# Tuning Decomposition Temperature: A Structural Study of Ligand Directed Bonding and Fluxionality

**DOI:** 10.1002/chem.202500178

**Published:** 2025-03-21

**Authors:** Shreya Mrig, Petra Vasko, Salma Saeed, Abil E. Aliev, Kersti Karu, Caroline E. Knapp

**Affiliations:** ^1^ Department of Chemistry University College London 20, Gordon Street London WC1H 0AJ; ^2^ Department of Chemistry University of Helsinki P.O. Box Helsinki, 55 00014 Finland

**Keywords:** aluminum, precursor, TGA, fluxionality, thiourea

## Abstract

In the first study of its kind towards the design and synthesis of easy‐to‐handle aluminium precursors that decompose at temperatures <200 °C: informed ligand choice and structural design of the compounds has caused inbuilt fluxionality leading to a markable decrease in the onset of decomposition temperatures. Eight thiourea ligands [**L** ^1^ **H**–**L** ^8^ **H**] were chosen with the steric bulk on the N atoms of these ligands varied systematically [**L** ^1−4^ **H**: RN(H)CS(NMe_2_) and **L** ^5−8^ **H**: RN(H)CS(NEt_2_); R=Me (**L** ^1^ **H** and **L** ^5^ **H**), Et (**L** ^2^ **H** and **L** ^6^ **H**), ^i^Pr (**L** ^3^ **H** and **L** ^7^ **H**) and Ph (**L** ^4^ **H** and **L** ^8^ **H**). Three families of aluminium compounds were synthesised by the reaction of these thiourea ligands with trimethylamine alane [Al(L^x^)_3_ (**1**–**7**), trimethylaluminium [MeAl(L^x^)_2_] (**8**–**11)** and triethylaluminium [EtAl(L^x^)_2_] (**12**–**14)** respectively. The three most spatially encumbered compounds (Al(L^3^)_3_ (**2**), Al(L^6^)_3_ (**5**) and Al(L^7^)_3_ (**6**) are highly fluxional in solution and displayed lengthening of the Al−N bond as compared to the other compounds. Both factors directly affect the activation temperature of these compounds. The remaining compounds were not shown to display any of these behaviours and consequently have higher thermal decomposition temperatures. SCXRD, ^1^H and ^13^C{^1^H} NMR, variable temperature ^1^H NMR, MS and EA have been used to study the structure and solution dynamics of **1–14**. This has directly been linked to the decomposition profiles of the compounds to assess their viability as precursors, evidencing that what we see in the solution state is present in the solid state too. Density functional theory calculations have been carried out to elucidate the various bonding modes observed for compounds **1–7**. Tandem MS has been employed to better understand the breakdown of the molecules.

## Graphical abstract

In this work, we have investigated the design and synthesis of aluminium compounds via systematic variation of ligand type. This is the first example of a ligand directed design and study of aluminium compounds which decompose at low temperatures (<200 °C). Their dynamic properties have also been investigated, with a focus on the decomposition profiles.

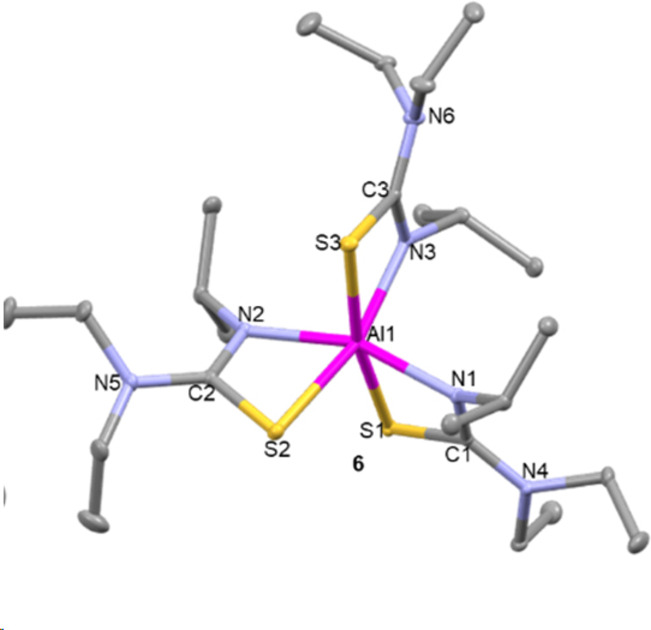

## Introduction

The manufacturing industry is increasingly seeing a shift towards manufacturing processes that are cheap, environment friendly and minimize energy usage.^[1]^ Device fabrication is one such industry that is seeing a push towards newer fabrication processes for printed electronics. A major shift in this industry is the move from using photolithography to various printing techniques like inkjet printing,[^2^, ^3^] spray coating,^[4]^ etc. To capitalize on these printing technologies, suitable solutions of metal precursors that allow deposition of the target material at low temperature are essential. Coordination metal complexes are commonly used for this objective.^[5]^ Suitable precursors have been synthesized and used for the deposition of silver[^6^, ^7^, ^8^, ^9^] and copper[^2^, ^10^, ^11^, ^12^] metals. However, there is a dearth of literature surrounding the synthesis and decomposition studies of aluminium compounds that have vast scope as precursors. As a cheap earth abundant metal^[6]^ which is also the fourth least resistive metal^[7]^ there is a lot of precedence to study aluminium precursors.

Most of the work in the field of aluminium deposition in recent years has been carried out by Lee et al.^[13]^ who used aluminium trihydride dibutylethearate (AlH_3_{O(C_4_H_9_)}_2_) as the aluminium precursor and deposited aluminium films on various substrates such as glass, polyethyleneterephthalate (PET), and various kinds of paper.^[14]^ Their work also lists the use of titanium isopropoxide as catalyst. Large area aluminium film deposition at room temperature over a span of 20 min was also reported by them.^[15]^ Lee et al.^[16]^ also synthesized a trimethylamine alane (TMAA) powder precursor which could be stored at −10 °C for up to 180 days. The ink could be formulated by dissolving this powder in a suitable solvent. The use of inks synthesized from this powder resulted in aluminium films with good mechanical properties when deposited through the solution stamping process. An additional use of TMAA to deposit aluminium has been in the work by Shen et al.^[17]^ However, their process makes use of an expensive platinum catalyst and high boiling point amines in addition to an adhesive layer which was annealed at 600 °C. Most recently, Douglas et al.^[18]^ used as synthesized (without dilution) triethylaminealane and dimethylethylaminealane as aluminium precursors. These precursors were drop cast onto different substrates at 100 and 120 °C to obtain conductive aluminium films.

While advances are being made in the aluminium deposition literature, a major roadblock in the field of printed electronics has been the trial‐and‐error attempts at ink formulations. Additionally, due to the highly unstable nature of the aluminium precursors, all depositions must be carried out in a glovebox. This represents a significant challenge to the field, namely the need to produce compounds that can be handled for processing in industrially viable environments at low temperatures.

Therefore, there is an urgent need for precursors that are designed through an informed and comprehensive study of the fundamental chemistry to make future designs more industrially viable.[^19^, ^20^, ^21^, ^22^] To the best of the authors’ knowledge, no study of this kind has been carried out for Al precursors and only a couple of examples of these exist in the copper deposition literature.[^2^, ^23^]

The current requirements of the market call for two essential criteria to be fulfilled‐ low temperature decomposition and easy air handling of aluminium precursors. These are largely dependent on the chemical and structural properties of the precursor molecules, which in turn are affected by the ligand choice.^[24]^ Given the highly reactive nature of aluminium, for low‐temperature decomposition of aluminium precursors a balance must be achieved such that the metal‐ligand bond is strong enough to stabilize the metal centre and allow easy air handling while simultaneously being weak enough to prevent thermal decomposition temperatures being increased beyond the desired limit. As a stepping stone towards successful aluminium thin film deposition *via* systematically designed precursors – we aimed to design and synthesise aluminium compounds that display the ability to be broken down at temperatures <200 °C while being easy to handle without the need for an inert atmosphere.

Polydentate ligands have often been used in literature to increase metal complex stability. Six‐membered chelating metal complexes are particularly widespread in precursor chemistry.[^25^, ^26^, ^27^] This works studies how the coordination of four‐membered chelating thiourea ligands to the aluminium ion affects the decomposition properties of the compounds. It is expected that a strained four‐membered chelate structure will result in a lower decomposition temperature as compared to a more stable six‐membered chelate ring. Additionally, the thiourea ligands display the presence of one donor atom which is a hard base (N) and another which is a soft base (S). It is expected that the hard base would coordinate strongly and quickly to the hard acid aluminium centre, while the bond with the soft base will be weaker. This will bring us closer to achieving the aforementioned balance. Thiourea ligands have also been previously shown to yield complexes that display fluxionality. It is hypothesized that fluxionality in these complexes would help reduce decomposition temperatures as molecules would be more likely to break apart during the process.^[28]^ Ease of synthesis, air and water stability as well as easy tunability on the N atoms makes these monoanionic bidentate molecules attractive ligands.^[29]^

Taking inspiration from Sullivan et al.,^[30]^ Bhide et al.^[28]^ and Ahmet et al.^[31]^ we used eight thiourea ligands and explored the changes that the different ligands cause to the thermal decomposition properties of the resultant aluminium compounds.

Herein, we describe the synthesis of novel aluminium‐thioureide compounds belonging to three families [Al(L^x^)_3_] (**1**–**7**), [MeAl(L^x^)_2_] (**8**–**11**) and [EtAl(L^x^)_2_] (**12**–**14**). These have been synthesised by using trimethylamine alane, trimethylaluminium and triethylaluminium as the sources of aluminium respectively. The structure, solution dynamics and thermal decomposition properties of these compounds have been studied and trends observed as both, the aluminium source and the alkyl groups on the N atoms of the thiourea ligand are altered. Variable temperature (VT) ^1^H NMR has been used to elucidate the fluxionality of the most sterically and spatially encumbered compounds. Trends between this fluxionality and the thermal decomposition properties of these complexes via thermal gravimetric analysis (TGA) have also been determined. Lastly, the thermal decomposition profiles of the hexa‐coordinate and penta‐coordinate compounds are discussed separately and in comparison, with each other.

## Results and Discussion

**Figure 1:**
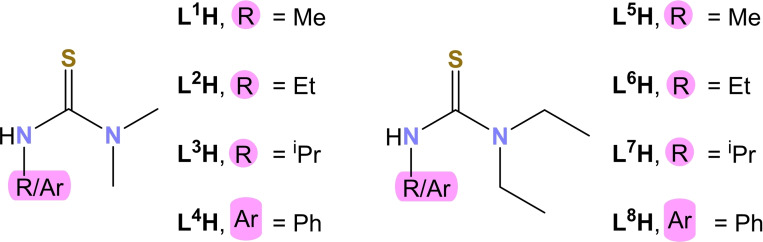
Structures of the thiourea ligands (**L^1^H**–**L^8^H**) synthesized by suitable variations to the synthesis method by Sullivan *et al*.^[30]^

Thiourea ligands with varying R groups on the N atoms were synthesized. The R groups were varied systematically in order of increasing steric bulk to gain a better understanding of how changing the steric bulk changes the properties of the final metal‐organic compounds, specifically their thermal decomposition properties and solution dynamics. Ligands **L** ^1^ **H** – **L** ^8^ **H** (Figure 1) were synthesized by suitable modifications to the standard synthetic routes by Sullivan *et al*.^[30]^ All ligands were synthesized by the addition reaction of methyl/ethyl diamine and the suitable isothiocyanate. Washing the products with excess hexane and drying them under vacuum yielded the thioureas in high purity and yield.[^32^, ^33^, ^34^]

**Scheme 1:**
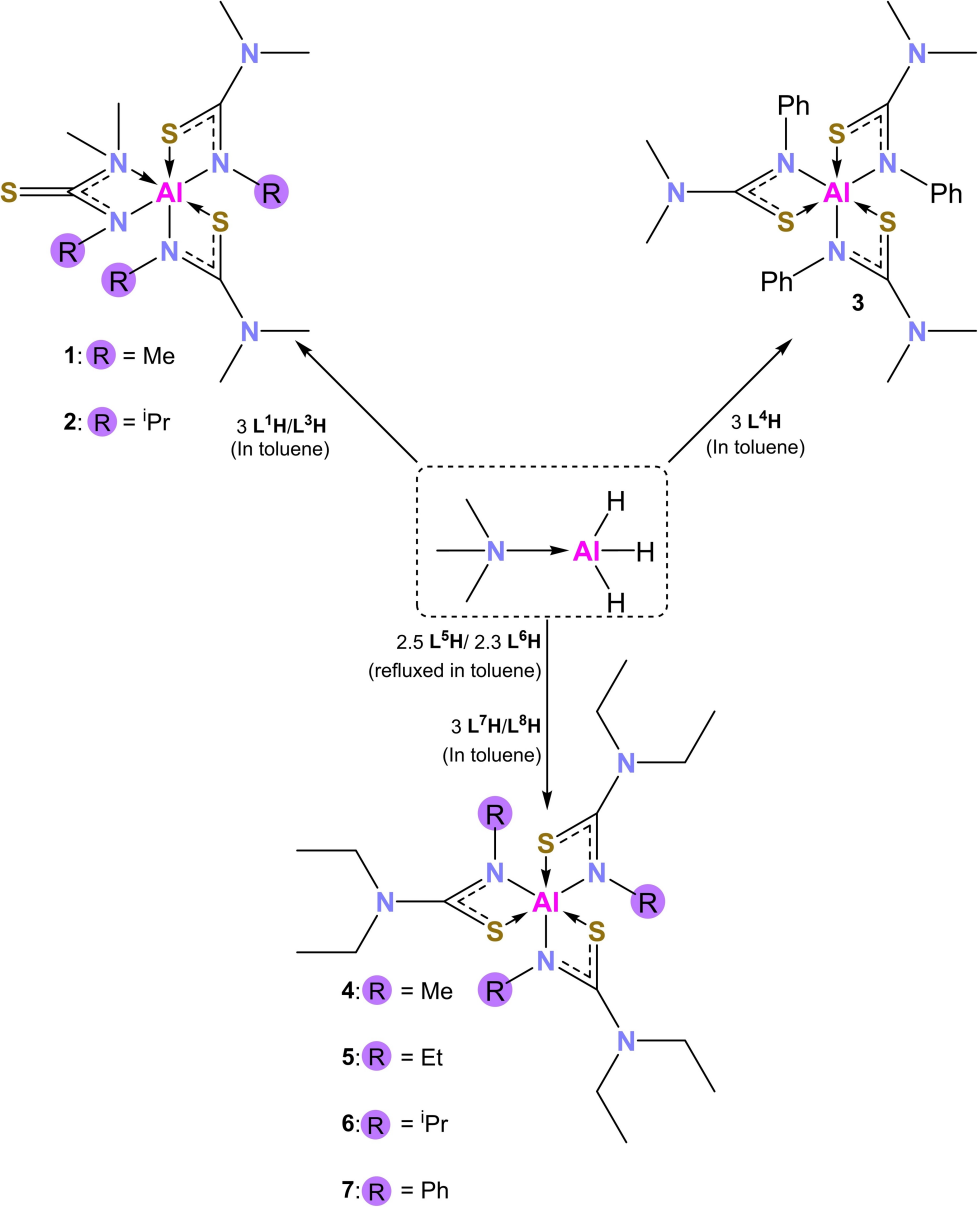
Synthetic routes towards the [Al(L^x^)_3_] compounds (**1**–**7**).

**Synthesis of tris(thioureide) aluminium(III) [Al(L** ^x^ **)_3_] compounds**: Seven novel tris(thioureide) aluminium(III) complexes of the type [Al(L^x^)_3_] were synthesised by the reaction of trimethylamine alane with the thiourea ligands under varying conditions (Scheme 1). Compound **4** could not be obtained by a 3 : 1 reaction of the ligand with TMAA. A 3 : 1 reaction yielded a mix of reagents and **4**, suggesting some degree of equilibrium. Similar results were seen when trying to synthesise **5**. Hence reaction conditions had to be altered to shift the equilibrium to the right and obtain the pure product. Compounds of the type Al(L^x^)_3_ were obtained with x=1, 3–8 (**1**–**7**). All of these were obtained as octahedral complexes, consisting of three thiourea ligands bound to the aluminium ion in a bidentate manner. Four‐membered chelate rings are formed by each of the bidentate ligands, exerting high steric strain and yielding compounds wherein large deviations from ideal octahedral geometry are observed. Inconsistent with expectations, **1** and **2** are heteroleptic octahedral aluminium compounds.

**Figure 2:**
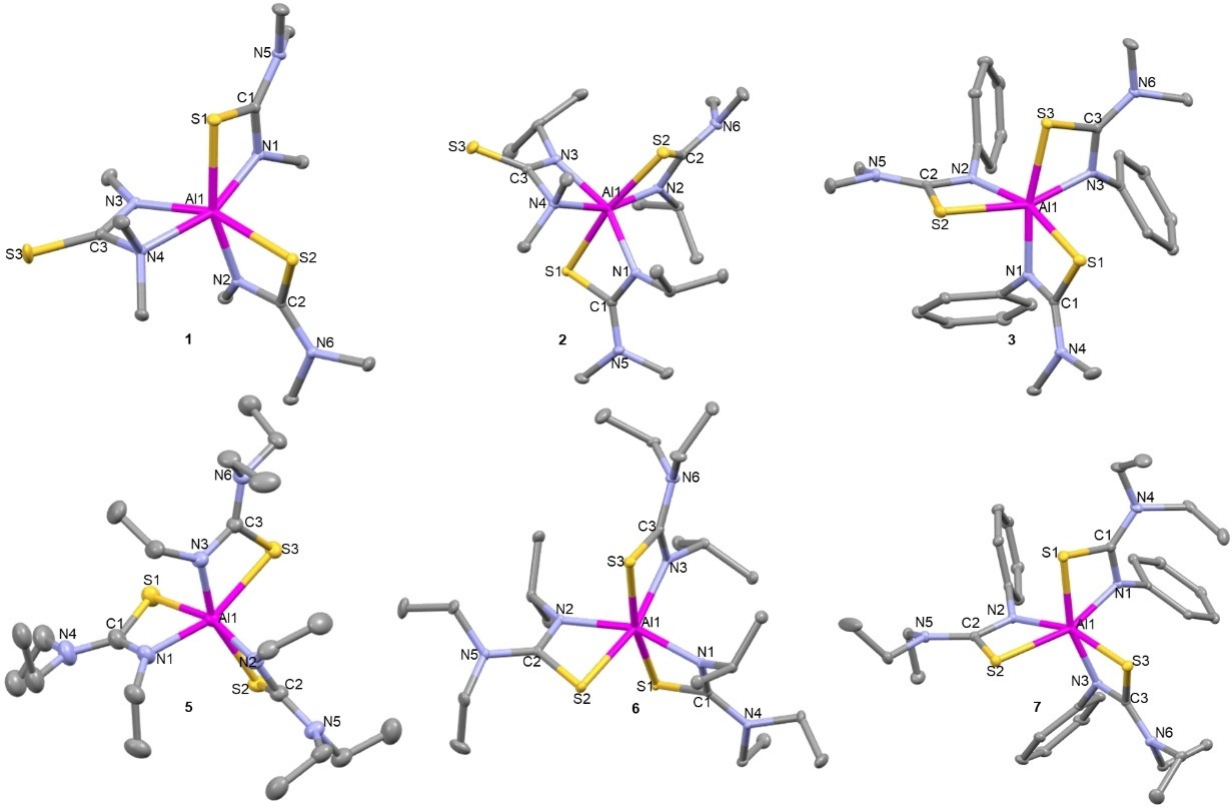
Solid state structures of compounds **1–7**. Thermal ellipsoids drawn at 20 % probability. Hydrogen atoms omitted for clarity, 2^nd^ molecule of **2** also omitted for clarity.

**Solid state studies of compounds 1–7**: Compound **1** crystallises in the monoclinic space group *P*2_1_/*c* as a cis isomer. The aluminium adopts a highly distorted octahedral geometry with three bidentate chelating ligands bound to it. (Figure 2). Two of the ligands in **1** display κ^2^ S−C−N bonding, while one of the ligands displays κ^2^ N−C−N bonding. This is inconsistent with expectations as it was hypothesized that all three ligands would display κ^2^ S−C−N chelation. However, this bonding mode is made possible by the delocalized electrons in the ligand backbone.^[35]^

This bidentate chelation via both N atoms of a thioureide ligand has been previously observed in literature,^[30]^ albeit sparsely. In this case, it is likely a result of aluminum's higher affinity for the harder N base, which is made possible by the low steric bulk (methyl groups) on the N atom. This difference in binding modes is also therefore, expected to affect the decomposition profile of this complex as the stronger bond between the hard Al acid and hard N base might result in elevated decomposition temperatures. Ligands chelating in the S−C−N binding mode exhibiting a delocalized π‐electron system. This is true for all tris thioureide aluminium complexes discussed in this section. The N−C−N bonded ligand has a carbon‐sulfur multiple bond character as reflected by the C−S bond lengths: S3−C3 (1.671(2) Å) bond is shortened in comparison to S1−C1 (1.742(2) Å) and S2−C2 (1.753(2) Å). Three of the Al−N bonds are similar to each other (1.935(1)‐1.951(1) Å), while the Al−N4 bond is elongated (2.113(1) Å). This is a result of the N4−Al bond being dative as opposed to the other N−Al bonds and acts to reduce the steric strain at the centre given the presence of the two methyl groups on N4. As expected, the dative S−Al bonds are longer than the N−Al bonds and agree with the typical Al−S bond length in a four‐membered chelate ring structure (S1−Al: 2.4168(6) and S2−Al: 2.42175(5) Å). The bite angles for the S−C−N κ^2^ ligands are as expected (~70°) while the bite angle for the N−C−N κ^2^ ligand is smaller at 65.77(5)°.

With an additional N−Al bond, and small alkyl substituents on the thiourea ligand, this is expected to be the compound with the least steric strain and consequently have a higher decomposition temperature than its counterparts.

Compound **2** crystallises in the *P*2_1_/*n* space group, with a structure similar to compound **1**. As with **1**, one of the three ligands displays κ^2^ N−C−N bonding, while the other two showcase κ^2^ S−C−N bonding. Of all the hexa‐coordinated complexes reported in this work, **2** is the only compound which shows the presence of two molecules in a unit cell (Z′=2). There are no significant differences in the bond lengths and angles between the two molecules (SI). In the solid state, **2** exists as a trans isomer. As with **1**, the S3−C3 bond (1.683(2) Å) is much shorter compared to the S−C bond distances of the S−C−N κ^2^ bound ligands (S1−C1: 1.745(2) Å and S2−C2: 1.737(2) Å). The N3−C3 bond length (1.301(2) Å) lies between the values for a single and double bond, indicating some level of delocalisation in the S3−C3−N3 fragment, while the N4−C3 bond (1.481(2) Å) is typical of a single C−N bond. As expected, the N−C−N κ^2^ ligand displays two different Al−N bond lengths, N4−Al=2.158(1) Å and N3−Al=2.001(1) Å. The N−Al bond lengths for the S−C−N κ^2^ ligands for **2** are longer than those seen in literature for analogous thioureide complexes.^[36]^ This is due to the strategic employment of the sterically bulky isopropyl groups which leads to high steric strain causing the metal ligands bond to lengthen and weaken – and is consequently expected to display lower decomposition temperatures. The S−Al bond lengths are in the expected range (2.3906(6) and 2.3911(6) Å). The octahedral arrangement is highly distorted from ideal geometry. This is reflected in the bond angles which deviate from the ideal 90° and 180° angles. The smallest bite angle for 1 is the N3−Al−N4 angle (64.77(5)°). This is possibly to maximize the distance between the sterically hindered N4, the Al ion and the neighbouring ligand. The other bite angles are in keeping with other complexes containing S atoms in four‐membered chelate ring structures (70.56(4)° and 70.93(4)°). The higher steric bulk which has probably caused the lengthening of the Al−N bonds is expected to be a major factor in helping reduce the decomposition temperature of **2**. However, the N−C−N κ^2^ will also probably drive this to be slightly higher than if all three ligands were seen to bond in the S−C−N κ^2^ mode.

**3** crystallises in the monoclinic space group Cc as a fac isomer No κ^2^ N−C−N bond was seen for any of the ligands, all three ligands were seen to bind in the S−C−N κ^2^ mode. This is in spite of the less bulky methyl group substituents on the N atom. It is expected that the bulkier phenyl groups make it harder for N−C−N κ^2^ bonding to take place. This trend is seen for compounds **3–7**, all of which have bulkier R/Ar groups attached to the N atoms as compared to compounds **1** and **2**. It is expected that as the ligand bulk increases, steric strain supersedes aluminium's affinity for the harder N base and prevents any κ^2^ N−C−N bonding.

The bond angles and bond lengths for **3** are in keeping with those seen for the other S−C−N κ^2^ bound ligands. The Al−S bond lengths lie in the range 2.399(1)‐2.4399(1) Å, while the Al−N bond lengths are also as expected, ranging between 1.962(2)‐1.973(2) Å. The S−Al−N bite angles all lie close to 70°. With all ligands displaying S−C−N κ^2^ bonding, the Al−S bonds are expected to break off easily pushing the decomposition temperature lower. However, the breakdown of the phenyl ring might cause this to shift in the opposite direction.

Crystals of **4** were found to be inherently twinned and a complete SCXRD analysis could not be carried out to obtain a full data set. However, the structure of a unit cell was determined using the Rigaku ‘What is this’ tool, which allows for the fast collection of a minimal date set. This confirmed the formation of **4** (although bond lengths and angles are not of sufficient quality to be deemed useful) as a distorted octahedral complex, with three S−C−N κ^2^ ligands bound to the aluminium ion, as seen for **3**.

**5**, **6** and **7** are homoleptic octahedral compounds that crystallise in *P*2_1_/*n*, *P*2_1_/*c* and *P*2_1_/*c* space groups respectively. **5** and **7** exist as fac isomers while **6** is the only octahedral compound that occurs as a mer isomer in this series. Al−S bond lengths for all three compounds are in the expected range and the bite angles are all close to 70°. **6** however shows deviations in the Al−N bond lengths, which like **2** are longer than its analogous counterparts. The presence of the ethyl groups instead of the methyl will impact the decomposition temperatures of these compounds. The strategic employment of the spatially cumbersome isopropyl groups causes the Al−N bonds of **6** to be longer than its analogues – which will help decrease its decomposition temperature.

Crystallographic data and selected bond angles and lengths for compounds **1–7** are given in tables 1 and 2.

**Table 1:** Crystallographic data for compounds **1**–**3** and **5**–**7**.

Compound	**1**	**2**	**3**	**5**	**6**	**7**
Empirical formula	C_12_H_27_AlN_6_S_3_	C_36_H_78_Al_2_N_12_S_6_	C_27_H_33_AlN_6_S_3_	C_21_H_45_AlN_6_S_3_	C_24_H_51_AlN_6_S_3_	C_33_H_45_AlN_6_S_3_
Formula weight/g mol^−1^	378.55	925.42	564.75	504.79	546.86	648.91
Crystal system	Monoclinic	Monoclinic	Monoclinic	Monoclinic	Monoclinic	Monoclinic
Space group	*P*2_1_/*c*	*P*2_1_/*n*	*Cc*	*P*2_1_ */n*	*P*2_1_/*c*	*P*2_1_/*c*
*a*/Å	7.36380(10)	11.54960(10)	16.7376(2)	11.17500(10)	17.1166(2)	10.21000(10)
*b*/Å	13.4783(2)	22.9737(2)	9.72850(10)	14.44600(10)	10.07450(10)	18.4988(2)
*c*/Å	19.2786(3)	18.8965(2)	17.8234(2)	18.1727(2)	19.6806(2)	18.2633(2)
*α*/°	90	90	90	90	90	90
*β*/°	92.8350(10)	96.2350(10)	102.7480(10)	98.1450(10)	113.3400(10)	92.4090(10)
*γ*/°	90	90	90	90	90	90
Volume/Å^3^	1911.09(5)	4984.28(8)	2830.68(6)	2904.10(5)	3116.03(6)	3446.39(6)
*Z*	4	4	4	4	4	4
*ρ*_*calc*/_g cm^−3^	1.316	1.233	1.325	1.155	1.166	1.251
μ/mm^−1^	4.032	3.180	2.913	2.766	2.613	2.456
Reflections collected	26510	92580	23220	72066	47141	48809
Independent reflections	3801	8814	5549	5105	5480	6908
R_int_	R_int_=0.0601	R_int_=0.0485	R_int_=0.0291	R_int=_0.0366	R_int_=0.0380	R_int_=0.0423
Final *R* indexes [*I* ≥2*σ* (*I*)]	R_1_=0.0341, wR_2_=0.0923	R_1_=0.0314, wR_2_=0.0792	R_1_=0.0234, wR_2_=0.0626
	R_1_=0.0367, wR_2_=0.0997	R_1_=0.0278, wR_2_=0.0763	R_1_=0.0394, wR_2_=0.0992
Final *R* indexes [all data]	R_1_=0.0374, wR_2_=0.0962	R_1_=0.0363, wR_2_=0.0825	R_1_=0.0236, wR_2_=0.0630
	R_1_=0.0405, wR_2_=0.1025	R_1_=0.0284, wR_2_=0.0770	R_1_=0.0453, wR_2_=0.1064
Data/restraints/ parameters	3801/0/208	8814/0/529	5549/2/340
	5105/245/386	5480/0/319	6908/0/394
Goodness‐of‐fit on *F* ^2^	1.068	1.035	1.034	1.082	1.049	1.042
CCDC Number	2257998	2257999	2258000	2258001	2258002	2258003

**Table 2:** Selected bond angles and bond lengths of compounds **1–3** and **5–7**.

Compound	**1**	**2**	**3**	**5**	**6**	**7**
Length (Å)						
Al−N1	1.945(1)	2.000(1)	1.970(2)	1.955(2)	1.9932(9)	1.966(1)
Al−N2	1.935(1)	1.995(1)	1.962(2)	1.949(2)	1.999(1)	1.980(2)
Al−N3	1.951(1)	2.001(1)	1.973(2)	1.958(2)	2.034(1)	1.971(1)
Al−N4	2.113(1)	2.158(1)	–	–	–	–
Al−S1	2.4168(6)	2.3906(6)	2.4121(8)	2.4368(8)	2.3607(4)	2.4428(7)
Al−S2	2.4217(5)	2.3911(6)	2.4399(8)	2.4120(7)	2.4572(6)	2.4043(7)
Al−S3	–	–	2.399(1)	2.4235(7)	2.4095(4)	2.4047(7)
S3−C3	1.671(2)	1.683(2)	–	–	–	–
						
Angle (°)						
S1−Al−N1	70.55(4)	70.56(4)	69.94(6)	69.52(6)	71.20(3)	69.40(4)
S2−Al−N2	70.61(4)	70.93(4)	69.77(6)	69.95(5)	69.02(3)	70.16(5)
S3−Al−N3	–	–	70.53(6)	69.75(5)	69.87(3)	70.37(4)
N3−Al−N4	65.77(5)	64.77(5)	–	–	–	–
S1−Al−S2	–	168.50(2)	–	–	–	–
S1−Al−S3	–	–	–	–	169.05(2)	–
S1−Al−N2	162.48(4)	–	–	–	–	–
S3−Al−N1	–	–	168.31(7)	160.08(6)	–	–
S1−Al−N3	–	–	–	–	–	163.33(5)

**Figure 3:**
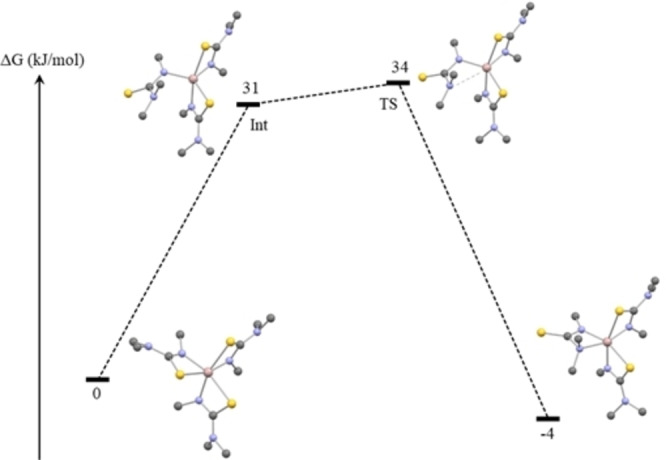
Calculated mechanism for the switch in ligand bonding mode for compound **1**. Colour codes: Al pink, N blue, S yellow and C grey. Hydrogen atoms omitted for clarity.

**Density Functional Theory Studies of Compounds 1–7**: DFT studies were employed to study the different bonding modes observed in compounds **1–7**. The primary aim of these studies was to shed greater light on the preference for either S−C−N or N−C−N κ^2^ binding displayed by the different ligands (**L** ^1^ **H**, **L** ^3^ **H** – **L** ^8^ **H**). All seven compounds were optimised using the dispersion corrected functional PBE0‐GD3BJ and Def2‐TZVP basis set for all atoms using the programme Gaussian16.62–67 In addition, a solvent model (pcm, solvent=toluene) was applied to simulate experimental conditions more accurately. We began by optimising the pseudo‐octahedral structures **1–7** as the experimentally observed two different isomers. The relative stabilities of the two calculated isomers for each compound were in excellent agreement with the experimentally observed structures as can be seen from Table 3. The calculations confirm that for the compounds **1** and **2**, which carry the smallest substituents on the nitrogens, the preferred bonding mode of the third ligand is N−C−N κ^2^. Unsurprisingly, there is clear correlation between the size of the substituents and preference for all three ligands to bind in a S−C−N fashion as the substituent size increases. The most favoured S−C−N bonding mode is calculated for the most sterically hindered compound **7** which bears ethyl and phenyl substituents on the nitrogens. However, for **1**, the compound with only methyl substituents and thus the least steric hindrance between the three ligands around the aluminium ion, the calculated Gibbs free energy difference is only 4.5 kJ mol^−1^ between the two isomers. Hence, we investigated the possible mechanism for the switching of the bonding modes in this compound further (Figure 3). We were able to locate a local minimum structure, which is 31 kJ mol^−1^ higher in energy compared to the 3 S−C−N κ^2^ bound structure. In this intermediate, two ligands are S−C−N κ^2^ bound to aluminium and the third ligand is bonded to Al only from one nitrogen atom. A transition state for the binding of the second nitrogen atom to the aluminium ion was found only 3 kJ mol^−1^ above the intermediate. As a conclusion, the calculation corroborates experimental findings well as it is evident that only the Me‐substituted ligands can bind through the harder N‐donor due to higher steric demands of bigger substituents.

**Table 3:** Gibbs free energy differences for the two calculated isomers of compounds **1–7**.

Compound	3 S−C−N (a.u.)	N−C−N, 2 S−C−N (a.u.)	ΔG (kJ mol^−1^)
**1**	−2237.802954	−2237.804667	4.5
**2**	−2473.321399	−2473.329684	21.8
**3**	−2812.455923	−2812.448866	−18.5
**4**	−2473.341096	−2473.327179	−36.5
**5**	−2591.109352	−2591.09578	−35.6
**6**	−2708.865417	−2708.852675	−33.5
**7**	−3047.996974	−3047.970201	−70.3

**Scheme 2:**
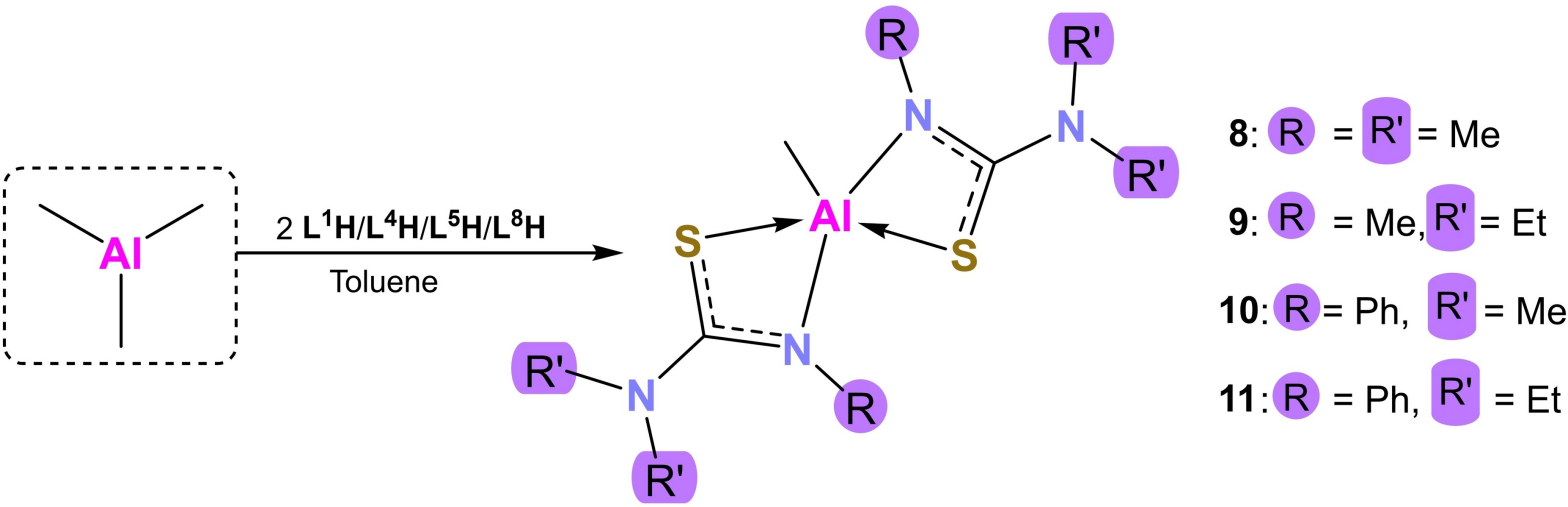
Schematic showing the formation of the bis(thioureide) aluminium methyl compounds **8**–**10**.

**Synthesis of Bis(Thioureide) Aluminium Methyl [MeAl(L** ^x^ **)_2_] and Bis(Thoiureide) Aluminium Ethyl Compounds [EtAl(L** ^x^ **)_2_]**:

To study the impact of moving from a hexa‐coordinate to a penta‐coordinate Al‐thioureide structure, compounds of the type [MeAl(L^x^)_2_] and [EtAl(L^x^)_2_] were synthesised. Ligands **L** ^1^ **H** – **L** ^8^ **H** were reacted with trimethylaluminium in a 2 : 1 ratio. However, in spite of various attempts not all of the reactions were successful. Whilst reactions with **L** ^1^ **H**, **L** ^4^ **H**, **L** ^5^ **H** and **L** ^8^ **H** yielded the desired products, reactions with the remaining ligands only produced intractable mixtures. Scheme 2 highlights the synthesis of compounds **8–11**. All four reactions yielded pentacoordinate aluminium(III) compounds with distorted geometries that were assessed using the structural parameter (τ_5_).^[24]^ As with the hexacoordinate compounds, the presence of strained four‐membered ring chelates causes geometric strain resulting in these deviations.

**Figure 4:**
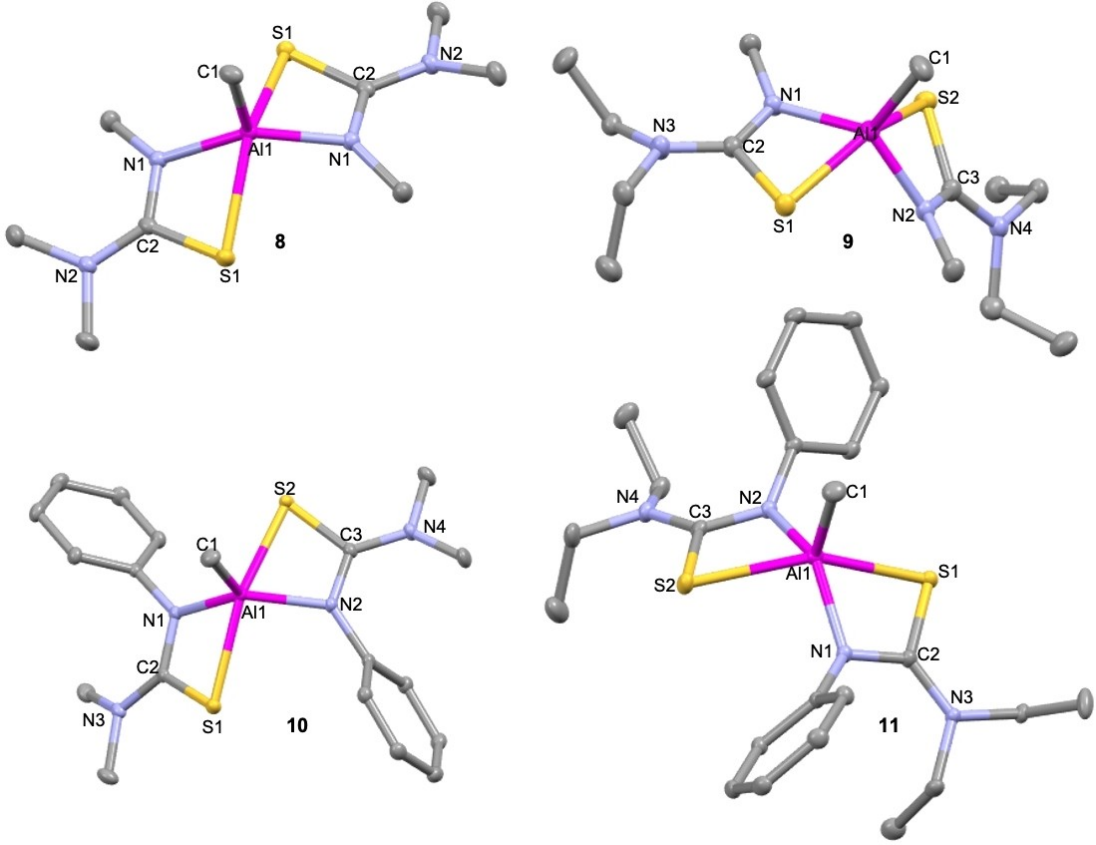
Solid state structures of compounds **8–11**. Thermal ellipsoids drawn at 20 % probability. Hydrogen atoms omitted for clarity. 2^nd^ molecule of **10** omitted for clarity.

Compound **8** crystallises in the monoclinic space group *C*2/*c* (Figure 4). The coordination around the aluminium ion is square planar (τ_5_=0.02) where the N and S atoms of the thioureide ligands are almost planar and form the base of the pyramid while the methyl C occupies the apical position. The bite angles and bond lengths lie in the expected range. The chelate rings are not puckered with the sum of angles (358.56°) almost equal to the ideal 360°. This shows the least deviation from ideal geometry of all the penta‐coordinate compounds, which is made possible by the small methyl substituents on the ligand. Unlike as seen for its tris(thioureide) counterpart (**1**), no κ^2^ N−C−N bound ligands are observed in spite of the low steric bulk on the ligand. This could be because the of the inductively electron donating effect of the methyl group which helps satiate the required electron density around the aluminium ion, preventing the need for N to bond to it (Tables 4 and 5).

**Table 4:** Crystallographic data of compounds **8–14**.

Compound	**8**	**9**	**10**	**11**	**12**	**13**	**14**
Empirical formula	C_9_H_21_AlN_4_S_2_	C_13_H_29_AlN_4_S_2_	C_45_H_58_Al_2_N_8_S_4_	C_23_H_33_AlN_4_S_2_	C_20_H_27_AlN_4_S_2_	C_14_H_31_AlN_4_S_2_	C_24_H_35_AlN_4_S_2_
Formula weight/g mol^−1^	276.408	332.50	893.19	456.63	414.55	346.543	468.64
Crystal system	Monoclinic	Monoclinic	Triclinic	Monoclinic	Orthorhombic	Monoclinic	Monoclinic
Space group	*C*2*/c*	*P*2_1_ */n*	*P* $\bar{1}$	*P*2_1_ */c*	*Pbca*	*P*2_1_ */n*	*P*2_1_ */c*
*a*/Å	9.8655(3)	15.1405(4)	11.29580(10)	13.15200(10)	12.0823(2)	17.2432(3)	13.83660(10)
*b*/Å	13.7896(3)	7.1913(2)	14.03850(10)	15.28990(10)	13.3175(2)	7.0913(1)	14.50950(10)
*c*/Å	10.9837(3)	17.8801(5)	16.1273(2)	13.47730(10)	26.9759(4)	17.7736(3)	14.26540(10)
*α*/°	90	90	73.2440(10)	90	90	90	90
*β*/°	107.631(3)	109.355(3)	77.3280(10)	114.2260(10)	90	117.748(2)	115.4130(10)
*γ*/°	90	90	89.6640(10)	90	90	90	90
Volume/Å^3^	1424.05(7)	1836.76(9)	2384.66(4)	2471.51(4)	4340.58(12)	1923.38(6)	2586.83(4)
*Z*	4	4	2	4	8	4	4
*ρ*_*calc*/_g cm^−3^	1.289	1.202	1.244	1.227	1.269	1.197	1.203
μ/mm^−1^	3.842	3.057	2.498	2.418	2.703	2.939	2.324
Reflections collected	13044	36888	60661	61057	97605	40038	61323
Independent reflections	1474	3756	9820	5180	4486	3933	5333
R_int_	0.0422	0.0821	0.0360	0.0373	0.0727	0.0367	0.0413
Final *R* indexes [*I* ≥2*σ* (*I*)]	R_1_=0.0376, wR_2_=0.0955	R_1_=0.0501, wR_2_= 0.1318	R_1_=0.0329, wR_2_=0.0842	R_1_=0.0310, wR_2_= 0.0819	R_1_=0.0385, wR_2_= 0.1240	R_1_=0.0359, wR_2_= 0.1046	R_1_=0.0315, wR_2_=0.0810
Final *R* indexes [all data]	R_1_=0.0404, wR_2_=0.0979	R_1_=0.0734, wR_2_= 0.1482	R_1_=0.0407, wR_2_=0.0877	R_1_=0.0346, wR_2_=0.0841	R_1_=0.0589, wR_2_=0.1402	R_1_=0.0394, wR_2_= 0.1079	R_1_=0.0332, wR_2_=0.0821
Data/ restraints/ parameters	1474/0/78	3756/0/211	9820/122/571	5180/0/292	4486/0/263	3933/0/214	5333/0/285
Goodness‐of ‐fit on *F* ^2^	1.041	1.030	1.070	1.066	1.035	1.056	1.049
CCDC Number	2258018	2258021	2258019	2258020	2258022	2258023	2258024

**Table 5:** Selected bond lengths and angles of compounds **8–14**.

Compound	**8**	**9**	**10**	**11**	**12**	**13**	**14**
Length (Å)							
Al−N1	1.924	1.908(2)	1.933(1)	1.931(1)	1.930(2)	1.928(2)	1.9371(9)
Al−N2	–	1.907(2)	1.922(2)	1.936(1)	1.940(2)	1.923(2)	1.938(1)
Al−S1	2.4318	2.441(7)	2.4315(6)	2.4327(6)	2.508(2)	2.4502(5)	2.4413(5)
Al−S2	–	2.479(1)	2.4639(6)	2.4534(5)	2.365(4)	2.4163(8)	2.4314(4)
Al−C1	1.969	1.954(3)	1.959(2)	1.959(2)	1.990(5)	1.973(2)	1.967(2)
							
Angle (°)							
S1−Al−N1	70.46	70.4(2)	70.61(4)	70.38(4)	69.38(8)	70.16(5)	70.14(3)
S2−Al−N2	–	69.97(7)	70.05(4)	69.86(4)	71.9(1)	70.68(5)	70.48(4)
S1−Al−C1	108.70	108.8(2)	104.68(6)	107.18(6)	100.3(1)	105.52(6)	107.84(5)
S2−Al−C1	–	103.2(1)	103.62(6)	108.11(6)	111.2(2)	106.50(6)	104.81(5)
S1−Al−S2	–	147.9(2)	151.69(2)	144.69(2)	148.5(1)	147.97(3)	147.34(2)
N1−Al−N2	–	128.2(1)	128.05(6)	133.61(5)	129.64(7)	134.55(7)	132.98(5)
S1−Al−S1	142.59		–	–	–	–	–
N1−Al−N1	141.16		–	–	–	–	–

**Figure 5:**
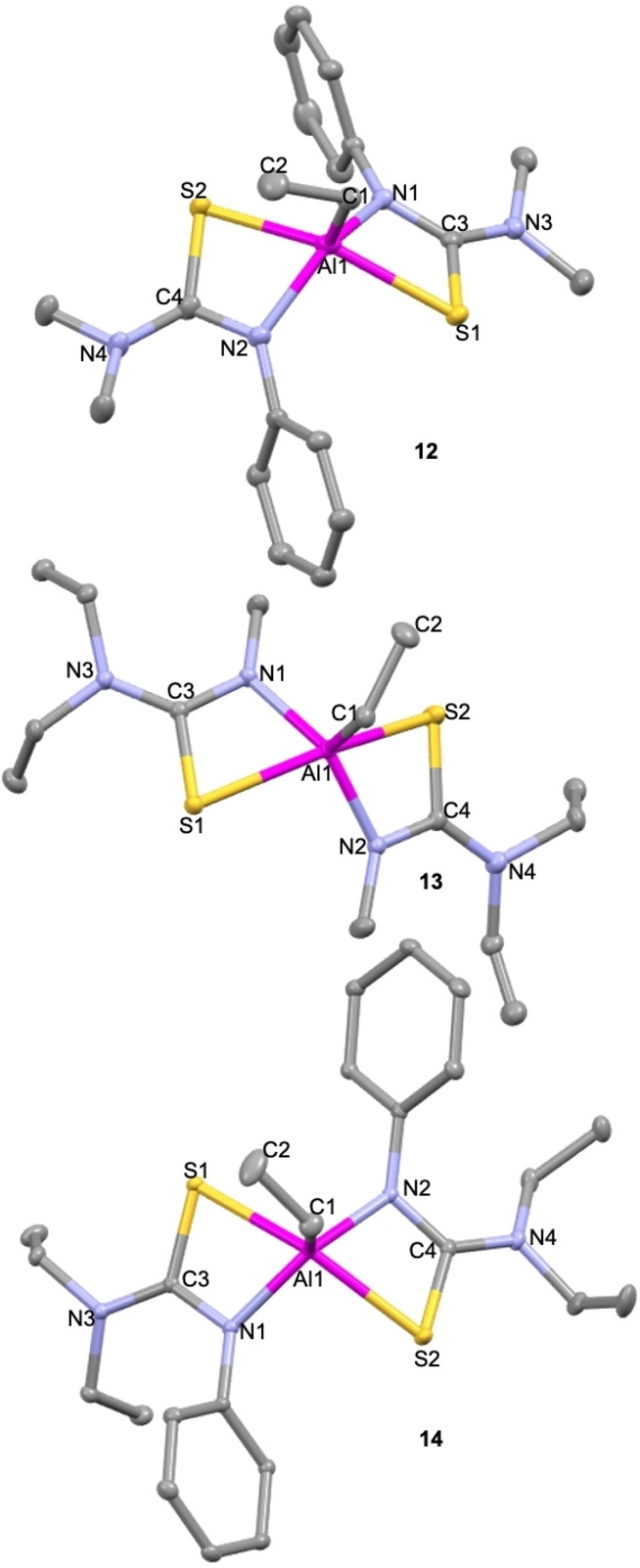
Solid state structures of compounds **12–14**. Thermal ellipsoids drawn at 20 % probability. Hydrogen atoms omitted for clarity.

**9**, **10** and **11** crystallise in the *P*2_1_/*n*, *P* $\bar{1}$ and *P*2_1_/*c* space groups as distorted square planar compounds with τ_5_=0.33, 0.40 and 0.18 respectively (Figure 5). These are isostructural to **8**. **10** shows the presence of two molecules in a unit cell (Z′=2) along with the presence of a toluene molecule. There are no significant differences between the two molecules in their bond angles and lengths. The chelate rings are not puckered in any of the three compounds, with the sum of internal angles almost a perfect 360°. The Al−N and Al−C bond lengths for **9** are smaller than those for its analogous counterparts. The Al−S bond lengths for all three compounds are within the expected range (Tables 4 and 5).

**Scheme 3:**
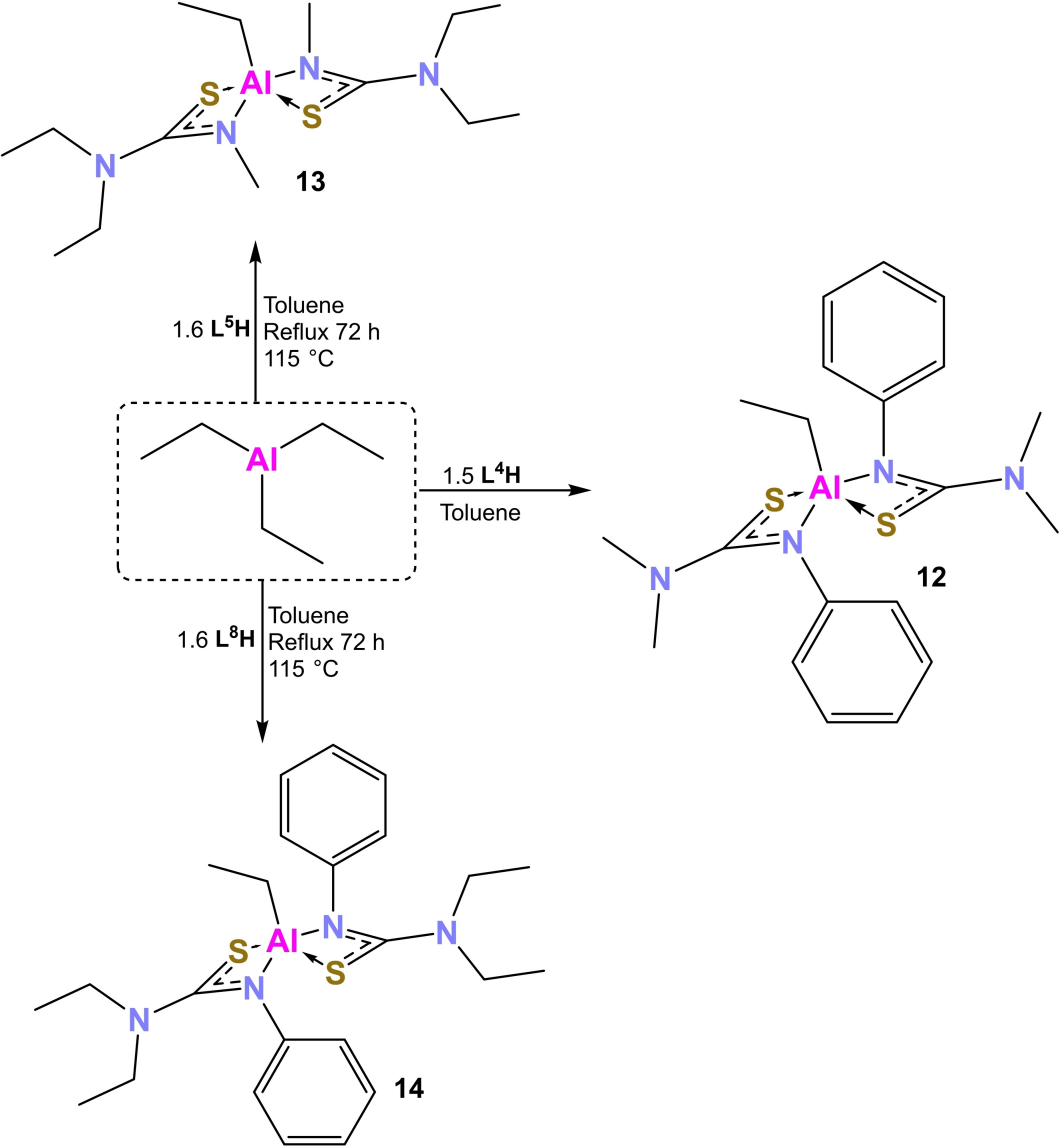
Schematic showing the synthesis of compounds **12**–**14**.

As with compounds **8–11**, attempts were made to synthesis [EtAl(L^x^)_2_] compounds by reacting triethyl aluminium with all 8 ligands. However, again, not all reactions were successful. **12–14** were isolated by the reaction of triethylaluminium with **L** ^4^ **H**, **L** ^5^ **H** and **L** ^8^ **H** respectively (Scheme 3). Unlike the synthesis of the aluminium methyl compounds, the synthesis of compounds **12–14** required multiple permutations and combinations as seen in Scheme 3. For the reaction with **L** ^4^ **H**, the 2 : 1 reaction yielded only the starting reagent: the thiourea pro‐ligand **L** ^4^ **H** in crystalline form. It was hypothesised that the equilibrium for this reaction lay largely to the left. To shift the equilibrium towards the right, the stoichiometric ratios of the reagents were varied systematically and a 1.5 : 1 ratio of **L** ^4^ **H** to triethylaluminium was found to be the ideal ratio to shift the equilibrium towards the right and recrystallisation from toluene yielded clear colourless crystals of **12**. For compound **13**, to shift the equilibrium to the right, a ligand:triethylaluminium ratio of 1.6 was required in addition to a 72 h reflux in toluene. However, even then, a mix of **L** ^5^ **H** and **13** crystals was obtained which could not be separated. **14** required similar conditions as **13** but a pure product with no mix of the ligand was obtained.

No significant variation is seen between the solid‐state structures of the three compounds. All of them are penta‐coordinate with two κ^2^ S−C−N bound thiourea ligands and an ethyl ligand. **12** crystallises in the orthorhombic space group *Pbca* and adopts a distorted square planar geometry (τ_5_=0.31). **13** crystallises in the monoclinic space group *P*2_1_/*n*, in a distorted square planar geometry (τ_5_=0.22). **14** also crystallises in a monoclinic space group: *P*2_1_/*c* in a distorted square planar geometry (τ_5_=0.24). In all cases, the ethyl group is in the axial position. The bite angles for all three compounds are similar to those seen for compounds **1–11**. Al−N bonds are shorter than Al−S bonds in keeping with expectations as the latter are datively bound while the former are coordinatively bound. Al−N bonds for **13** are shorter than those for **12** possibly due to the more flexible nature of the ligand substituents. None of the rings are seen to be puckered.

None of the penta‐coordinate compounds display κ^2^ N−C−N bonding of the thiourea ligands which could be explained to be a result of the inductively electron donating effect of the alkyl groups. This allows the aluminium ion to be more electronically saturated than in compounds **1–7**.

**NMR Studies of compounds 1–14**: The NMR spectroscopic data for most compounds are unexceptional, with the spectra displaying all the expected signals, integration of which confirms the formation of the expected product. No characteristic NH peak is seen either, confirming the complete conversion of the reagents to the desired product. For compounds **1**, **3**, **4**, **7**, **8**, **9**, **10**, **11**, **12** and **14**, the detailed NMRs are given in the SI. The ^1^H NMR spectrum of **13** is different to the rest as the pure product could not be isolated from a mix of the product and the ligand **L** ^5^ **H**. This is reflected in the NMR which shows peaks for both the ligand, including the NH peak as well as those for the product which are slightly shifted, indicating coordination to the metal ion. For the compounds with the highest steric strain and spatial occupation (**2**, **5** and **6**), the solution dynamics were seen to be very interesting and these are discussed here. These compounds display *in solutio* intramolecular rearrangement which provides further insights into their decomposition temperatures.

**Figure 6:**
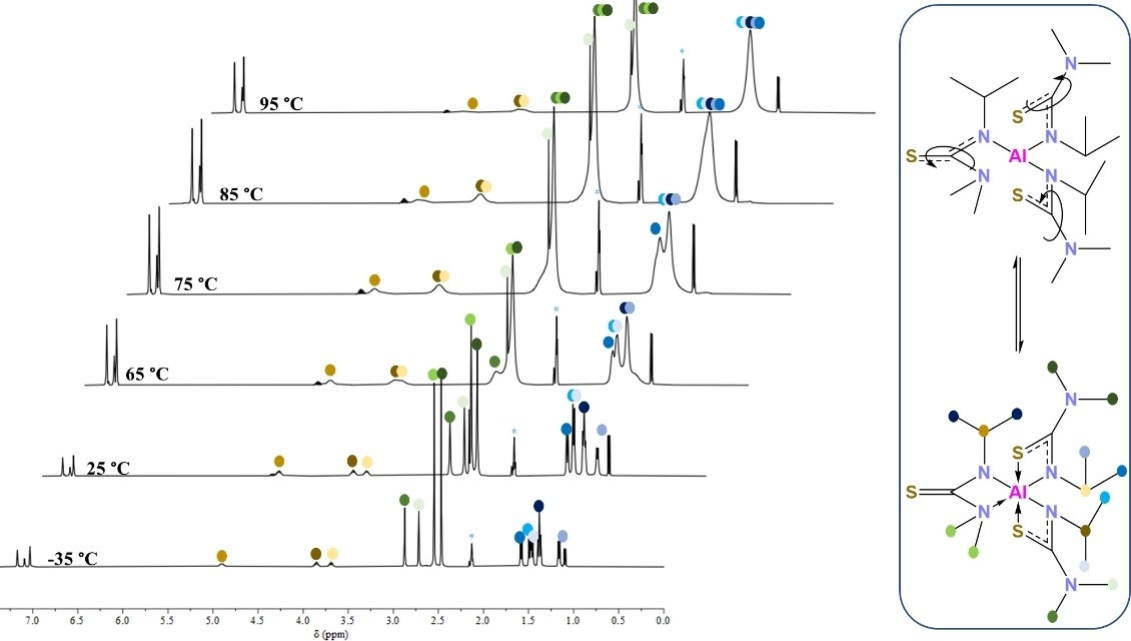
Variable temperature ^1^H NMR spectrum of **2** displaying fluxional behaviour of the compound.

The room temperature ^1^H NMR spectrum of **2** shows three distinct signals in a 1 : 1 : 1 ratio at 3.70, 3.86 and 4.87–4.78 ppm for each of the isopropyl C(*H*)(CH_3_)_2_ protons on the three ligands. Surprisingly, 5 sharp singlet peaks between 2.98–2.30 ppm are observed for the NMe_2_ protons, while the terminal isopropyl methyl groups occur as 6 separate doublets between 1.68–0.97 ppm, indicating all six CH_3_ groups are in inequivalent NMR environments. The occurrence of such a complex ^1^H NMR is indicative of inequivalent proton environments arising as a result of the spatial proximity of the terminal methyl groups on the isopropyl substituents in addition to *in solutio* intramolecular rearrangement. To gain a better understanding of this behaviour, variable temperature ^1^H NMR was carried out in toluene‐d_8_ (Figure 6). At lower temperatures (−35 °C) the peaks are seen to sharpen while slow increase of temperature causes these peaks to coalesce. However, even at the highest measured temperature (95 °C) complete coalescence of the signals is not seen, rather peaks get broader as the temperature is increased. This can be attributed to a labile behaviour of the ligands as seen for the dithiocarbamates^[37]^ and thioureide complexes with other metals[^28^, ^38^] which may be resulting in a change in isomerism for the compound. It is hypothesised that this occurs through a bond rupture‐rotation mechanism.^[39]^ Possibly due to the poor bonding between the hard Al acid and the weak S base, there is momentary rupture of the Al−S bond, rotation around the C−N bond followed by the formation of either an Al−S or an Al−N bond. At lower temperatures, this association‐disassociation is slow enough to be visible in the NMR timescale. However, as the temperature is increased, this occurs at a rate faster than the NMR timescale causing coalescence and broadening of peaks. No broad signal for the NH proton of the pro‐ligand was seen, confirming complete conversion to the desired compound.

For signals at 2.60–2.80 ppm due to N(C *H*_3_)_2_ protons, the coalescence temperature can be estimated to be 70±3 °C. Assuming that the observed exchange is between two sites with the population 2 : 1 and the chemical shift difference of 115±15 Hz in the absence of the exchange, the free energies of activation can be estimated to be ΔGA≠=70.8±1.1 kJ mol^−1^ (for the transition from the more populated site to the less populated site) and ΔGB≠=68.8±1.1 kJ mol^−1^ (for the transition from the less populated site to the more populated site) using equations by Shanan‐Atidi and Bar‐Eli.^[40]^ Similarly, for signals at 3.60–4.90 ppm due to NC(*H*)(CH_3_)_2_ protons the coalescence temperature can be estimated to be 100±5 °C. Assuming that the observed exchange is between two sites with the population 2 : 1 and chemical shift difference of 453±35 Hz in the absence of the exchange, the free energies of activation can be estimated to be ΔGA≠=73.0±1.3 kJ mol^−1^ and ΔGB≠=70.9±1.3 kJ mol^−1^.

**Figure 7:**
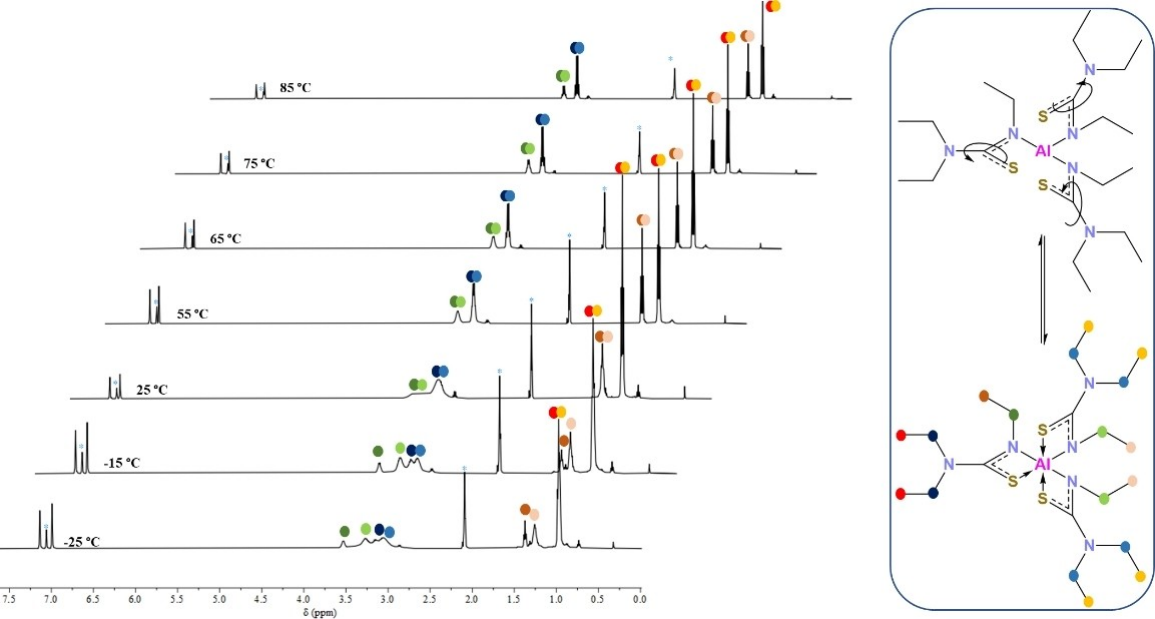
Variable Temperature ^1^H NMR spectrum of **5**, displaying functional behaviour of the compound.

The solution dynamics of **5** are found to be similar to those of **1**. The room temperature ^1^H NMR spectrum shows broad peaks instead of sharp signals (Figure 7). Two broad peaks are seen at 3.54 ppm and between 3.33–2.92 ppm in a 1 : 2 ratio which correspond to the N(C *H*_2_CH_3_) and N(C *H*_2_CH_3_)_2_ protons. The signals corresponding to the terminal methyl protons appear slightly sharper. A slightly broad triplet is seen for the N(CH_2_C *H*_3_) protons at 1.28 ppm while the N(CH_2_C *H*_3_)_2_ protons appear as a sharp triplet at 0.99 ppm. VT ^1^H NMR studies reveal results discussed previously; as the temperature is lowered, a more complex structure is seen, with inequivalent ligand environments observed. As the temperature is increased, the peaks begin to coalesce and with further temperature increase sharpen into two quartets and two triplets as expected for a structure wherein all the ligands are in equivalent NMR environments. This is indicative of any *in solutio* intramolecular occurring fast enough at these temperatures to not be captured by the NMR timescale. The peaks sharpening is seen at a lower temperature (75 °C) as compared to **2** and is indicative of slower solution dynamics possibly due to hinderance caused by the ethyl arms.

**Figure 8:**
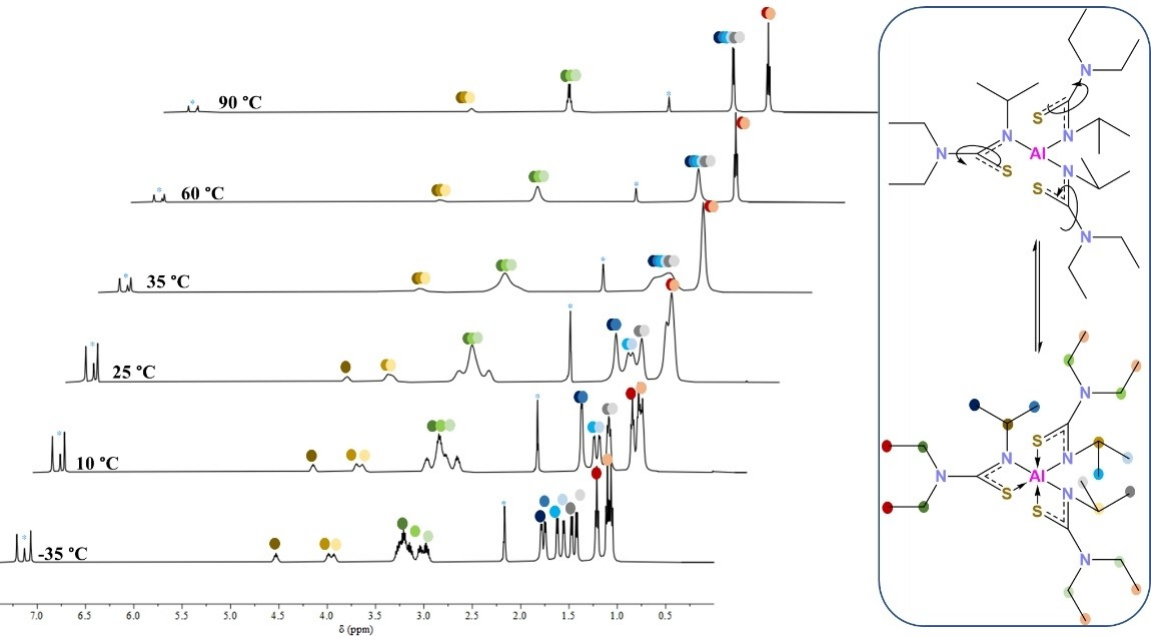
VT ^1^H NMR spectrum of **6** showing fluxional behaviour in solution.

Compound **6** is also found to be fluxional in solution. The room temperature ^1^H NMR of **6** shows two broad peaks in a 1 : 2 ratio at 4.40 and between 4.12–3.80 ppm corresponding to the three C(H)(CH_3_)_2_ protons on the isopropyl groups. The C *H*_2_ groups on the ethyl arms appear as a multiplet between 3.36–2.86 ppm. Three doublets at 1.51 ppm integrate to the 18 protons corresponding to the terminal methyl groups on the isopropyl arm while the terminal C *H*_3_ groups of the ethyl arms are seen as a multiplet between 1.17–0.91 ppm. The variable temperature ^1^H NMR of compound **6** (Figure 8) reveals similar results to those seen previously. However, at low temperature (−35 °C), much sharper peaks are observed, indicating a lower degree of fluxionality as compared to **2**. This is possibly because the bulkier ethyl substituents hinder the dynamics of the compound in solution. Three separate multiplets are seen for the isopropyl C *H* protons. The terminal methyl groups of the isopropyl arm are seen as 6 separate sharp doublets. The ethyl arms appear as three multiplets and three triplets, however, in this case there seems to be a lot of overlap between the peaks. Unlike the ^1^H NMR spectra for **2**, the analysis for **6** shows coalescence of peaks at a much lower temperature (60 °C) and by 90 °C a clear sharp spectrum is observed with one heptet, one quartet and a doublet and triplet, indicating that at this temperature, all ligands are in equivalent NMR environments. The hypothesis for this sort of behaviour in solution remains the same as for **2**: *in solutio* intramolecular rearrangement coupled with spatial proximity of the CH_3_ groups on the isopropyl arms. The fluxionality is again attributed to labile ligands undergoing the rupture and rotation mechanism, possibly resulting in a change in isomerism, which occurs slowly enough to observed at low temperature but too fast for the NMR timescale at higher temperatures. Again, no NH broad signal was observed indicating complete conversion of the pro‐ligands.

For signals at 3.9–4.5 ppm due to the NC(*H*)(CH_3_)_2_ protons, the coalescence temperature can be estimated to be 42±3 °C. Assuming that the observed exchange is between two sites with the population 2 : 1 and chemical shift difference of 286±15 Hz in the absence of the exchange, the free energies of activation can be estimated to be ΔGA≠=62.4±0.8 kJ mol^−1^ and ΔGB≠=60.6±0.8 kJ mol^−1^ using equations by Shanan‐Atidi and Bar‐Eli.^[40]^

### Thermal Gravimetric Analysis

To assess whether these compounds can act as suitable precursors that can break down into metallic aluminium, TGA was carried out for the compounds. The compounds were sealed in aluminium pans and heated from 30 °C–510 °C at a ramp rate of 10 °C min^−1^ under a constant flow of nitrogen. For the compounds of the type [Al(L^x^)_3_], it was expected that the three compounds undergoing *in solutio* intramolecular rearrangement would decompose at lower temperatures compared to the other compounds. It is presumed that fluxionality leads to lower decomposition temperatures as molecules are more likely to break apart during the decomposition process, making them more favourable and efficient as precursors.^[28]^ The bidentate N−C−N bound ligands might result in a slightly higher decomposition temperature due to the stronger bonds between aluminium and nitrogen. The bulkier phenyl compounds may also result in a higher carbon contamination in the deposited material. Derivates were also calculated for these curves to highlight the onset of thermal degradation of these compounds.

**Figure 9:**
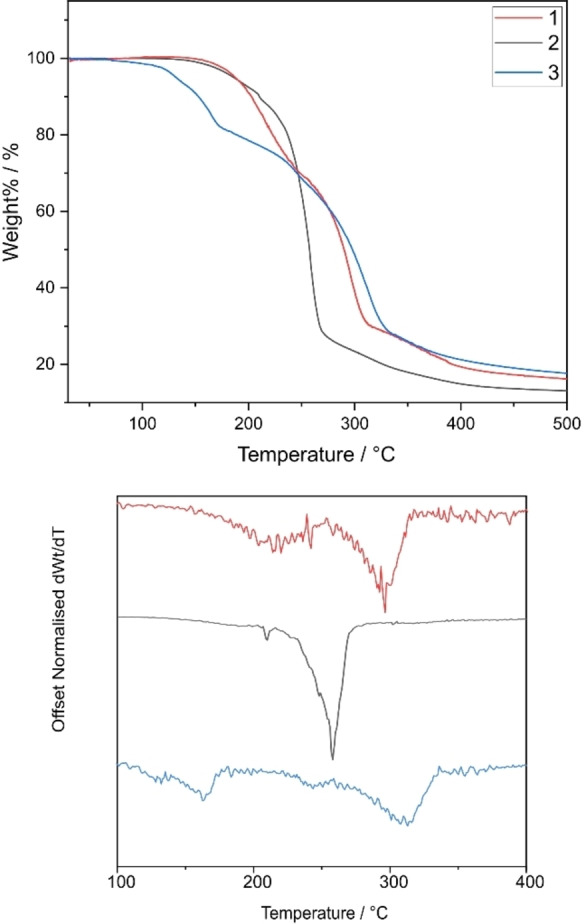
(top) Overlapping thermograms of compounds **1–3**, (bottom) Offset derivatives of thermograms with *dWt/dT*=0.

**Figure 10:**
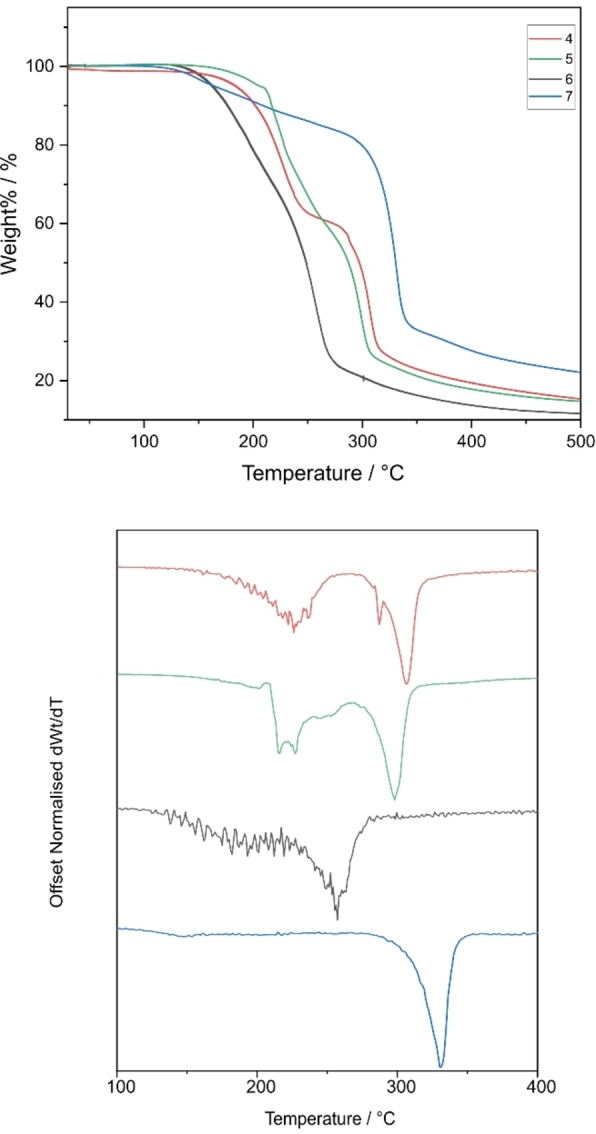
(top) Overlapping thermograms of compounds **4–7**, (bottom) Offset derivatives of thermograms with *dWt/dT*=0.

Thermal decompositions profiles were found to reflect what was expected from the crystallographic and NMR studies of the compounds. (Figures 9 and 10 show overlapping thermograms of compounds **1–7**). **2** and **6**, both of which include the presence of sterically bulky isopropyl groups, have elongated Al−N bond lengths and display fluxionality in the solution state were found to have the lowest decomposition temperatures of all seven compounds.

Both compounds degrade through a single event thermal event which is initiated at 163 °C for **2** and at 138 °C for **6** (taken from the point at which the data first deviates from dWt/dT=0) indicating that for complete conversion to aluminium metal, minimum sintering temperature of 163 and 138 °C would need to be reached for **2** and **6** respectively. However, the final weight percentages of the two compounds do not match the weight% of Al in the compound, indicating the presence of some impurities in the decomposed products (Table 6). **5** however, shows a two‐step decomposition. But as expected, its decomposition profile is lower than that of the other compounds that do not undergo fluxional behaviour. Conversely, **1**, **3**, **4** and **7** do not display single step decomposition events and their minimum sintering temperatures are higher than what is seen for the compounds with elongated Al−N bonds and labile metal centres. Compounds **1** and **4** have high onset of decomposition temperatures (the temperature at which 1 % mass loss has occurred) which may be a result of the lack of steric strain due to the less bulky methyl groups being able to sit comfortably close to the aluminium centre, while **3** and **7** show the presence of the highest level of carbon/sulfur contamination in the decomposed products. The 1st derivative curves of the TGA also show the presence of some mass loss occurring before the inflection point. These possibly correspond to small fragments of the ligand dissociating as also seen in the MSMS.

**Table 6:** Expected residual mass% for zerovalent aluminium, % non‐volatile residue and onset of decomposition/volatilization temperatures of **1–7**.

Aluminium Compound	Expected % for Al	% Non‐volatile residue (Temp)	Onset temperature
**1**	7.13	19.18 % (402 °C)	162.43 °C
**2**	5.83	15.66 % (385°C)	150.81 °C
**3**	4.78	21.10 % (402 °C)	84.48 °C
**4**	5.83	19.83 % (395 °C)	157.26 °C
**5**	5.35	16.79 % (424 °C)	172.59 °C
**6**	4.93	16.50 % (345 °C)	141.45 °C
**7**	4.16	27.37 % (402 °C)	129.19 °C

For the compounds of the type [RAl(L^x^)_2_] (**8**–**14**) where R=Me/Et, it is expected that the compounds that show greater distortion from ideal geometry would decompose at lower temperatures due to the misalignment of orbitals and the elongation of bonds. **8**, **9**, **10**, **11**, **12** and **14** have τ_5_ values of 0.02, 0.33, 0.40, 0.19, 0.31 and 0.24 respectively. As the ligand and compound could not be separated for compound **13**, TGA was not carried out for this as accurate decomposition profile of the compound could not be obtained.

**Figure 11:**
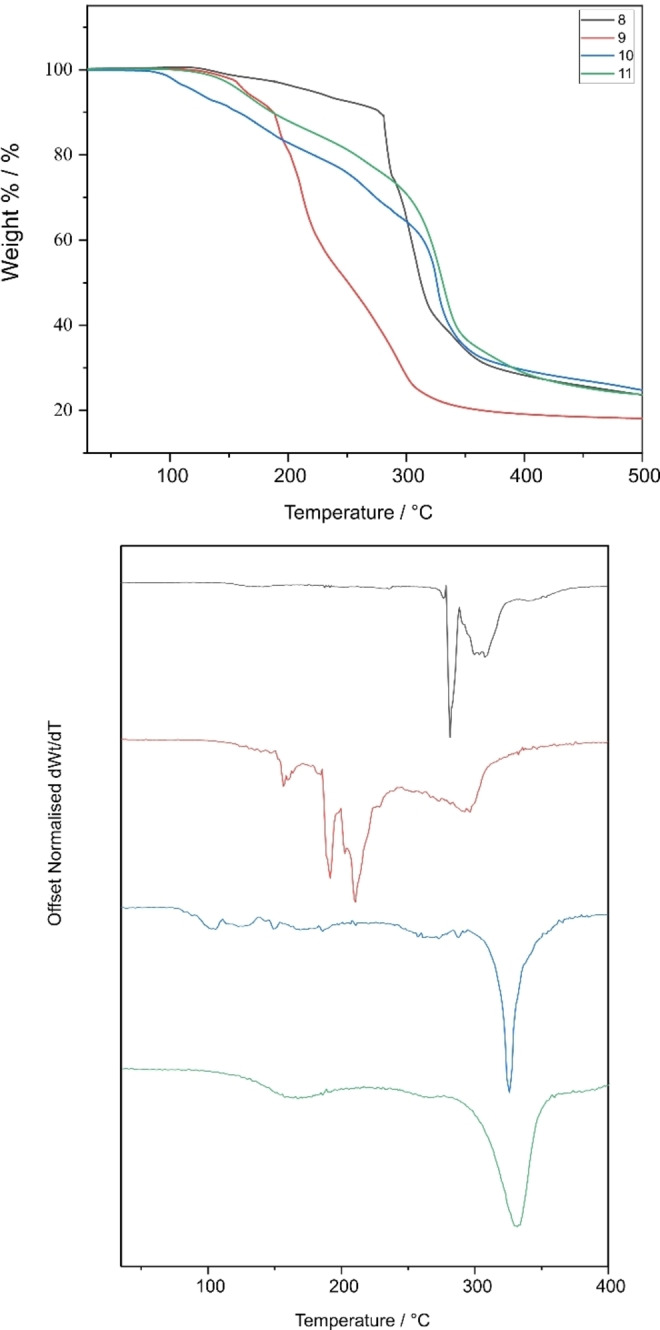
(top) Overlapping thermograms of compounds **8–11**, (bottom) Offset derivatives of thermograms with *dWt/dT*=0.

**Figure 12:**
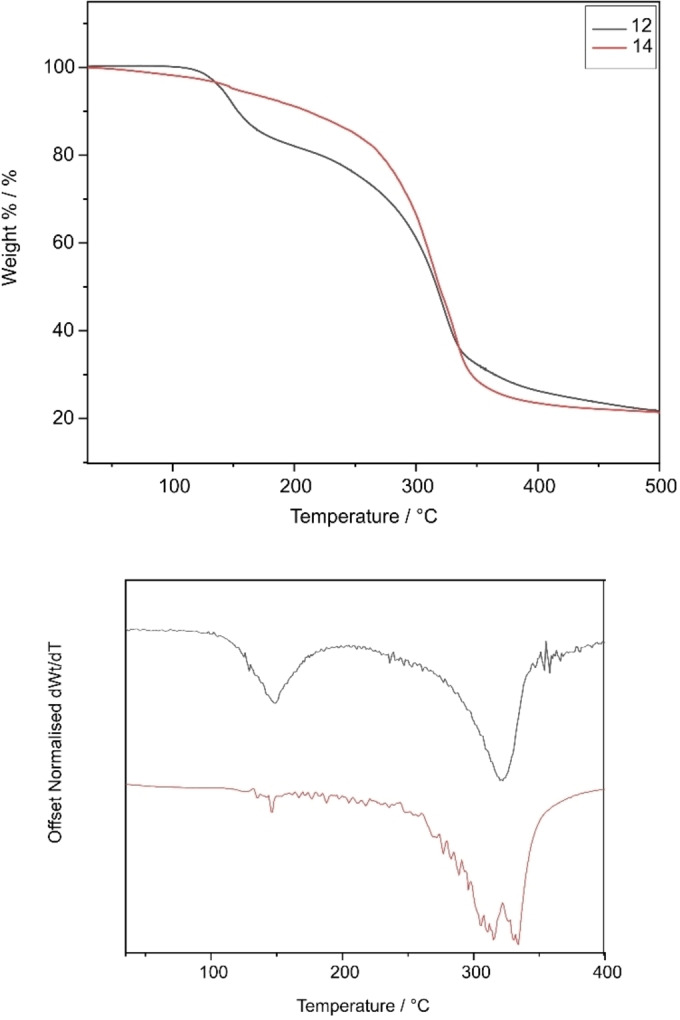
(top) Overlapping thermograms of compounds **12** and **14**, (bottom) Offset derivatives of thermograms with *dWt/dT*=0.

Figures 11 and 12 show the overlapping thermograms of these compounds. As expected, the compound with the least deviation from ideal geometry (**8**) shows the highest onset of decomposition temperature. The compounds with greater distortion show comparatively lower onset of decomposition temperatures though these are not in line with their deviation from ideal geometry. Unlike the tris(thioureide) compounds, almost all the bis(thioureide) alkyl aluminium(III) compounds show a two‐step decomposition with the first deviations from dWt/dT seen at 120, 107, 81, 106, 93 and 94 °C for **8**, **9**, **10**, **11**, **12**, and **14** respectively, which is in keeping with the deviation from ideal geometries for these compounds (Table 7). Unfortunately, TGA data alone do not indicate complete decomposition to elemental aluminium with the phenyl substituted compounds showing the most residue despite having lower onset of decomposition temperatures. There is evidence of mass loss occurring before the inflection point as seen in the 1st derivative curves. These might be arising as a result of ligand fragments dissociating at these temperatures as also seen in the MSMS.

**Table 7:** Expected residual mass% for zerovalent aluminium, % non‐volatile residue and onset of decomposition/volatilization temperatures of **8–12** and **14**.

Aluminium Compound	Expected % for Al	% Non‐volatile residue (Temp)	Onset temperature
**8**	9.76	29.3 % (385 °C)	147.16 °C
**9**	8.11	20.2 % (359 °C)	136.31 °C
**10**	6.74	30.5 % (384 °C)	93.49 °C
**11**	5.91	27.3 % (417 °C)	129.55 °C
**12**	6.51	27.0 % (390°C)	122.16 °C
**14**	5.73	23.8 % (394 °C)	73.02 °C

**Figure 13:**
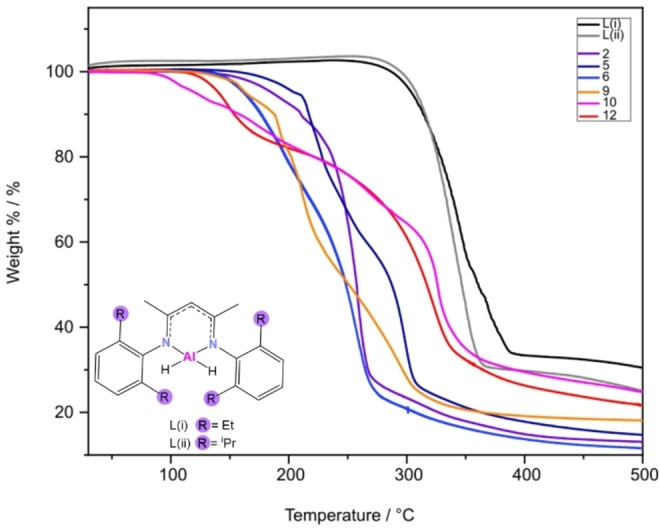
Overlapping thermograms of (a) Black and grey thermograms: literature compounds L(i) and L(ii) (shown in inset), (b) Solid lines in shades of blue and purple: tris(thioureide) aluminium(III) compounds (**2**, **5** and **7**) and (c): Solid lines in shades of red and orange: bis(thioureide) alkyl aluminium(III) compounds (**9**, **10** and **12**).

To gain a better understanding of how the presence of one hard and one soft base, as well as the presence of alkyl groups affects the decomposition temperatures of these compounds, comparisons were drawn between the TGA profiles of compounds **2**, **5**, **7**, **9**, **10** and **12**. Two β‐diketiminate aluminium hydride compounds were also synthesised using literature procedures. Following a crystal structure match to reported data,[^41^, ^42^] the thermal profiles of these literature compounds were also looked at to study how the decomposition profile for an aluminium(III) compound bound to two hard bases varies from those of the thioureide compounds. Figure 13 shows the thermal decomposition profiles for all these compounds while the inset image shows the structures of the two literature compounds. A stark difference is seen in the onset of decomposition temperatures of the two literature reported compounds and the various thioureide compounds reported here, reiterating the fact that replacing one hard base ligand atom with a softer one helps reduce the decomposition temperature without largely affecting the stability of these compounds. A comparison of the tris(thioureide) and bis(thioureide) alkyl aluminium(III) compounds reveals the tris(thioureide) aluminium(III) compounds to have a comparatively lower decomposition temperature which is possibly a result of the greater strain in the octahedral compounds as well as the hemi‐labile behaviour of the ligands in these compounds. However, the bis(thioureide) alkyl aluminium(III) compounds display almost the same or slightly higher decomposition temperatures in spite of having a strong Al−C bond. This is with the added benefit of a lower contamination due to the lack of one extra four‐membered chelate ring.

### Tandem Mass Spectromety

**Figure 14:**
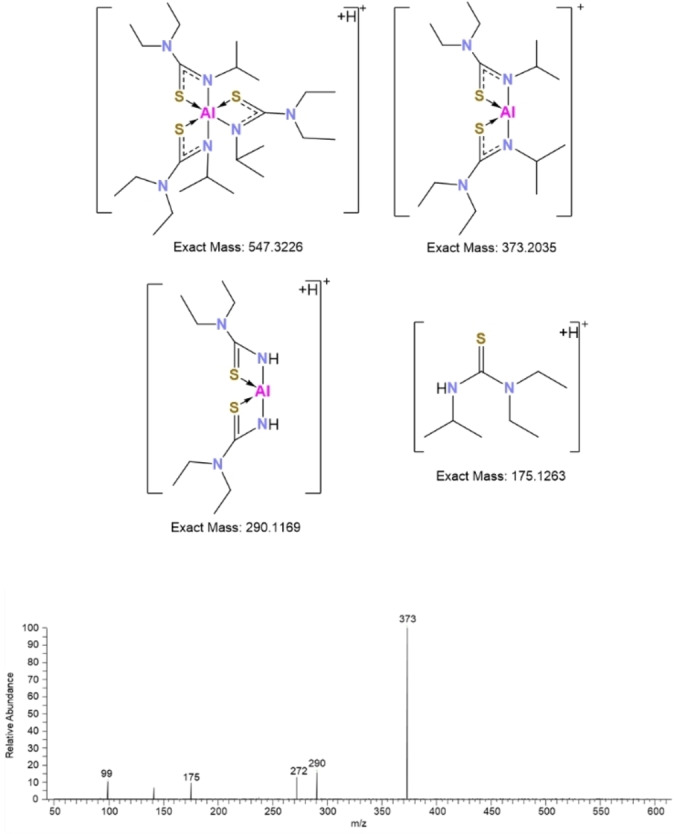
Top: Proposed mass fragmentation for compound **6**. Bottom: ASAP‐HESI MSMS spectrum on *m/z* 547.3226 in positive HESI mode acquired on the Exactive Q mass spectrometer at HCD 35 %.

To better understand the dissociation and breakdown of the novel aluminium compounds tandem mass spectrometry was carried out. The ions for compounds **1–12** were subject to different fragmentation energies and a full MSMS spectrum was acquired for each metal compound. The fragmentation patterns were similar for all compounds. An Exemplar pattern is shown in Figure 14. For **6**, the AlL_3_^+^H^+^ ion was seen at m/z=547.3326. MSMS for this generated a strong signal at m/z=373.2035 which corresponds to [AlL_3_‐L]^+^ ions, resulting from the loss of one ligand bound to the metal ion. The fragment at m/z=290.1169 corresponds to the loss of the isopropyl groups of the thioureide ligands bound to the metal ion while the signal at m/z=175.1263 corresponds to just the ligand with the elemental composition of C_8_H_18_N_2_S+H^+^. Other compounds also show similar fragmentation patterns, with fragments showing loss of one ligand, as well as fragments showing partial attachment of ligands to the aluminium ion. In certain cases, fragments with only one ligand bound to the metal ion are also observed, and finally, a clear signal for pure ligand is observed for all these compounds. This fragmentation is seen to take place between 120–180 °C. For some of the penta‐coordinate compounds fragmentation of the ligand ion into smaller molecules or ions is also observed. Contrary to expectations, the Al−C bond appears to fragment easily at <200 °C in some cases. These results indicate the potential for these molecules to act as precursors, since there seems to be evidence of the ligands cleanly dissociating.

## Conclusions

In this work, we have investigated the design and synthesis of aluminium compounds via systematic variation of ligand type. Their dynamic properties have also been investigated, with a focus on the decomposition profiles. This is the first example of a ligand directed design and study of aluminium compounds which decompose at low temperatures (<200 °C), opening up potential for structured design of precursors towards aluminium thin film deposition. Experimental findings are in excellent agreement with DFT calculations. DFT calculations confirmed that precursors with the smallest substituents on the nitrogen, showed a preferred N−C−N κ^2^ bonding. Undoubtedly showing for the first time, a clear correlation between the size of the substituents and preference for all three ligands to bind in a S−C−N fashion as the substituent size increases. It is evident that only the Me‐substituted ligands can bind through the harder N‐donor due to higher steric demands of bigger substituents in the tris‐ligated compounds.

A set of tris(thioureide) aluminium compounds (**1**‐**7**) have been synthesised with **2**, **5** and **6** observed to display fluxional behaviour in solution. Thorough VT ^1^H NMR investigations indicated a dynamic process in solution, which was hypothesised to be the labile behaviour of the three most spatially bulky ligands. This was firmly supported by TGA, which revealed that these fluxional compounds had a much lower onset of decomposition temperature as compared to the other four compounds also synthesised in this study. Compounds **8–14** are also reported, and TGA show that decomposition for these molecules is also below that of traditional aluminium β‐diketoiminates.

The results in this paper establish a clear correlation between fluxionality and decomposition temperature, which is a crucial step in developing precursors of aluminium that can decompose to elemental aluminium at low temperatures (<200 °C). These compounds were carefully designed to fulfil the requirement of being stable enough to be isolated and be easily handled for potential industrial use, but to be unstable enough to allow for a lower decomposition temperature. Achieving the fine balance between handleability/storage for real‐world application alongside the ability to act as useful precursors remains the ultimate challenge for precursor chemists. Case in point is the instability of reagents such as AlMe_3_ or aluminium hydrides, which are pyrophoric and therefore difficult to handle. The instability resulting from molecules that exhibit solution state fluxionality does not make these compounds highly reactive, and so molecules that exhibit dynamic behaviour could serve as safer alternatives to pyrophoric species in solution state deposition methods.

## Conflict of Interests

The authors declare no conflict of interest.

1

## Supporting information

As a service to our authors and readers, this journal provides supporting information supplied by the authors. Such materials are peer reviewed and may be re‐organized for online delivery, but are not copy‐edited or typeset. Technical support issues arising from supporting information (other than missing files) should be addressed to the authors.

Supporting Information

## Data Availability

The data that support the findings of this study are available from the corresponding author upon reasonable request.
