# Supplementary material for: Tuning Decomposition Temperature: A Structural Study of Ligand Directed Bonding and Fluxionality
Source: Chemistry. 2025 Mar 21;31(23):e202500178. doi: 10.1002/chem.202500178 (PMC12015401; doi:10.1002/chem.202500178)
Supplement: Supplementary file 1 — Supporting Information [file CHEM-31-e202500178-s001.pdf]

Chemistry–A European Journal

Supporting Information

Tuning Decomposition Temperature: A Structural Study of Ligand Directed Bonding and Fluxionality

Shreya Mrig, Petra Vasko, Salma Saeed, Abil E. Aliev, Kersti Karu, and Caroline E. Knapp*

Supporting Information

Tuning Decomposition Temperature: A Structural Study of Ligand Directed Bonding and Fluxionality

Shreya Mrig, Petra Vasko, Salma Saeed, Abil E. Aliev, Kersti Karu and Caroline E. Knapp*

1. Experimental
   a. General Procedures
   b. Ligand synthesis
   c. Synthesis of compounds **1** – **14**
   d. Synthesis of trimethylaminealane
2. Crystallographic data
   a. Crystallographic data for **2**
   b. Crystallographic data for **10**
3. NMR Spectra of compounds **1** – **14**
4. MSMS of compounds **1** – **12**
5. TGA Plots

1. Experimental

a. General Procedures

All organic reagents were procured from Sigma Aldrich unless stated otherwise. Isopropyl isothiocyanate was obtained from Alfa Aesar. Trimethylaluminium was procured from Epivalence Ltd. All non-deuterated solvents were pre-dried using dry solvent stills and were further dried over the appropriately sized molecular sieves (3 or 4 Å) or a potassium mirror. Deuterated solvents were obtained from Sigma Aldrich and were stored over 4 Å molecular sieves after drying using freeze-pump-thaw-cycles. Amines and isothiocyanates were used as supplied for ligand synthesis. All ligand preparations were performed using standard organic chemistry techniques. All reactions involving the formation or use of aluminium hydride or organoaluminium compounds were performed using standard Schlenk and glove-box techniques under an atmosphere of dried nitrogen. Recrystallisation was carried out either by storing the solutions in a 4 °C fridge, or in the glovebox at room temperature (RT) to allow slower growth. All thioureide aluminium compounds were stored in the glovebox at RT..

Nuclear Magnetic Resonance (NMR): All data was recorded in $CDCl_3/C_6D_6/C_6D_5\text{-}CD_3$ using Bruker Avance 300 MHz, Avance III 400 MHz, Avance Neo 500 or Avance III 600 MHz instruments. ^1H and $^{13}C\{^1H\}$ chemical shifts are reported relative to residual solvent peaks. ^1H and $^{13}C\{^1H\}$ chemical shifts were routinely confirmed by ^1H-^1H (COSY) and ^1H-^{13}C (HSQC) experiments where necessary.

SCXRD: Suitable crystals were selected and mounted on a nylon loop on a SuperNova Atlas (Dual) diffractometer using Cu K_α radiation (λ = 1.54184 Å) Crystals were kept at 150.00(10) K during data collection. Structures were solved by various methods. For compounds **1**, **2**, **3**, **6**, **7**, **9**, **10**, **12** and **14**: using Olex2[1], the structure was solved with the ShelXT[2] structure solution program using Intrinsic Phasing and refined with the ShelXL[3] refinement package using Least Squares minimisation. For compound **5**: using Olex2, the structure was solved with the olex2.solve[4] structure solution program using Charge Flipping and refined with the ShelXL refinement package using Least Squares minimisation. For compounds **8** and **13** using Olex2, the structure was solved with the ShelXT structure solution program using Intrinsic Phasing and refined with the olex2.refine refinement package using Gauss-Newton minimisation.

Deposition Number(s) <url href="https://www.ccdc.cam.ac.uk/services/structures?id=doi:10.1002/chem.202500178">2257998 (**1**), 2257999 (**2**), 2258000 (**3**), 2258001 (**5**), 2258002 (**6**), 2258003 (**7**), 2258018 (**8**), 2258021 (**9**), 2258019 (**10**), 2258020 (**11**), 2258022 (**12**), 2258023 (**13**), 2258024 (**14**)</url> contain(s) the supplementary crystallographic data for this paper. These data are provided free of charge by the joint Cambridge Crystallographic Data Centre and Fachinformationszentrum Karlsruhe <url href="http://www.ccdc.cam.ac.uk/structures">Access Structures service</url>.

Mass Spectrometry (MS): Data was collected using an ASAP-HESI source. A glass capillary was used to hold 50 µg portion of a solid sample which was them mounted into an ASAP probe (Advion, UK) and inserted into a HESI source. The ASAP-HESI ion source was connected to a Q Exactive Plus mass spectrometer (Thermo Fischer Scientific, UK). Auxiliary gas was set to 5, the sheath and sweep gases were set to 0. The capillary temperature was 200 °C and the auxiliary gas heater temperature was varied between 80 – 200 °C for different compounds. Nitrogen was used as the auxiliary gas. The discharge needle was installed, and voltage set to 4.5 kV. The Q Exactive mass spectrometer was operated in a positive ion mode, and at 140,000 resolution at *m/z* 400 (full width at half maximum height) with the mass range set to *m/z* 100 – 1000. The Q Exactive mass spectrometer readings were as follows: capillary voltage −0.8, bent flatpole 5.9, injection flatpole 7.7, quadrupole exit DC −11, C-trap entrance lens 11, C-trap RF amp 1527, C-trap RF frequency 3.2, C-trap exit lens −60 and HCD exit lens 34. The instrument was calibrated using the manufactures “tune mixture” consisting of caffeine, MRA and Ultramark 1621 for positive mode. Each sample had an analysis time of 60 s and all spectra were acquired in profile mode. Tandem mass spectra (MSMS) were acquired with high energy collisional dissociation (HCD). The spectra were produced at different collision energies: 10, 15, 25, 35 and 50% with the MSMS scan type set for the isolation of precursor ion with 1 *m/z* width. Data were analyzed using a FreeStyle Xcalibur software (Thermo Fischer).

Elemental Analysis (EA): Analysis for compounds **1** – **7** was carried out *via* the Dumas combustion method at Elemental Microanalysis Ltd. EA for the remaining compounds was carried out at London Metropolitan University using a Carlo Erba CE1108 elemental analyser.

TGA: Measurements for all compounds barring **13** and **14** were carried out on a PerkinElmer STA6000 TGA instrument. Samples were sealed in a glovebox and heated from 30 °C to 510 °C at a heating rate of 10 °C min^{-1} under a shield flow of nitrogen. Sample crucibles were pierced just before measurement to allow for mass loss. Data for **14** was obtained from a Discovery TGA 5500 instrument, collected using the Trios software with the same measurement criteria. TGA data for **13** could not be obtained.

b. Ligand Synthesis

Synthesis of 1,1,3-trimethylthiourea (L^1H)

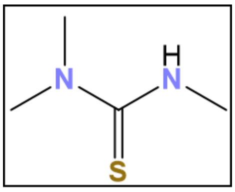

The synthesis was carried out with appropriate modifications to the procedure described by Sullivan *et al.*[5] To a flask containing dimethylamine, 2 M in THF, (30 mL, 60 mmol), methyl isothiocyanate (4.38 g, 60 mmol) was added slowly. The reaction mixture was allowed to stir at RT for 1 h. Following this, the reaction mixture was reduced under vacuum to result in the formation of a white powder of **L^1H**. The product was collected by filtration and washed with hexane.

^{1}H NMR δ/ppm (300 MHz, C_6D_6): 4.42 (1H, *s* (br), N*H*), 2.89 (3H, d, *J* = 4.4 Hz, N(H)CH_3), 2.53 (6H, *s*, N(CH_3)$_2$).

Synthesis of 3-ethyl-1,1-dimethylthiourea (L^2H)

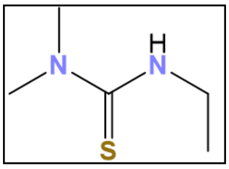

Dimethylamine, 2 M in THF, (30 mL, 60 mmol) and ethyl isothiocyanate (5.25 mL, 60 mmol) were combined and allowed to stir at RT for 1 h. The reaction mixture was reduced under vacuum to yield a viscous yellow oil of **L^2H**.

^{1}H NMR δ/ppm (500 MHz, C_6D_6): 5.59 (1H, *s* (br), N*H*), 3.84 – 3.54 (2H, m, N(H)CH_2CH$_3$), 2.78 (6H, *s*, N(CH_3)$_2$), 1.10 (3H, t, *J* = 7.1 Hz, N(H)CH$_2$CH_3).

Synthesis of 3-isopropyl-1,1-dimethylthiourea (L^3H)

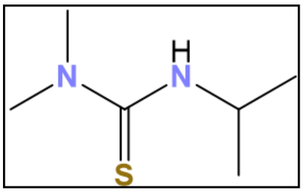

Isopropyl isothiocyanate (6.40 mL, 60 mmol) was slowly added to a flask containing dimethylamine, 2 M in THF, (30 mL, 60 mmol) and hexane (10 mL). The reaction mixture was allowed to stir for 1 h and then reduced under vacuum. A white powder was formed which was filtered and washed with hexane to yield $\mathbf{L^3H}$ as bright white crystals.

^{1}**H NMR** δ/ppm (300 MHz, C_6D_6): 4.86 (1H, m, $N(H)C(\underline{\mathit{H}})(CH_3)_2$), 4.55 (1H, *s* (br), $N\underline{\mathit{H}}$), 2.58 (6H, *s*, $N(C\underline{\mathit{H}}_3)_2$), 1.05 (6H, d, *J* = 6.5 Hz, $C(H)(C\underline{\mathit{H}}_3)_2$).

Synthesis of 1,1-dimethyl-3-phenylthiourea (*L^4H*)

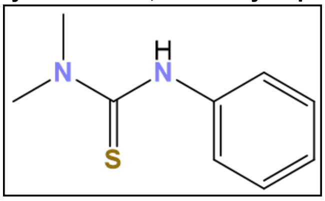

A solution of phenyl isothiocyanate (7.17 mL, 60 mmol) and THF (10 mL) was added to dimethylamine, 2 M in THF, (30 mL, 60 mmol). The reaction mixture was allowed to stir for 1 h in which time a white precipitate had formed. The precipitate was filtered, washed with hexane and dried to yield white crystals of $\mathbf{L^4H}$.

^{1}**H NMR** δ/ppm (300 MHz, C_6D_6): 7.15 – 6.84 (5H, m, Ph-$\underline{\mathit{H}}$), 6.47 (1H, *s* (br), $N\underline{\mathit{H}}$), 2.54 (6H, *s*, $N(C\underline{\mathit{H}}_3)_2$).

Synthesis of 1,1-diethyl-3-methylthiourea (*L^5H*)

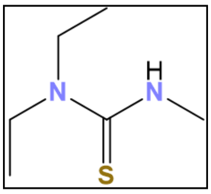

Diethylamine (6.18 mL, 60 mmol) and methyl isothiocyanate (4.38 g, 60 mmol) were combined together and allowed to stir for 1 h. The reaction mixture was then reduced under vacuum to yield a viscous yellow-orange liquid of $\mathbf{L^5H}$.

^{1}**H NMR** δ/ppm (300 MHz, C_6D_6): 5.82 (1H, *s* (br), $N\underline{\mathit{H}}$), 3.34 (4H, q, *J* = 7.1 Hz, $N(C\underline{\mathit{H}}_2CH_3)_2$), 3.06 (3H, d, *J* = 4.4 Hz, $N(H)C\underline{\mathit{H}}_3$), 0.93 (6H, t, *J* = 7.1 Hz, $N(CH_2C\underline{\mathit{H}}_3)_2$).

Synthesis of 1,1,3-triethylthiourea (L^6H)

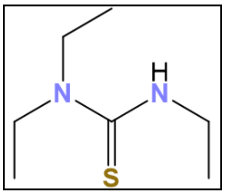

Ethyl isothiocyanate (5.25 mL, 60 mmol) was slowly added to a flask containing diethylamine (6.18 mL, 60 mmol). The reaction mixture was allowed to stir for 1 h and reduced under vacuum to yield a dark yellow-orange viscous oil of $\mathbf{L^6H}$.

^{1}**H NMR** δ/ppm (500 MHz, C_6D_6): 5.26 (1H, *s* (br), $N\underline{\mathit{H}}$), 3.74 – 3.65 (2H, m, $N(H)C\underline{\mathit{H}}_2CH_3$), 3.28 (4H, q, *J* = 7.2 Hz, $N(C\underline{\mathit{H}}_2CH_3)_2$), 1.06 (3H, t, *J* = 6.9 Hz, $N(H)CH_2C\underline{\mathit{H}}_3$), 0.90 (6H, t, *J* = 6.9 Hz, $N(CH_2C\underline{\mathit{H}}_3)_2$).

Synthesis of 1,1-diethyl-3-isopropylthiourea (*L^7H*)

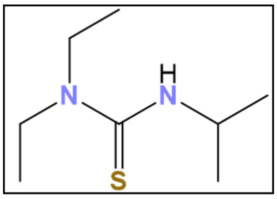

Isopropyl isothiocyanate (6.40 mL, 60 mmol) and diethylamine (6.18 mL, 60 mmol) were combined together and allowed to stir at RT. In 15 min a peach-white precipitate had formed which was filtered, washed with hexane and dried to yield a peach-white powder of $\mathbf{L^7H}$.

^{1}**H NMR** δ/ppm (500 MHz, C_6D_6): 4.92 (1H, m, $N(H)C(\underline{\mathit{H}})(CH_3)_2$), 4.77 (1H, *s* (br), $N\underline{\mathit{H}}$), 3.22 (4H, q, *J* = 7.1 Hz, $N(C\underline{\mathit{H}}_2CH_3)_2$), 1.07 (6H, d, *J* = 6.5 Hz, $N(H)C(H)(C\underline{\mathit{H}}_3)_2$), 0.87 (6H, t, *J* = 7.1 Hz, $N(CH_2C\underline{\mathit{H}}_3)_2$).

Synthesis of 1,1-diethyl-3-phenylthiourea (*L^8H*)

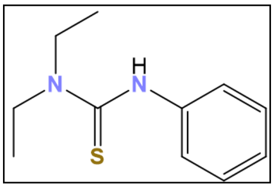

Solutions of diethylamine (6.18 mL, 60 mmol) in THF (20 mL) and phenyl isothiocyanate (7.17 mL, 60 mmol) in THF (30 mL) were combined and refluxed for 2 h. Following this, the reaction mixture was reduced *in vacuo* to yield a yellow-orange viscous oil of $\mathbf{L^8H}$.

^{1}**H NMR** δ/ppm (500 MHz, C_6D_6): 7.36 (2H, d, *J* = 7.3 Hz, Ph-$\underline{\mathit{H}}$), 7.18 – 7.09 (2H, m, Ph-$\underline{\mathit{H}}$), 6.96 (1H, t, *J* = 7.5 Hz, Ph-$\underline{\mathit{H}}$), 6.90 (1H, *s* (br), $N\underline{\mathit{H}}$), 3.25 (4H, q, *J* = 7.1 Hz, $N(C\underline{\mathit{H}}_2CH_3)_2$), 0.87 (6H, t, *J* = 7.1 Hz, $N(CH_2C\underline{\mathit{H}}_3)_2$).

c. **Synthesis of compounds 1 – 14.**

Synthesis of [Al(L^1)$_3$] (*1*)

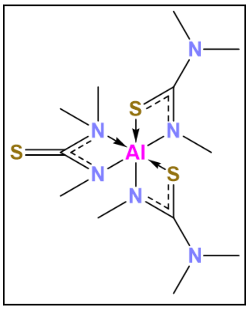

A solution of **L^1H** (0.39 g, 3.36 mmol) in toluene (5 mL) was added to a cooled solution of TMAA (0.10 g, 1.12 mmol) in toluene (5 mL). The reaction mixture was allowed to warm to RT and left to stir overnight. Filtration and removal of solvent *in vacuo* afforded a solid. Recrystallisation from a hot toluene-hexane solution resulted in clear crystals of **1**. Yield of single crystals: 52.1%

^{1}H NMR δ/ppm (500 MHz, C_6D_6): 3.21 (3H, *s*, NC(S)NC$\underline{H}_3$), 2.98 – 2.10 (24H. m, NCNC$\underline{H}_3$ and N(C$\underline{H}_3$)$_2$). **^{13}C{^{1}H} NMR** δ/ppm (126 MHz, C_6D_6): 42.01 (N($\underline{C}$H$_3$)$_2$ and NCN($\underline{C}$H$_3$)$_2$), 35.43 (N($\underline{C}$H$_3$)$_2$ and NCN$\underline{C}$H$_3$), 34.33 (NC(S)N$\underline{C}$H$_3$). **EA** calc.% C: 38.07, H: 7.19, N: 22.20, found%: C: 39.39, H: 7.14, N: 21.99. **MS**: *m/z* 379.1347 [Al-(L^1)$_3$+H]$^+$.

Synthesis of [Al(L^3)$_3$] (*2*)

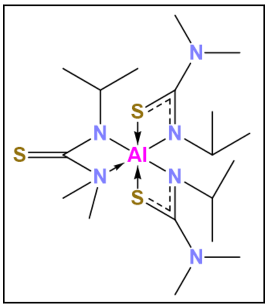

A solution of **L^3H** (0.98 g, 6.73 mmol) in toluene (5 mL) was slowly added to a cooled solution of TMAA (0.20 g, 2.24 mmol) in toluene (10 mL), allowed to warm to RT and stirred overnight. Filtration and removal of solvent *in vacou* resulted in a solid product. Recrystallisation from toluene yielded colourless crystals of **2**. Yield of single crystals: 44.5%

^{1}H NMR δ/ppm (500 MHz, C_6D_6): 4.87 – 4.78 (1H, m, NC($\underline{H}$)(CH$_3$)$_2$), 3.86 (1H, p, *J* = 6.7 Hz, NC($\underline{H}$)(CH$_3$)$_2$), 3.70 (1H, p, *J* = 6.7 Hz, NC($\underline{H}$)(CH$_3$)$_2$), 2.98 – 2.30 (18H, m, N(C$\underline{H}_3$)$_2$), 1.68 – 0.97 (18H, m, NC(H)(C$\underline{H}_3$)$_2$). **^{13}C{^{1}H} NMR** δ/ppm (126 MHz, C_6D_6): 50.92 (N$\underline{C}$(H)(CH$_3$)$_2$), 49.85 (N$\underline{C}$(H)(CH$_3$)$_2$), 49.63 (N$\underline{C}$(H)(CH$_3$)$_2$), 47.35 – 39.05 (m) (N($\underline{C}$H$_3$)$_2$), 23.87 – 19.79 (m) (NC(H)($\underline{C}$H$_3$)$_2$). **EA** calc.% C: 46.72, H: 8.50, N: 18.16, found%: C: 47.50, H: 8.65, N: 18.35. **MS**: *m/z* 463.2286 [Al-(L^3)$_3$+H]$^+$.

Synthesis of [Al(L^4)$_3$] (*3*)

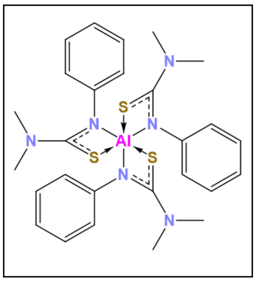

A solution of **L^4H** (1.21 g, 6.73 mmol) in toluene (10 mL) was added to a cooled solution of TMAA (0.20 g, 2.24 mmol) in toluene (10 mL). The reaction mixture was allowed to warm up to RT and left to stir overnight. The resultant clear solution was filtered and reduced *in vacuo* to yield a solid which was recrystallised from warm toluene to afford colourless crystals of **3**. Yield of single crystals: 65.3%

^{1}H NMR δ/ppm (500 MHz, C_6D_6): 7.14 – 6.01 (15H, m, Ph-$\underline{H}$), 2.34 (18H, *s*, N(C$\underline{H}_3$)$_2$). **^{13}C{^{1}H} NMR** δ/ppm (126 MHz, C_6D_6): 126.51 – 123.44 (m) (Ph-$\underline{C}$), 42.34 (N($\underline{C}$H$_3$)$_2$). **EA** calc.% C: 57.42, H: 5.89, N: 14.88, found: C: 55.60, H: 6.09, N: 14.26. **MS**: *m/z* 565.1817 [Al-(L^4)$_3$-H]$^+$

Synthesis of [Al(L^5)$_3$] (*4*)

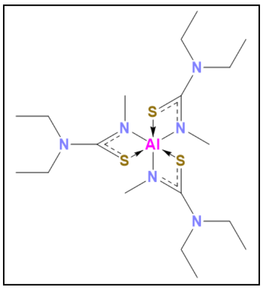

A solution of **L^5H** (4.91 g, 33.66 mmol) in toluene (20 mL) was added to a cooled solution of TMAA (1.20 g, 13.46 mmol) in toluene (25 mL). The reaction mixture was allowed to warm up to RT and then heated to 115 °C. The reaction mixture was allowed to reflux overnight to result in a dark brown clear solution. This was filtered and the solution removed *in vacuo* to yield a white solid. The powder was recrystallised from warm toluene and the resulting crystals washed with cold toluene (3 x 15 mL) to afford clear colourless crystals of **4**. Yield of single crystals: 78.2%

^{1}H NMR δ/ppm (500 MHz, Tol): 3.17 (12H, q, *J* = 7.1 Hz, $N(C\underline{H}_2CH_3)_2$), 2.97 (9H, *s*, $N(C\underline{H}_3)$), 0.98 (18H, t, *J* = 6.9 Hz, $N(CH_2C\underline{H}_3)_2$). **^{13}C{^{1}H} NMR** δ/ppm (126 MHz, Tol) 46.73 ($N(\underline{C}H_2CH_3)_2$), 14.29 ($N(CH_2\underline{C}H_3)_2$). **EA** calc.% C: 46.72, H: 8.50, N: 18.16, found%: C: 47.08, H: 8.58, N: 18.15.**MS**: *m/z* 463.2286 $[Al\text{-}(L^5)_3+H]^+$

Synthesis of [Al(L^6)$_3$] (5)

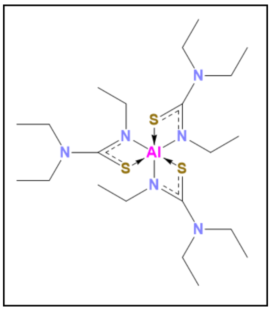

A solution of **L^6H** (3.77 g, 23.56 mmol) in toluene (15 mL) was slowly added to a cooled solution of TMAA (0.9 g, 10.09 mmol) in toluene (15 mL) and allowed to warm up to RT. The reaction mixture was left to reflux for 48 h to result in a dark brown solution. This solution was allowed to cool to RT, filtered, reduced under vacuum and stored at 4 °C for 2 days to produce a solid product. The solid product was recrystallised from warm toluene and washed with toluene (6 x 15 mL) to afford clear crystals of **5**. Yield of single crystals: 22.1%

^{1}H NMR δ/ppm (500 MHz, C_6D_6): 3.54 (6H, *s* (br), $N(C\underline{H}_2CH_3)$), 3.33 – 2.92 (12H, m, $N(C\underline{H}_2CH_3)_2$), 1.28 (9H, t, *J* = 7.1 Hz, $N(CH_2C\underline{H}_3)$), 0.99 (18H, t, *J* = 7.0 Hz, $N(CH_2C\underline{H}_3)_2$). **^{13}C{^{1}H} NMR** δ/ppm (126 MHz, C_6D_6): 47.16 ($N(\underline{C}H_2CH_3)_2$), 42.77 ($N(\underline{C}H_2CH_3)$), 16.25 ($N(CH_2\underline{C}H_3)_2$), 14.05 ($N(CH_2\underline{C}H_3)$. **EA** calc.% C: 49.97, H: 8.99, N: 16.65, found%: C: 47.25, H: 8.53, N: 15.23. **MS**: *m/z* 505.2756 $[Al\text{-}(L^6)_3+H]^+$

Synthesis of [Al(L^7)$_3$] (*6*)

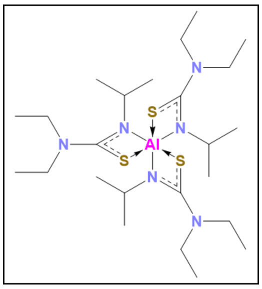

A solution of **L^7H** (2.98 g, 17.17 mmol) in toluene (15 mL) was added to a cooled solution of TMAA (0.51 g, 5.72 mmol) in toluene (20 mL), allowed to warm to RT and stirred overnight. Filtration and removal of solvent under vacuum resulted in a solid product. Recrystallisation from toluene afforded clear colourless crystals of **6**. Yield of single crystals: 51.8%

^{1}H NMR δ/ppm (600 MHz, C_6D_6): 4.40 (1H, *s* (br) $NC(\underline{H})(CH_3)_2$), 4.12 – 3.80 (2H, m, $NC(\underline{H})(CH_3)_2$), 3.36 – 2.86 (12H, m, $N(C\underline{H}_2CH_3)_2$), 1.51 (18H, ddd, *J* = 96.3, 58.8, 15.5 Hz, $NC(H)(C\underline{H}_3)_2$), 1.17 – 0.91 (18H, m, $N(CH_2C\underline{H}_3)_2$). **^{13}C{^{1}H} NMR** δ/ppm (126 MHz, C_6D_6): 52.20 – 47.74 (m) ($N\underline{C}(H)(CH_3)_2$), 46.86 ($N(\underline{C}H_2CH_3)_2$), 23.51 – 21.55 (m) ($NC(H)(\underline{C}H_3)_2$), 14.11 – 13.87 ($N(CH_2\underline{C}H_3)_2$). **EA** calc.%: C: 52.71, H: 9.40, N: 15.37, found%: C: 53.17, H: 9.07, N: 15.52. **MS**: *m/z* 547.3226 $[Al\text{-}(L^7)_3+H]^+$.

Synthesis of [Al(L^8)$_3$] (*7*)

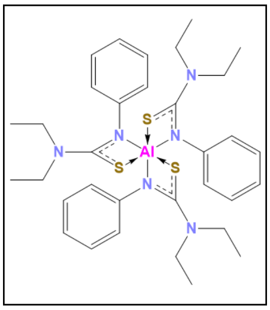

A solution of **L^8H** (1.75 g, 8.41 mmol) in toluene (10 mL) was added to a cooled solution of TMAA (0.25 g, 2.80 mmol) in toluene (15 mL). The reaction mixture was allowed to warm up to RT and stirred overnight. Filtration and *in vacou* reduction of the resultant solution yielded a solid product. Recrystallisation from warm toluene afforded clear crystals of **7**. Yield of single crystals: 35.7%

^{1}H NMR δ/ppm (500 MHz, C_6D_6): 7.57 – 7.18 (3H, m, Ph-$\underline{H}$), 7.11 (6H, t, *J* = 7.6 Hz, Ph-$\underline{H}$), 6.95 (6H, t, *J* = 7.4 Hz, Ph-$\underline{H}$), 3.07 – 2.87 (12H, m, $N(C\underline{H}_2CH_3)_2$), 0.67 (18H, t, *J* = 7.0 Hz, $N(CH_2C\underline{H}_3)_2$). **^{13}C{^{1}H} NMR** δ/ppm (126 MHz, C_6D_6): 127.25 – 122.89 (m, Ph-$\underline{C}$), 46.58 ($N(\underline{C}H_2CH_3)_2$), 13.13 ($N(CH_2\underline{C}H_3)_2$). **EA** calc.%: C: 61.08, H: 6.99, N: 12.95, found%: C: 61.22, H: 7.15, N: 13.07. **MS**: *m/z* 649.2756 $[Al\text{-}(L^8)_3+H]^+$

Synthesis of [Al(L^1)$_2$Me] (*8*)

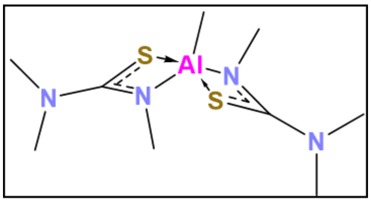

A solution of **L^1H** (0.50 g, 4.24 mmol) in toluene (15 mL) was added to a cooled solution of trimethylaluminium (0.15 g, 2.12 mmol) in toluene (10 mL), allowed to warm up to RT and stirred overnight following which an evolution of gas was observed. A pale-yellow solution was obtained which was filtered and dried *in vacuo* to yield a solid product. Recrystallisation from toluene afforded clear crystals of **8**. Yield: 72.81%

^{1}H NMR δ/ppm (500 MHz, C_6D_6): 2.75 (6H, *s*, N(C$\underline{H}_3$)), 2.48 (12H, *s*, N(C$\underline{H}_3$)$_2$) 0.29 (3H, *s*, Al(C$\underline{H}_3$). **^{13}C{^{1}H} NMR** δ/ppm (126 MHz, C_6D_6): 41.99 (N($\underline{C}$H$_3$)$_2$, 35.81 (N($\underline{C}$H$_3$)). **EA** calc.% C: 39.11, H: 7.66, N: 20.27, found%: C: 38.80, H: 7.82, N: 19.55. **MS**: *m/z* 277.1092 [Al(Me)-(L^1)$_2$+H]$^+$

Synthesis of [Al(L^5)$_2$Me] (*9*)

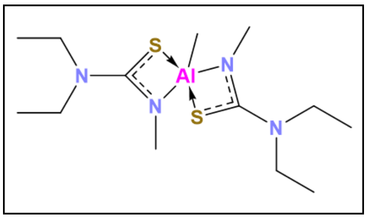

A solution of **L^5H** (0.81 g, 5.55 mmol) in toluene (15 mL) was slowly added to a cooled solution of trimethylaluminium (0.20 g, 2.77 mmol) in toluene (10 mL), allowed to warm up to RT and stirred overnight following which an evolution of gas was observed. The reaction mixture was filtered to obtain a clear yellow solution which was reduced under vacuum to afford a solid product. Recrystallisation from toluene yielded clear crystals of **9**. Yield: 71.74%

^{1}H NMR δ/ppm (500 MHz, C_6D_6): 3.06 – 3.01 (8H, m, N(C$\underline{H}_2$CH$_3$)$_2$), 2.81 (6H, *s*, N(C$\underline{H}_3$)), 0.82 (12H, t, J = 7.0 Hz, N(CH$_2$C$\underline{H}_3$)$_2$), 0.30 (3H, *s*, Al(C$\underline{H}_3$)). **^{13}C{^{1}H} NMR** δ/ppm (126 MHz, C_6D_6): 46.24 (N($\underline{C}$H$_2$CH$_3$)$_2$), 35.45 (N($\underline{C}$H$_3$)), 13.80 (N(CH$_2\underline{C}$H$_3$)$_2$). **EA** calc.%: C: 46.96, H: 8.79, N: 16.85, found%: C: 47.00, H: 8.72, N: 15.70. **MS**: *m/z* 333.1722 [Al(Me)-(L^5)$_2$+H]$^+$

Synthesis of [Al(L^4)$_2$Me] (*10*)

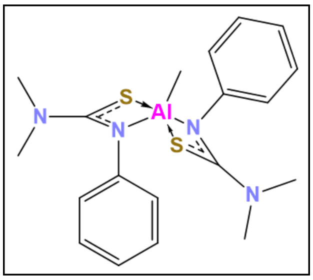

A solution of **L^4H** (0.99 g, 5.54 mmol) in toluene (10 mL) was slowly added to a cooled solution of trimethylaluminium (0.20 g, 2.77 mmol) in toluene (5 mL), allowed to warm to RT and stirred overnight following which an evolution of gas was observed. This was filtered to give a clear colourless solution which was reduced *in vacuo* to yield a solid. This was recrystallised from warm toluene to afford clear crystals of **10**. Yield 55.45%

^{1}H NMR δ/ppm (500 MHz, C_6D_6): 7.06 – 6.81 (10H, m, Ph-$\underline{H}$) 2.28 (12H, *s*, N(C$\underline{H}_3$)$_2$), 0.40 (3H, *s*, Al(C$\underline{H}_3$)). **^{13}C{^{1}H} NMR** δ/ppm (126 MHz, C_6D_6): 129.33 – 128.51 (Ph-$\underline{C}$), 125.70 – 123.91 (Ph-$\underline{C}$), 42.36 (N($\underline{C}$H$_3$)$_2$). **EA** calc.%: C: 56.98, H: 6.29, N: 13.99, found%: C: 60.01, H: 6.49, N: 11.68. **MS**: *m/z* 401.1403 [Al(Me)-(L^4)$_2$+H]$^+$

Synthesis of [Al(L^8)$_2$Me] (*11*)

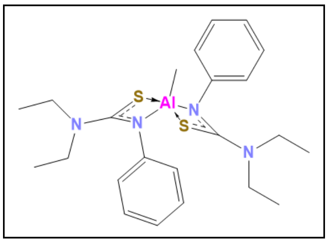

A solution of **L^8H** (1.15 g, 5.54 mmol) in toluene (10 mL) was slowly added to a cooled solution of trimethylaluminium (0.20 g, 2.77 mmol) in toluene (5 mL). The reaction mixture was allowed to warm to RT and stirred overnight following which an evolution of gas was observed. The resultant solution was filtered, reduced under vacuum and stored at 4 °C to afford a crude solid product. Recrystallisation from toluene yielded clear crystals of **11**. Yield: 52.35%

^{1}H NMR δ/ppm (500 MHz, C_6D_6): 7.15 – 6.82 (10H, m, Ph-$\underline{H}$) 3.04 – 2.80 (8H, m, N(C$\underline{H}_2$CH$_3$)$_2$), 0.67 (12H, t, J = 7.1 Hz, N(CH$_2$C$\underline{H}_3$)$_2$), 0.33 (3H, *s*, Al(C$\underline{H}_3$)). **^{13}C{^{1}H} NMR** δ/ppm (126 MHz, C_6D_6): 129.12 (Ph-$\underline{C}$), 124.77 (Ph-$\underline{C}$),

124.33(Ph-$\underline{C}$), 46.60 (N($\underline{C}H_2CH_3)_2$), 12.86 (N($CH_2\underline{C}H_3)_2$). **EA** calc.% C: 60.50, H: 7.28, N: 12.27, found%: C: 60.53, H: 7.30, N: 11.72. **MS**: *m/z* 457.2032 [Al(Me)-$(L^8)_2$+H]$^+$

Synthesis of [Al(L^4)$_2$Et] (*12*)

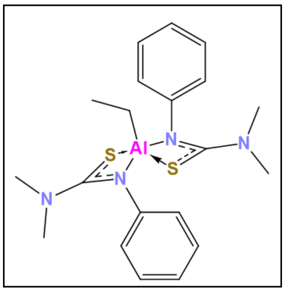

A solution of **L^4H** (1.18 g, 6.57 mmol) in toluene (20 mL) was slowly added to a cooled solution of triethylaluminium (0.50 g, 4.37 mmol) in toluene (15 mL), allowed to warm to RT and stirred overnight. The reaction mixture was filtered, and solvent removed *in vacuo* to yield a solid product. Recrystallisation from a warm toluene:hexane 1:1 solution yielded crystals of **12**. Yield of single crystals: 15.27%
^{1}H NMR δ/ppm (500 MHz, C_6D_6): 7.14 – 6.76 (10H, m, Ph-$\underline{H}$), 2.30 (12H, *s*, N($C\underline{H}_3)_2$), 1.77 (3H, t, *J* = 8.1 Hz, Al($CH_2C\underline{H}_3$)), 1.02 (2H, q, *J* = 8.1 Hz, Al($C\underline{H}_2CH_3$)). **^{13}C{^{1}H} NMR** δ/ppm (126 MHz, C_6D_6): 124.84 – 123.87 (m, Ph-$\underline{C}$), 42.42 (N($\underline{C}H_3)_2$. **EA** calc.%: C: 57.94, H: 6.56, N: 13.51, found%: C: 57.98, H: 6.63, N: 13.25. **MS**: *m/z* 415.1565 [Al(Et)-$(L^4)_2$+H]$^+$

Synthesis of [Al(L^5)$_2$Et] (*13*)

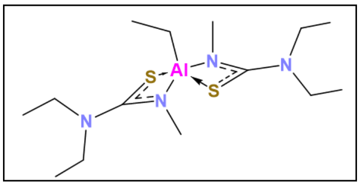

A solution of **L^5H** (1.49 g, 10.2 mmol) in toluene (15 mL) was added to a cooled solution of triethylaluminium (0.70 g, 6.13 mmol) in toluene (15 mL). The reaction mixture was allowed to warm up to RT and then refluxed for 72 h at 115 °C. This yielded a yellow-orange solution which was dried under vacuum to result in a viscous yellow-orange oil. A small amount of toluene was added to this, and the reaction mixture was stored in the glovebox to allow crystal formation. After 3 months, crystals were obtained which were seen to be a mixture of the pure ligand (**L^5H**) as well as those of compound **13**. The formation of **13** was confirmed by SCXRD.
^{1}H NMR δ/ppm (500 MHz, C_6D_6) δ 3.23 – 2.94 (8H, m, N($C\underline{H}_2CH_3)_2$), 2.82 (6H, *s*, N($C\underline{H}_3$)), 1.70 (3H, t, *J* = 8.1 Hz, Al$CH_2C\underline{H}_3$), 1.47 – 1.32 (2H, m, Al$C\underline{H}_2CH_3$), 0.84 (12H, t, *J* = 7.1 Hz, N($CH_2C\underline{H}_3)_2$). Signals for **L^5H** and other unidentified products were also observed in the ^{1}H NMR spectrum of **9**. **^{13}C{^{1}H} NMR** δ/ppm (126 MHz, C_6D_6): 46.32 (N($\underline{C}H_2CH_3)_2$), 35.54 (N($\underline{C}H_3$)), 13.81 (N($CH_2\underline{C}H_3)_2$) 11.24 (Al$CH_2\underline{C}H_3$), 9.63 (Al$\underline{C}H_2CH_3$). **EA** calc.%: C: 48.52, H: 9.02, N: 16.17, found%: C: 48.24, H: 9.18, N: 15.59. **MS**: *m/z* 347.1887 [Al(Et)-$(L^5)_2$+H]$^+$

Synthesis of [Al(L^8)$_2$Et] (*14*)

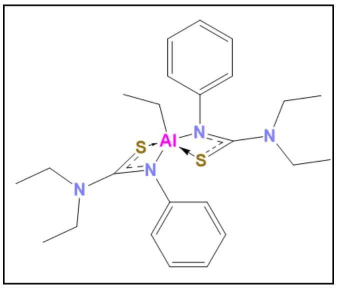

A solution of **L^8H** (2.12 g, 10.2 mmol) in toluene (15 mL) was added to a solution of triethylaluminium (0.70 g, 6.13 mmol) in toluene (15 mL). The reaction mixture was allowed to warm up to RT then heated to 115 °C and refluxed for 72 h. A deep brown-yellow solution was observed which was filtered to get rid of any unreacted material and then reduced *in vacuo* to yield a viscous oil. To this a small amount of toluene was added and this was stored in the glovebox to allow crystal formation. After 3 months, clear crystals of **14**, suitable for SCXRD were obtained. Yield of single crystals: 14.2%
^{1}H NMR δ/ppm (500 MHz, C_6D_6) 7.15 – 6.83 (10H, m, Ph-$\underline{H}$), 3.04 – 2.88 (8H, m, N($C\underline{H}_2CH_3)_2$), 1.77 (3H, t, *J* = 8.1 Hz, Al$CH_2C\underline{H}_3$), 1.00 (2H, q, *J* = 8.0 Hz, Al$C\underline{H}_2CH_3$), 0.68 (12H, t, *J* = 7.1 Hz, N($CH_2C\underline{H}_3)_2$). **^{13}C{^{1}H} NMR** δ/ppm (126 MHz, C_6D_6) δ 177.65 (Ph-$\underline{C}$), 146.15 (Ph-$\underline{C}$), 46.72 (N($\underline{C}H_2CH_3)_2$), 12.85 (Al$CH_2\underline{C}H_3$), 11.70 (N$CH_2\underline{C}H_3)_2$). **EA** calc.%: C: 61.24, H: 7.50, N: 11.90, found%: C: 61.05, H: 7.51, N: 11.77. **MS**: *m/z* 471.2191 [Al(Et)-$(L^8)_2$+H]$^+$

d. Synthesis of trimethylamine alane

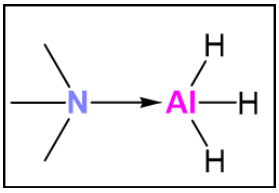

TMAA was synthesised following literature procedures.[7] $LiAlH_4$ (3.19 g, 84 mmol) and dried trimethylamine hydrochloride (6.69 g, 70 mmol) were combined and cooled to −78 °C. To this, cold diethyl ether (40 mL) was slowly added. After 10 min a white suspension had formed which was allowed to warm up to RT and stirred overnight. The white suspension was filtered to yield a colourless transparent solution. This was reduced *in vacou* to produce a white solid. Purification of the white solid by sublimation yielded TMAA.

^{1}H NMR δ/ppm (300 MHz, C_6D_6): 4.13 (6H, *s* (br), $Al_2\underline{H}_6$), 1.83 (18H, *s*, $C\underline{H}_3$). NMR data matches NMR spectra reported in the literature.[7]

2. Crystallographic data

a. Crystallographic data for **2**

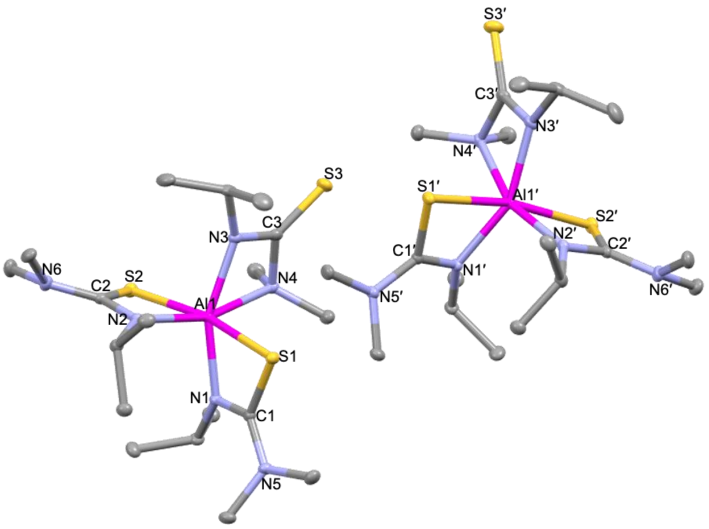

Figure S1: Solid state structure of **2** displaying both molecules. Hydrogen atoms omitted for clarity. Thermal ellipsoids drawn at 20% probability.

Table S1: Comparison of bond lengths and angles for the two molecules of **2**

Compound	**2**		
Length (Å)			
Al–S1	2.3906(6)	Al′–S1′	2.3857(6)
Al–S2	2.3911(6)	Al′–S2′	2.3917(6)
Al–N1	2.000(1)	Al′–N1′	1.994(1)
Al–N2	1.995(1)	Al′–N2′	1.997(1)
Al–N3	2.001(1)	Al′–N3′	1.992(1)
Al–N4	2.158(1)	Al′–N4′	2.157(1)
Angle (°)		Angle (°)	
S1–Al–N1	70.56(4)	S1′–Al′–N1′	70.86(4)
S2–Al–N2	70.93(4)	S2′–Al′–N2′	70.83(4)
N3–Al–N4	64.77(5)	N3′–Al′–N4′	64.90(5)
S1–Al–S2	168.50(2)	S1′–Al′–S2′	168.97(3)

b. Crystallographic data for **10**

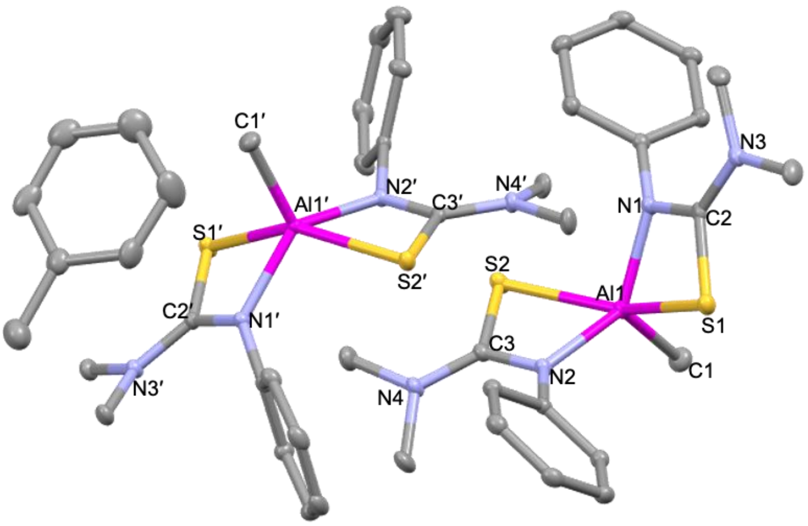

Figure S2: Solid state structure of **10** showing both molecules and the crystallised toluene molecule. Hydrogen atoms omitted for clarity. Thermal ellipsoids drawn at 20% probability.

Table S2: Comparison of bond lengths and angles for the two molecules of **10**.

Compound	**10**		
Length (Å)			
Al–S1	2.4315(6)	Al′–S1′	2.4644(5)
Al–S2	2.4639(6)	Al′–S2′	2.4113(6)
Al–N1	1.933(1)	Al′–N1′	1.926(1)
Al–N2	1.922(2)	Al′–N2′	1.927(1)
Al–C1	1.959(2)	Al′–C1′	1.960(2)
Angle (°)		Angle (°)	
S1–Al–N1	70.61(4)	S1′–Al′–N1′	70.08(4)
S2–Al–N2	70.05(4)	S2′–Al′–N2′	71.19(4)
S1–Al–C1	104.68(6)	S1′–Al′–C1′	104.95(5)
S2–Al–C1	103.62(6)	S2′–Al′–C1′	105.89(5)

3. NMR spectra of compounds **1** – **14**

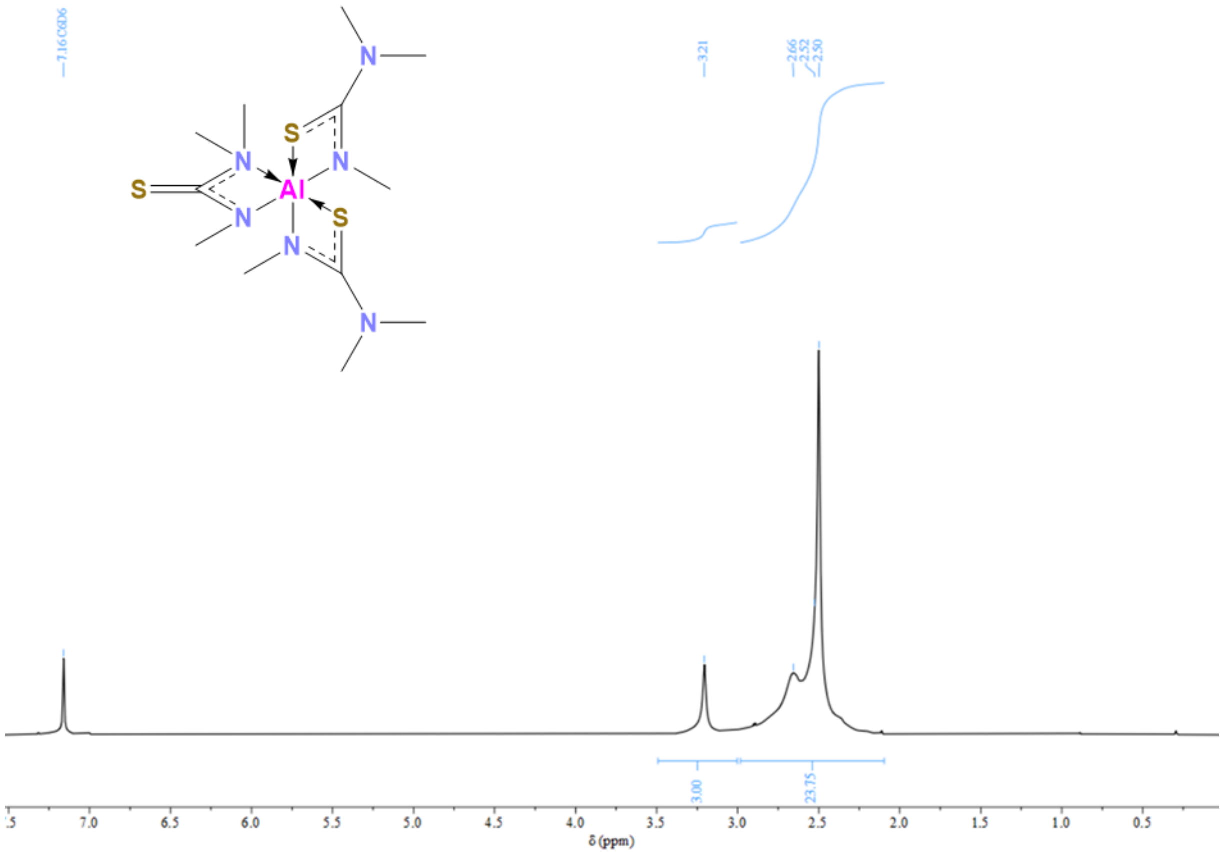

Figure S3: ^{1}H NMR spectrum of compound **1**.

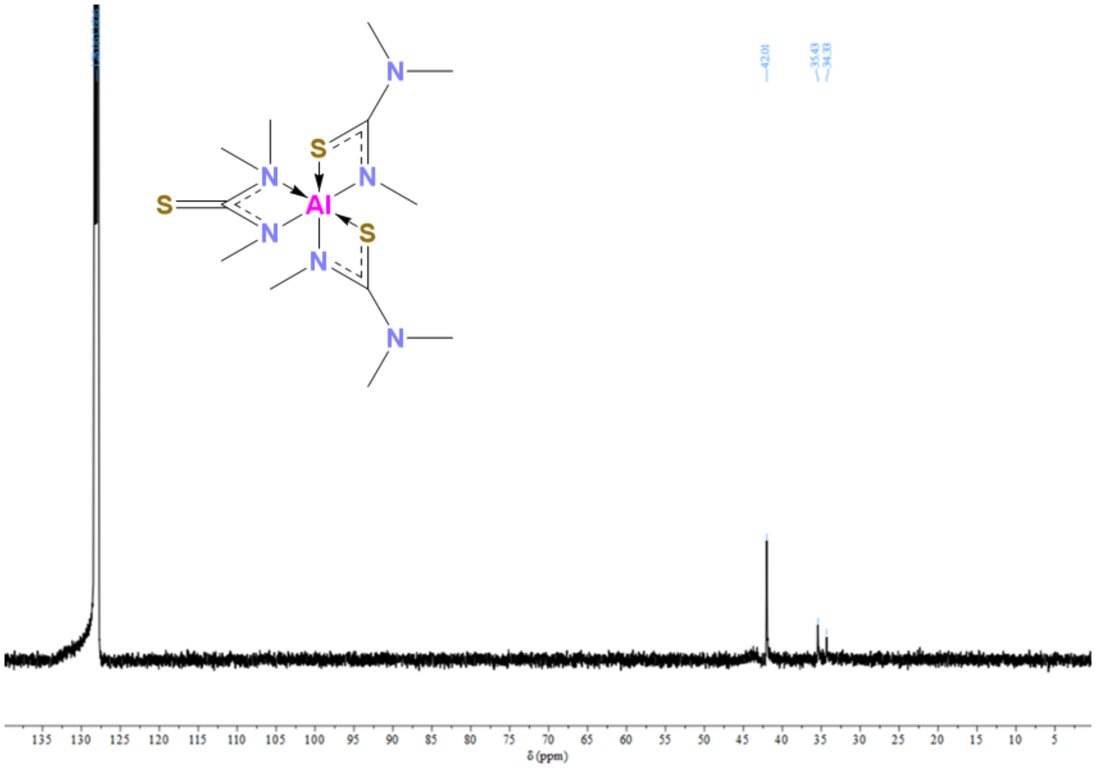

Figure S4: ^{13}C NMR spectrum of compound **1**.

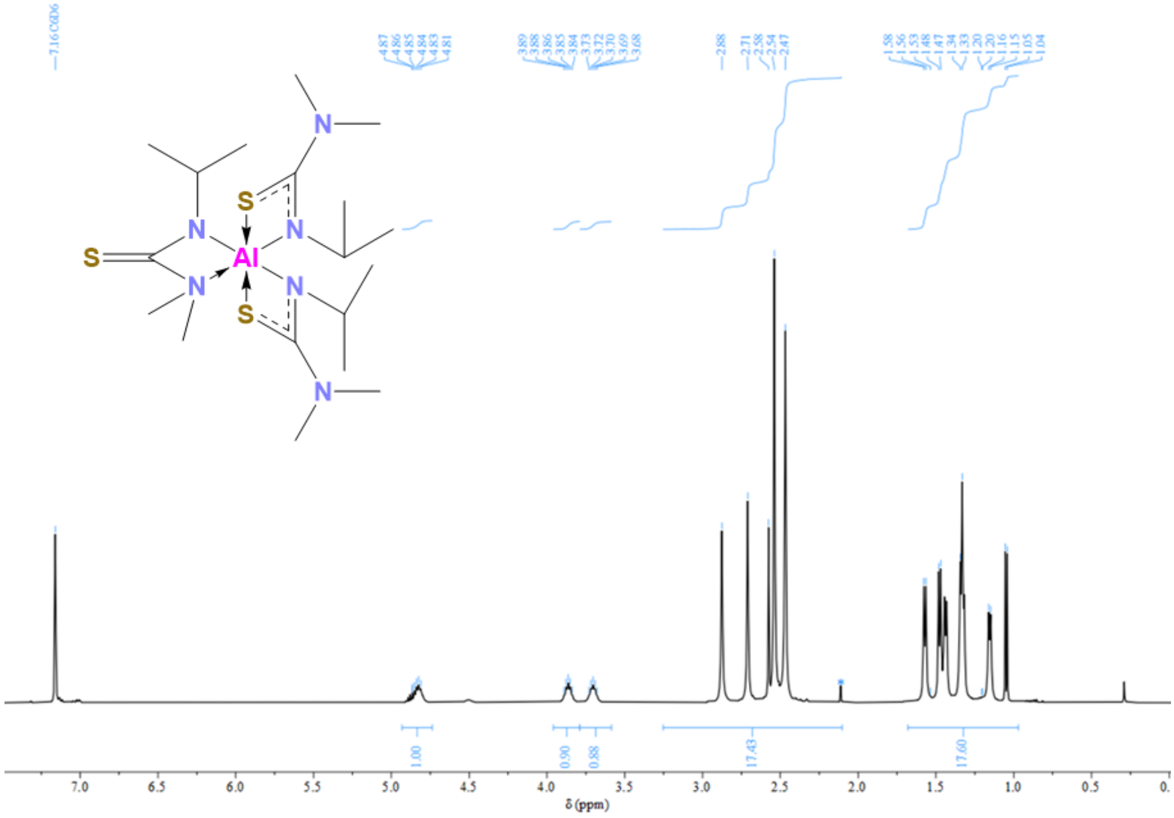

Figure S5: ^{1}H NMR spectrum of compound **2**.

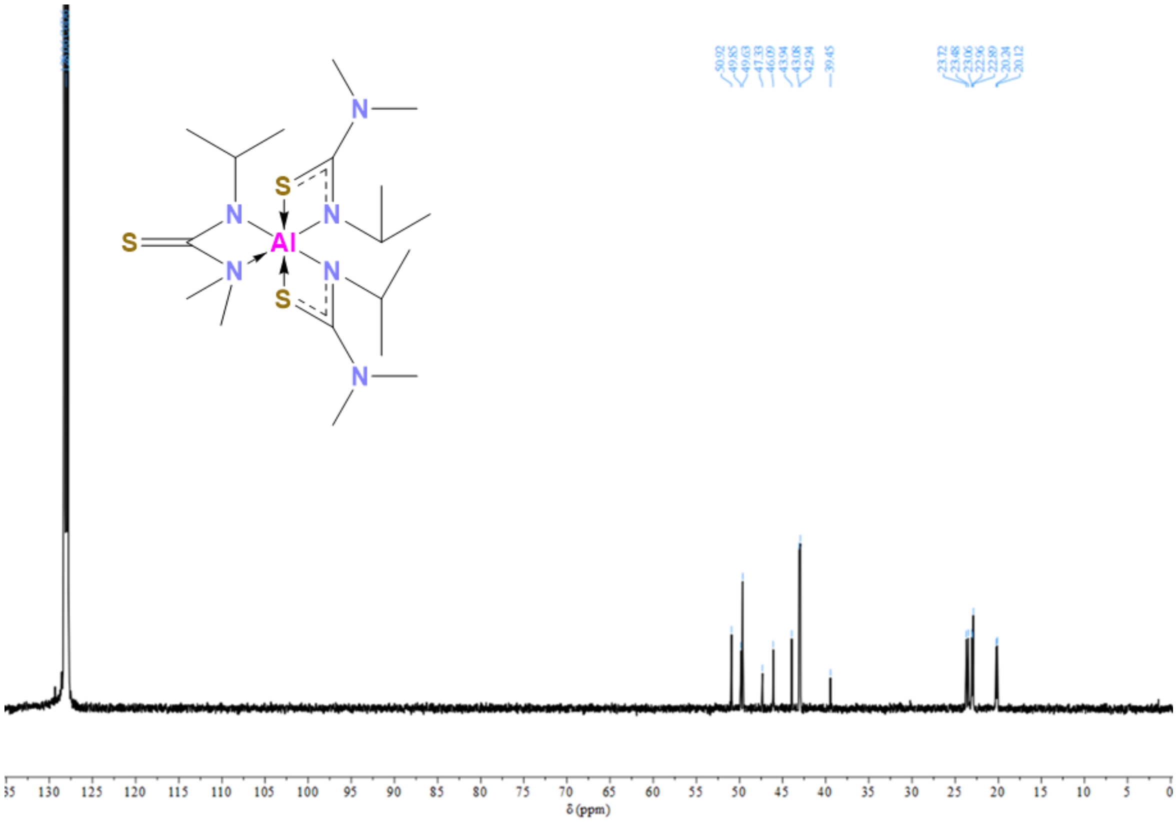

Figure S6: ^{13}C NMR spectrum of compound **2**.

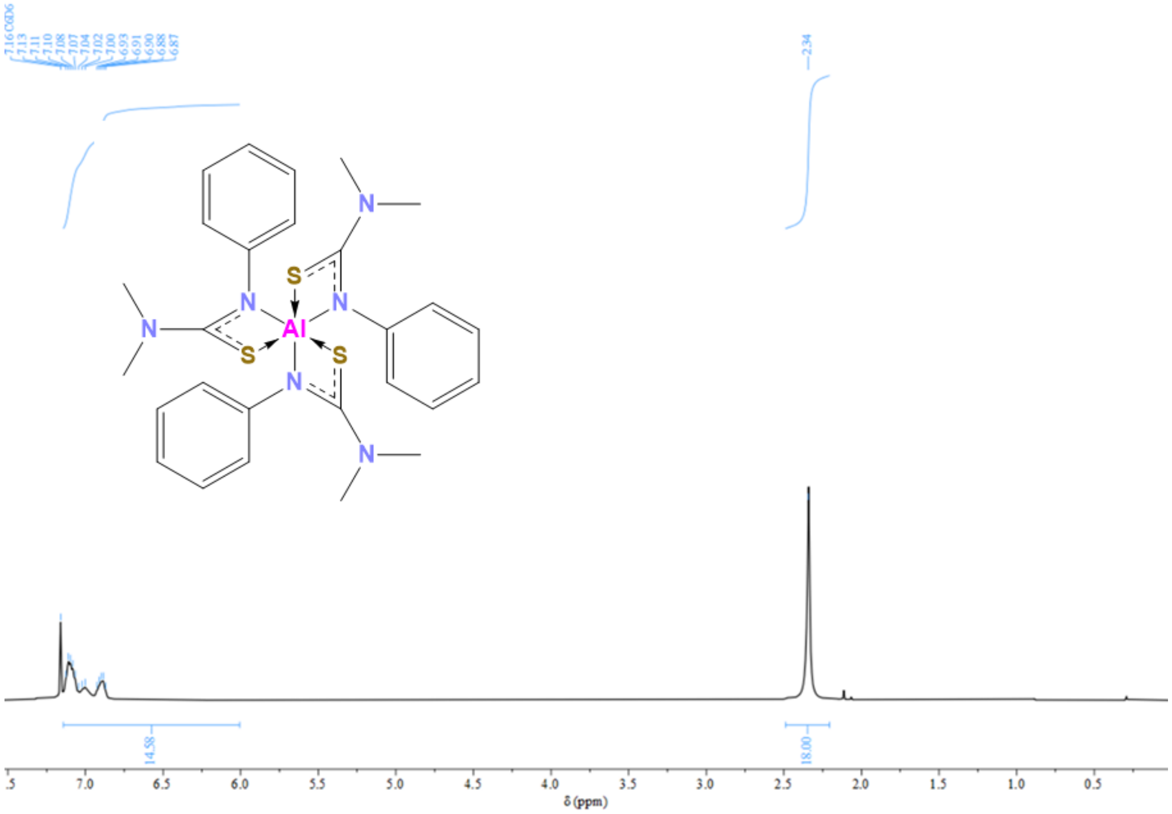

Figure S7: ^{1}H NMR spectrum of compound **3**.

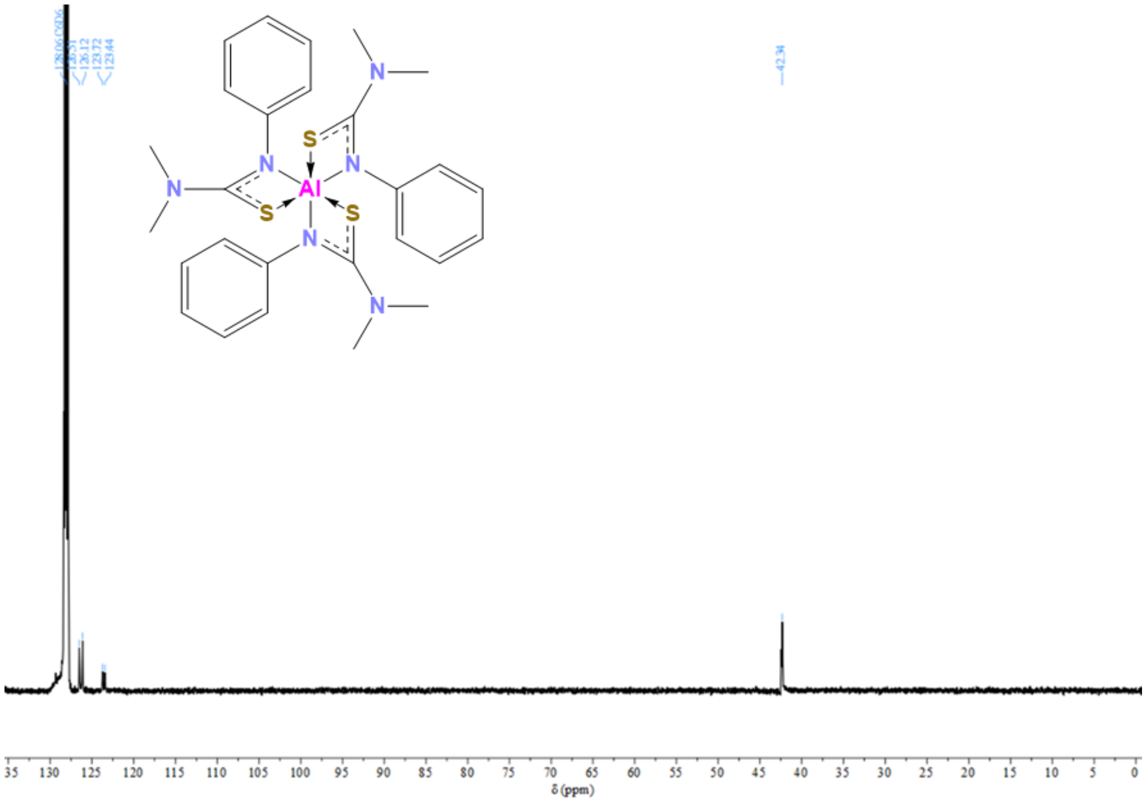

Figure S8: ^{13}C NMR spectrum of compound **3**.

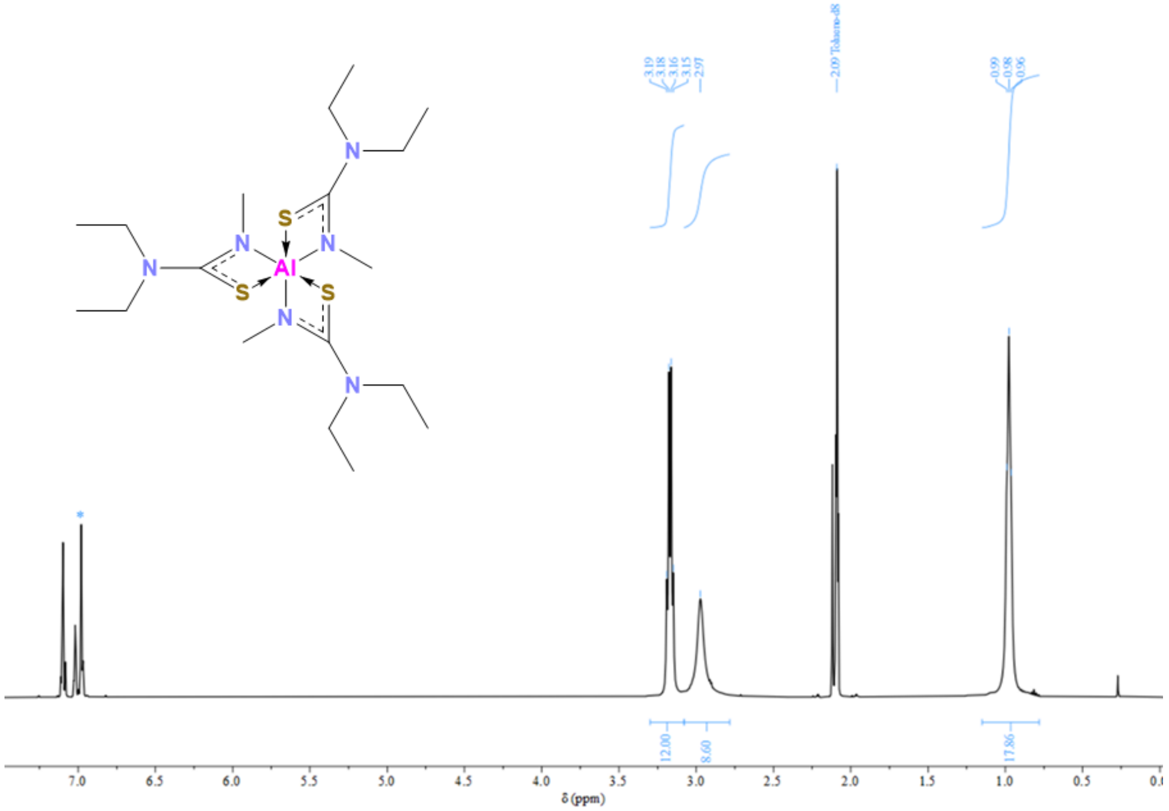

Figure S9: ^{1}H NMR spectrum of compound **4**.

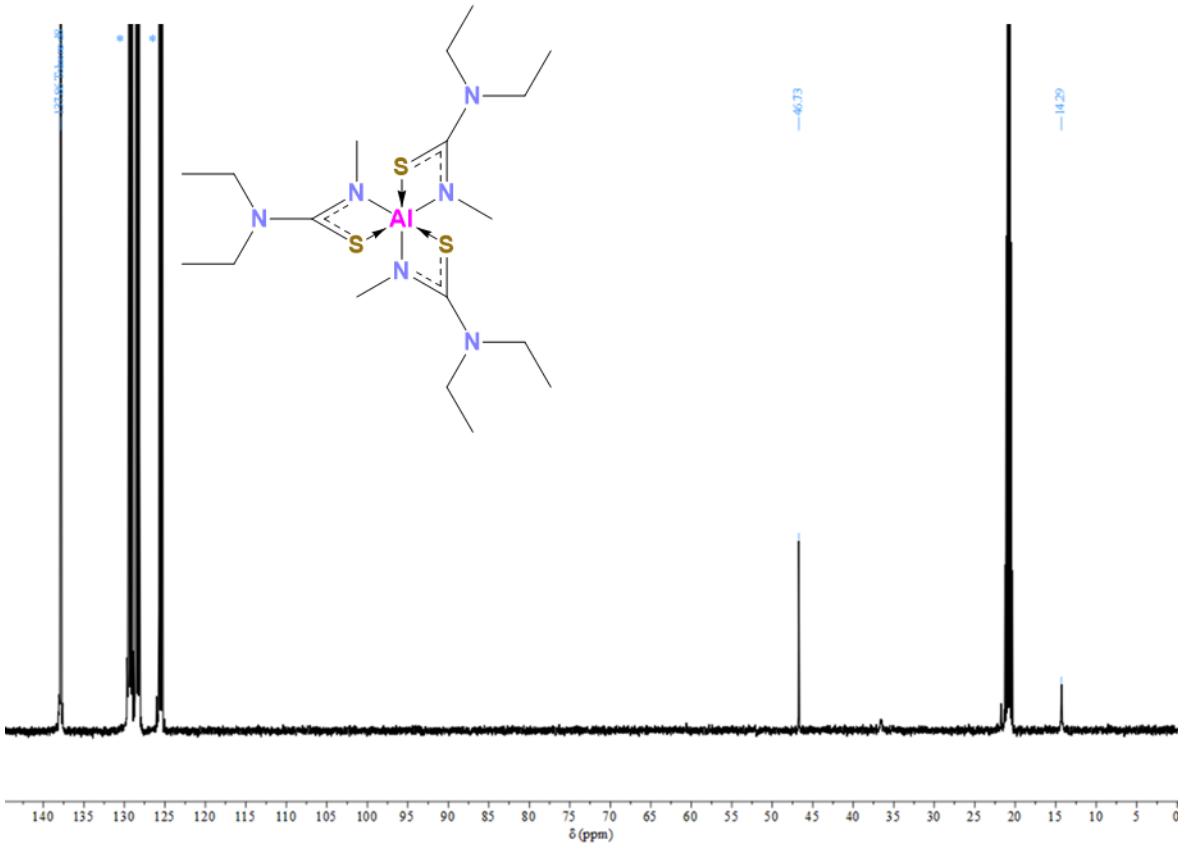

Figure S10: ^{13}C NMR spectrum of compound **4**.

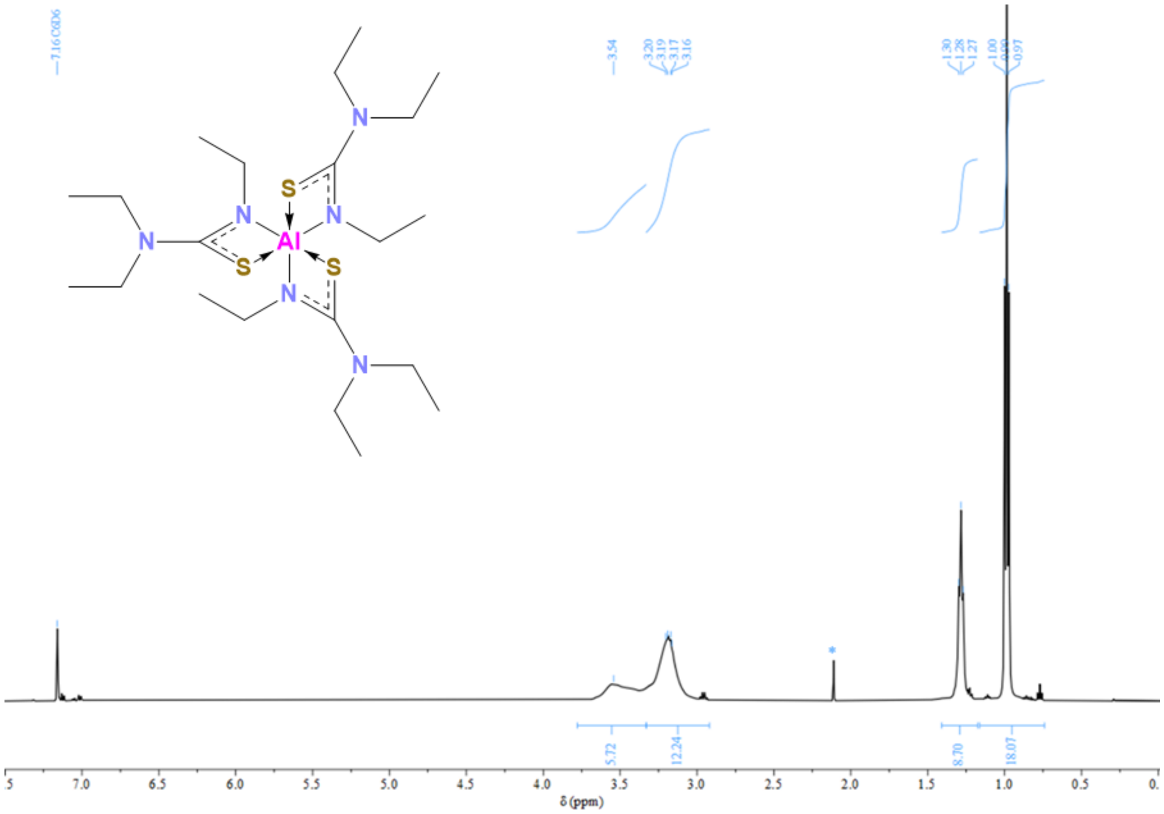

Figure S11: ^{1}H NMR spectrum of compound **5**.

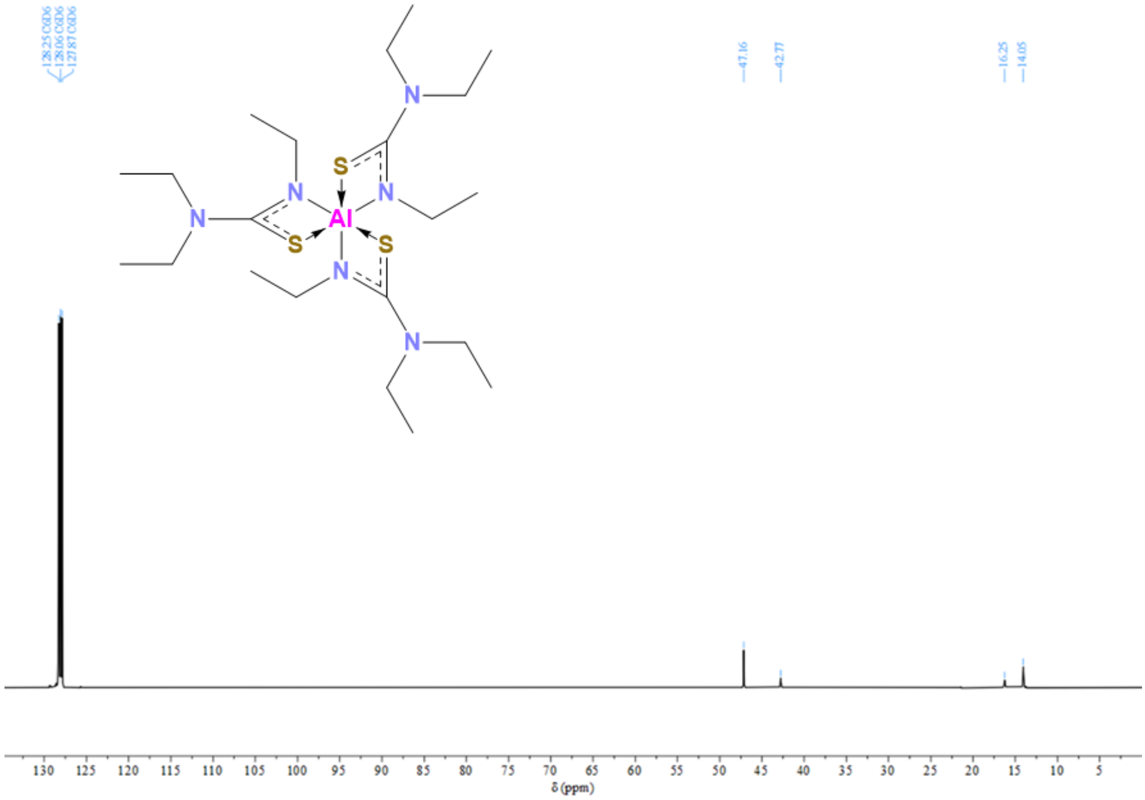

Figure S12: ^{13}C NMR spectrum of compound **5**.

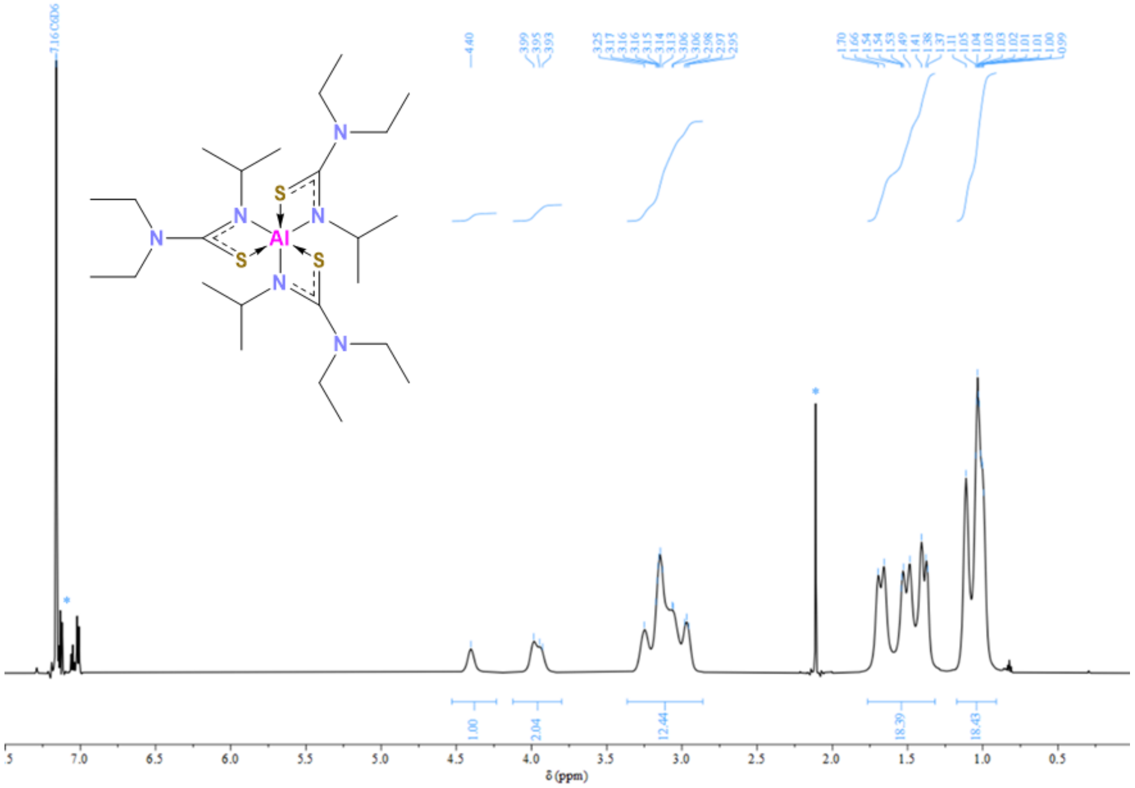

Figure S13: ^{1}H NMR spectrum of compound **6**.

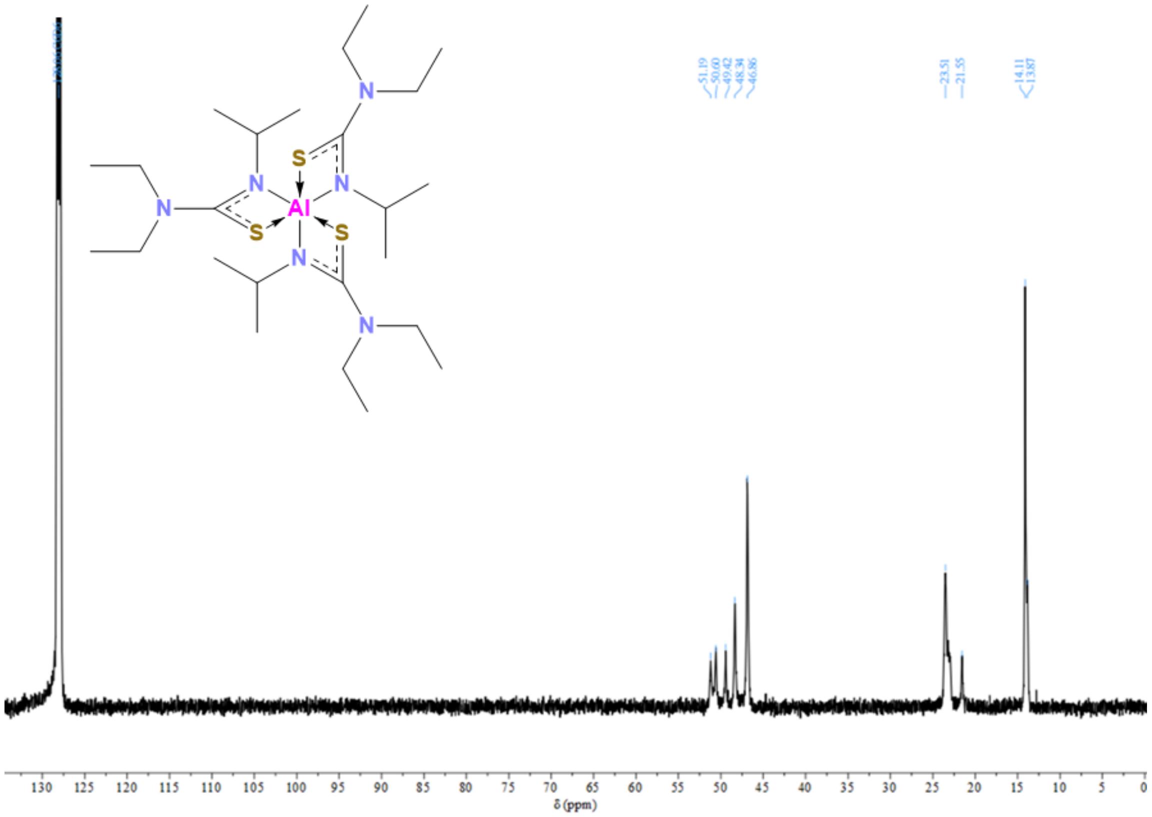

Figure S14: ^{13}C NMR spectrum of compound **6**

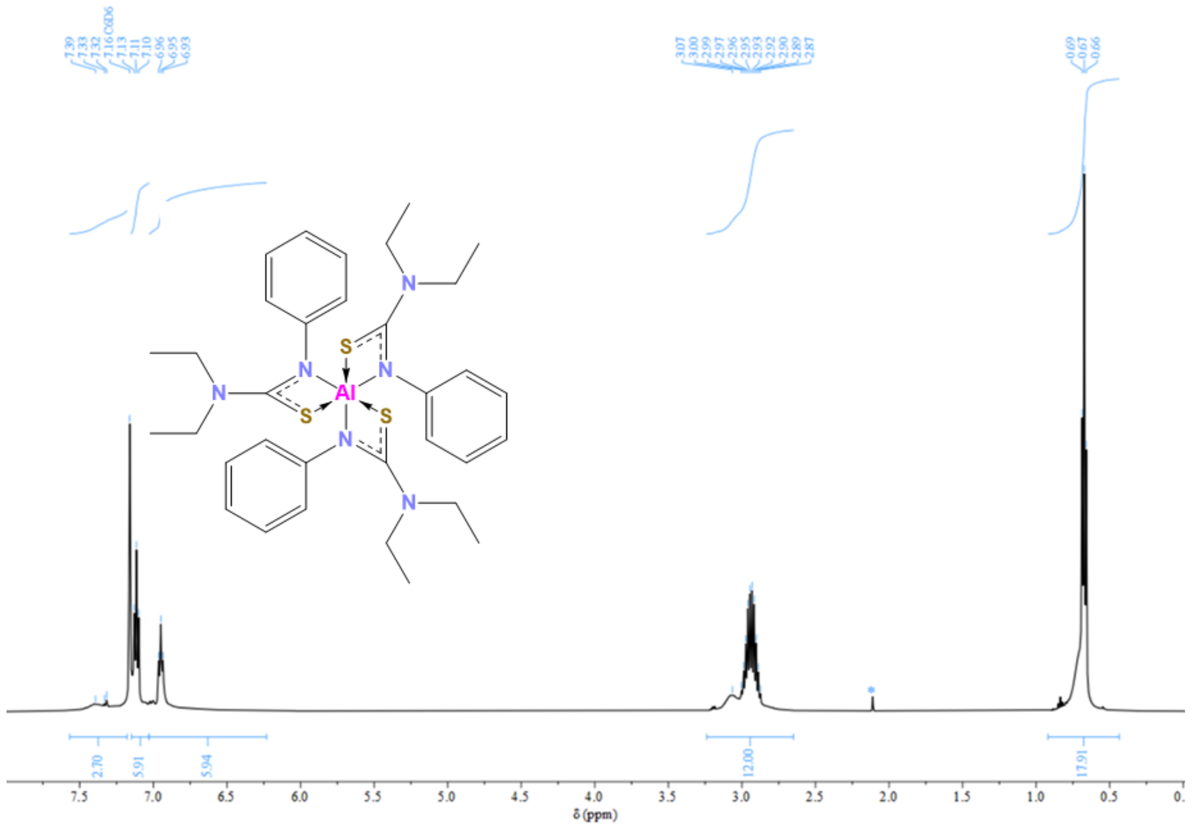

Figure S15: ^{1}H NMR spectrum of compound **7**.

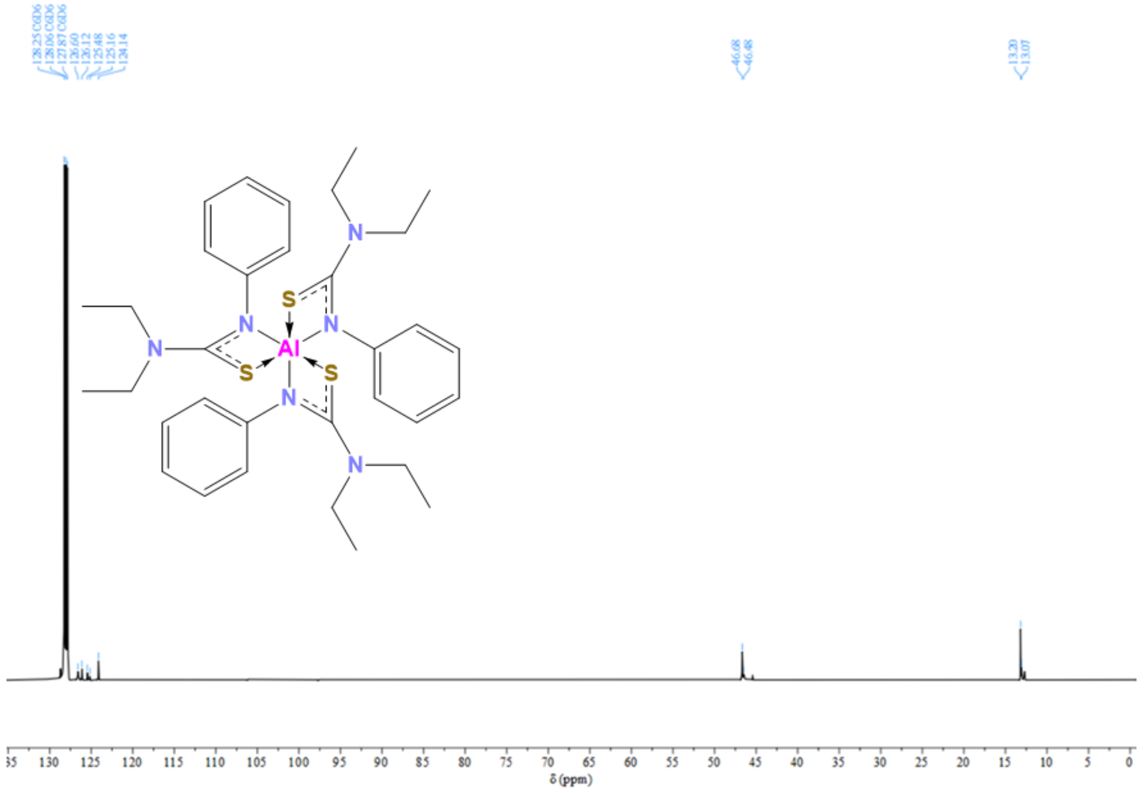

Figure S16: ^{13}C NMR spectrum of compound **7**.

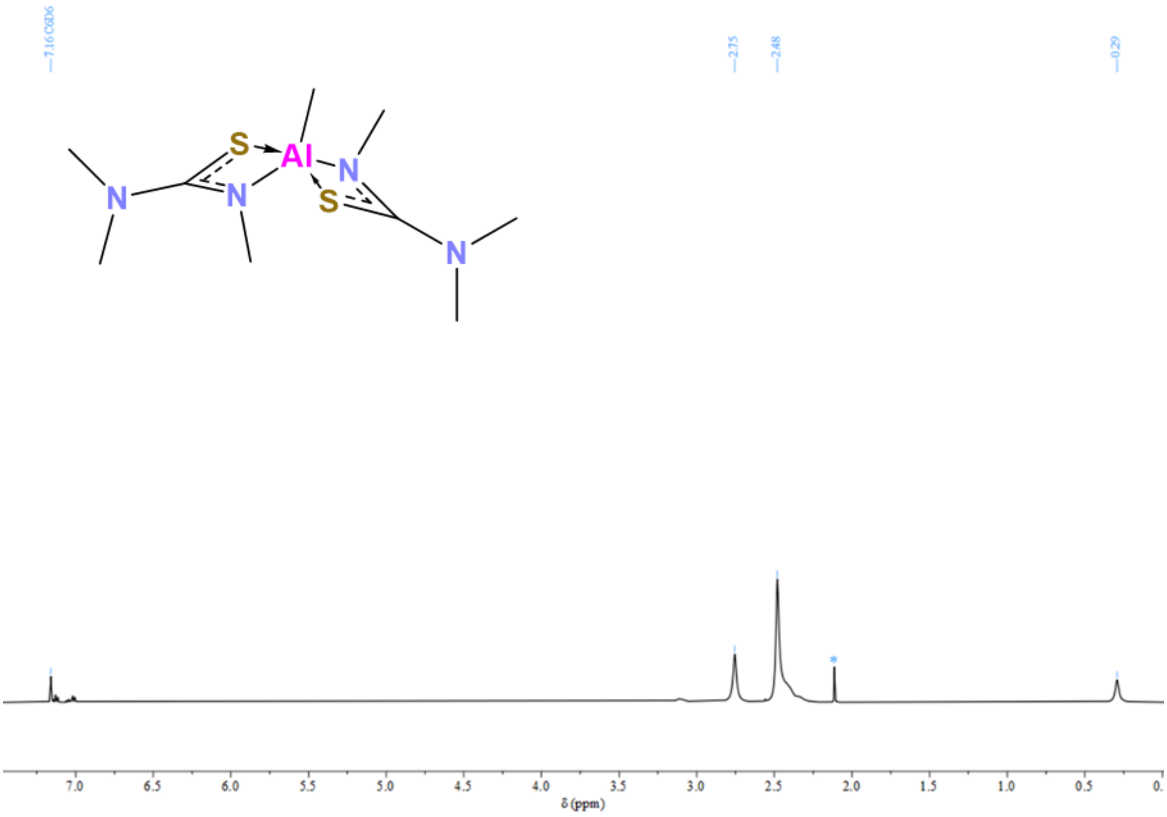

Figure S17: ^{1}H NMR spectrum of compound **8**.

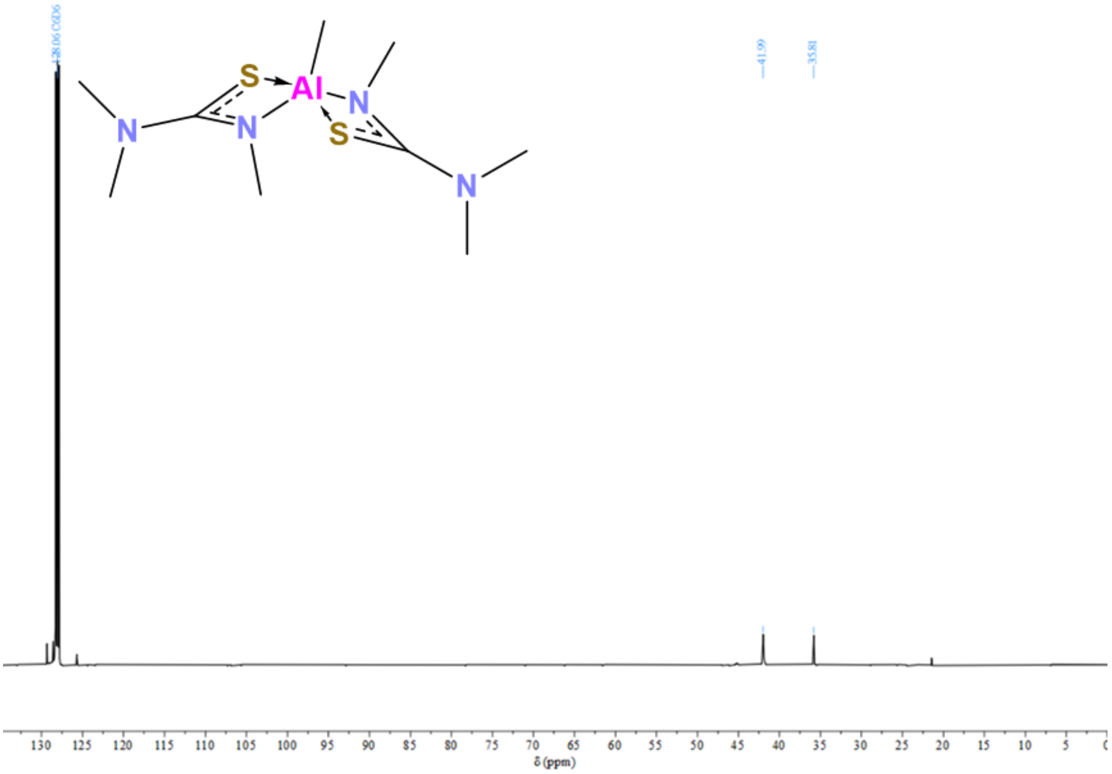

Figure S18: ^{13}C NMR spectrum of compound **8**.

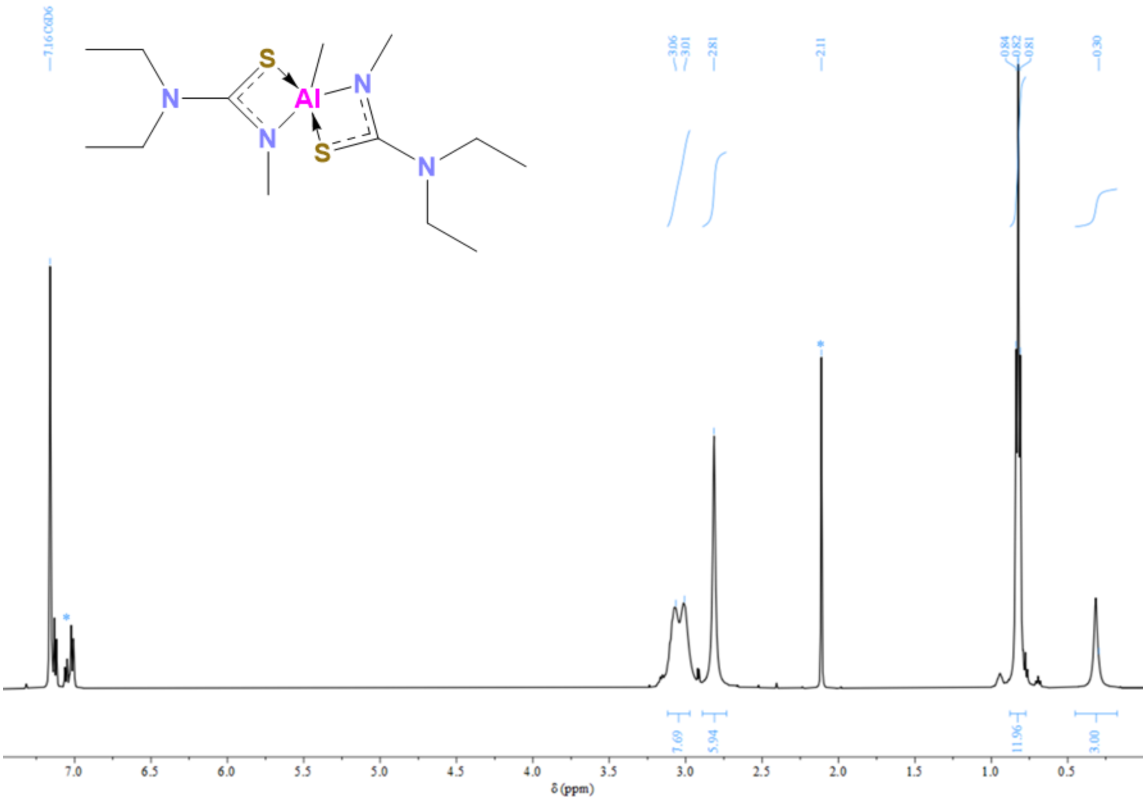

Figure S19: ^{1}H NMR spectrum of compound **9**.

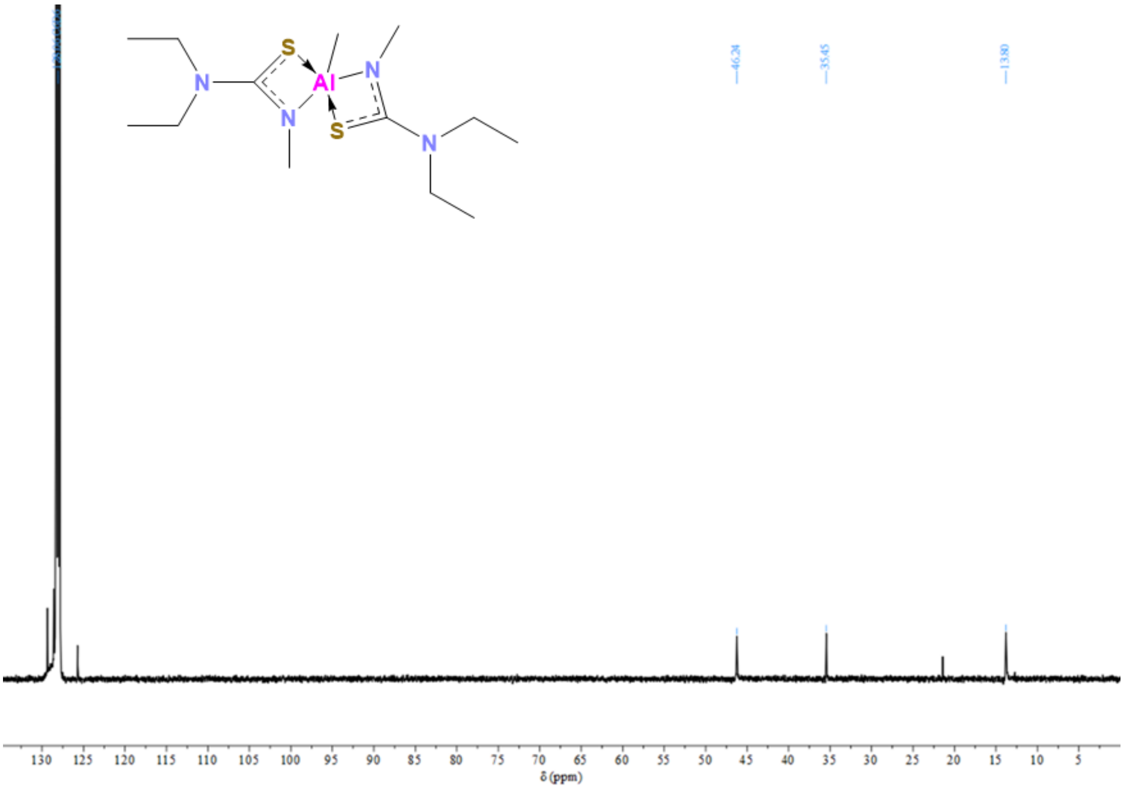

Figure S20: ^{13}C NMR spectrum of compound **9**.

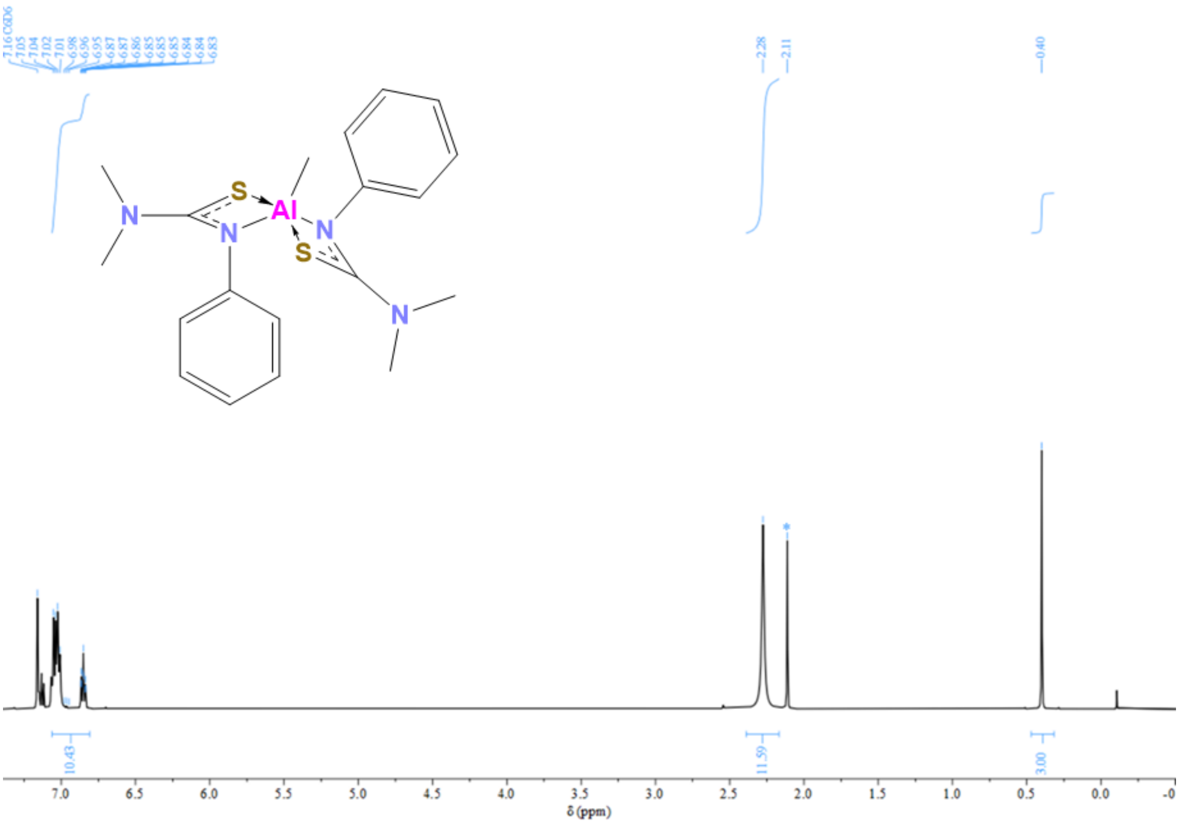

Figure S21: ^{1}H NMR spectrum of compound **10**.

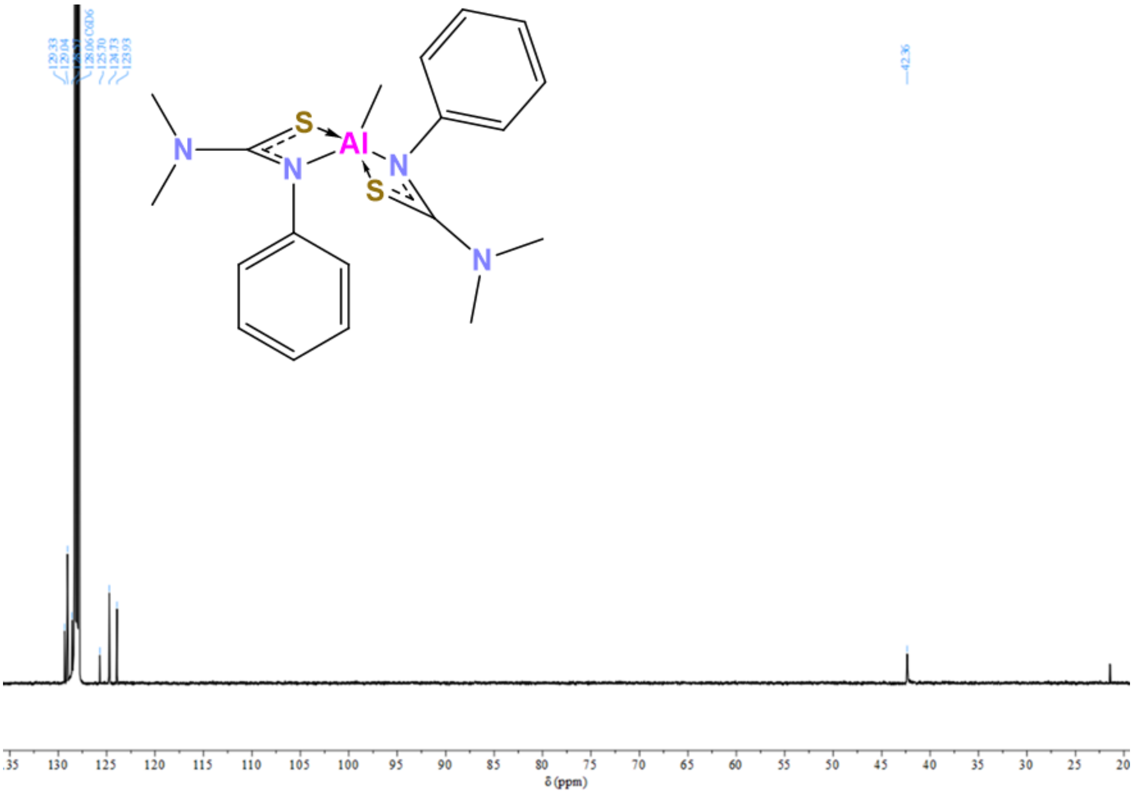

Figure S22: ^{13}C NMR spectrum of compound **10**.

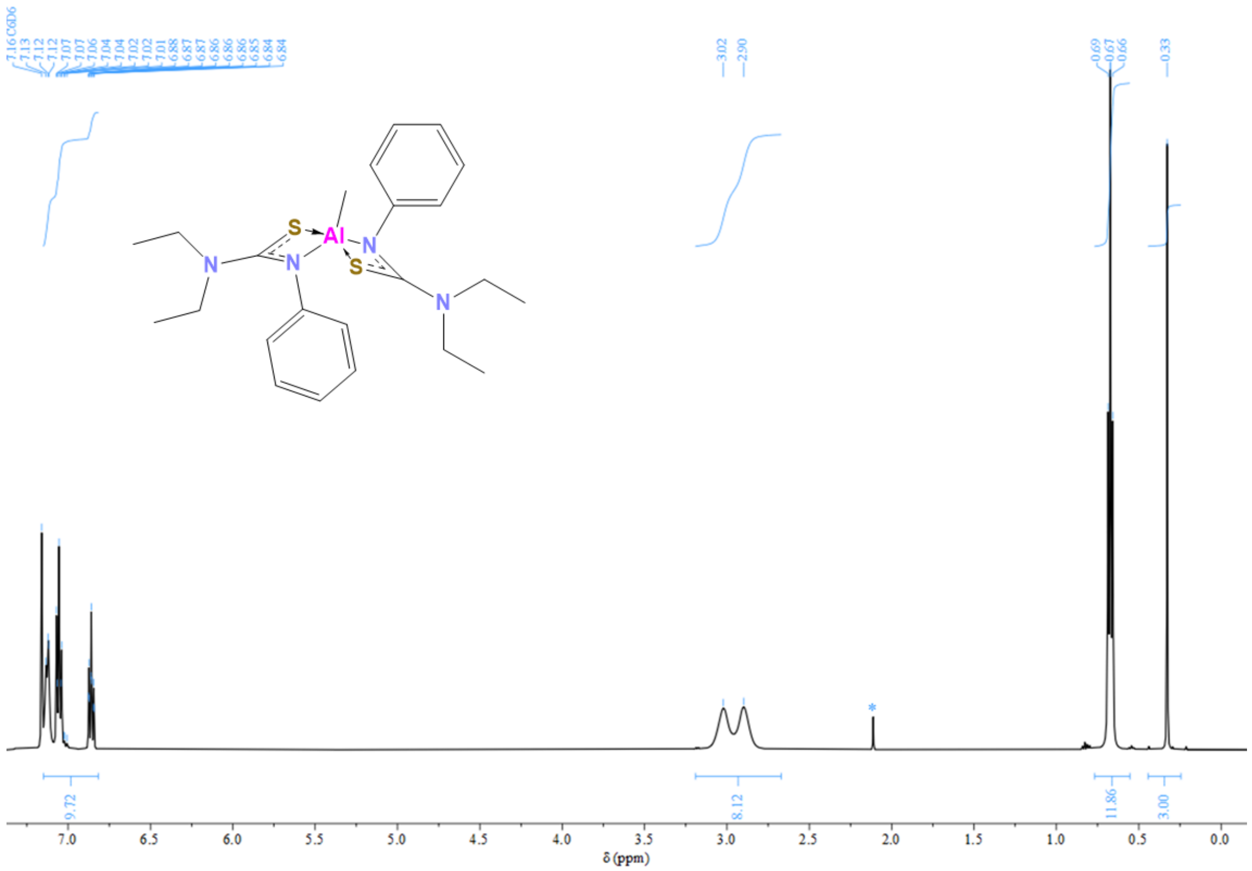

Figure S23:^{1}H NMR spectrum of compound **11**.

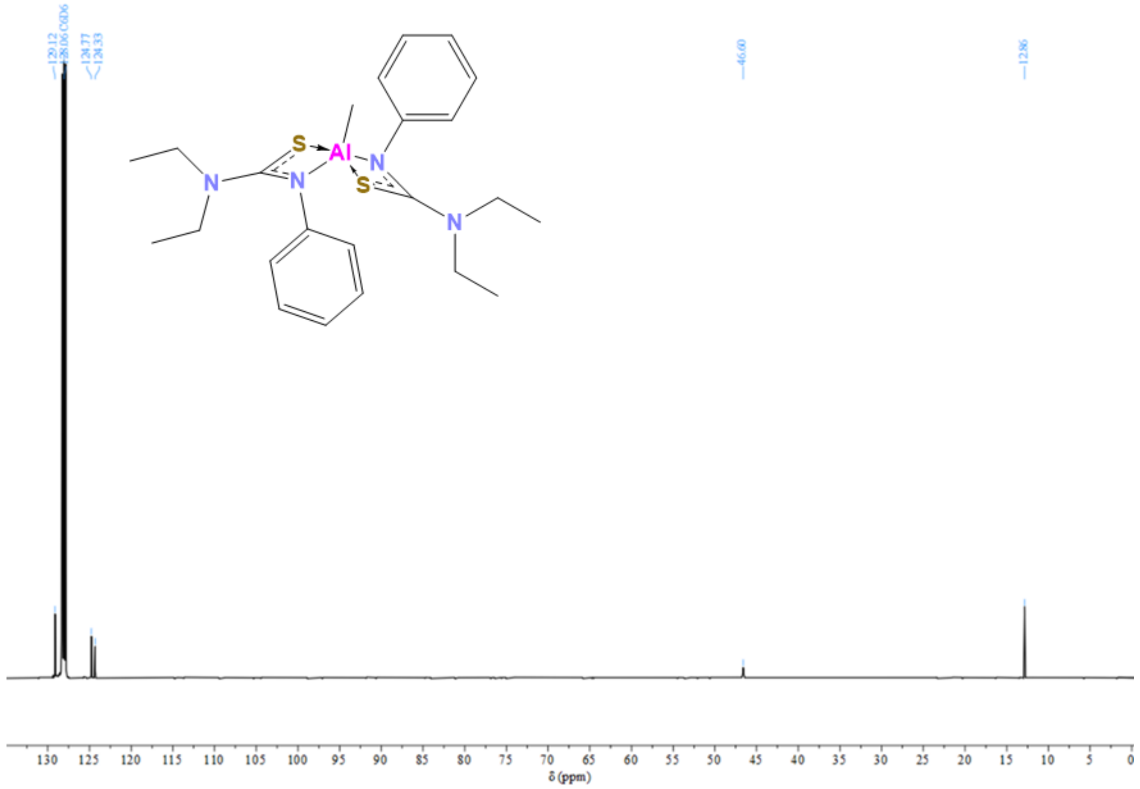

Figure S24:^{13}C NMR spectrum of compound **11**.

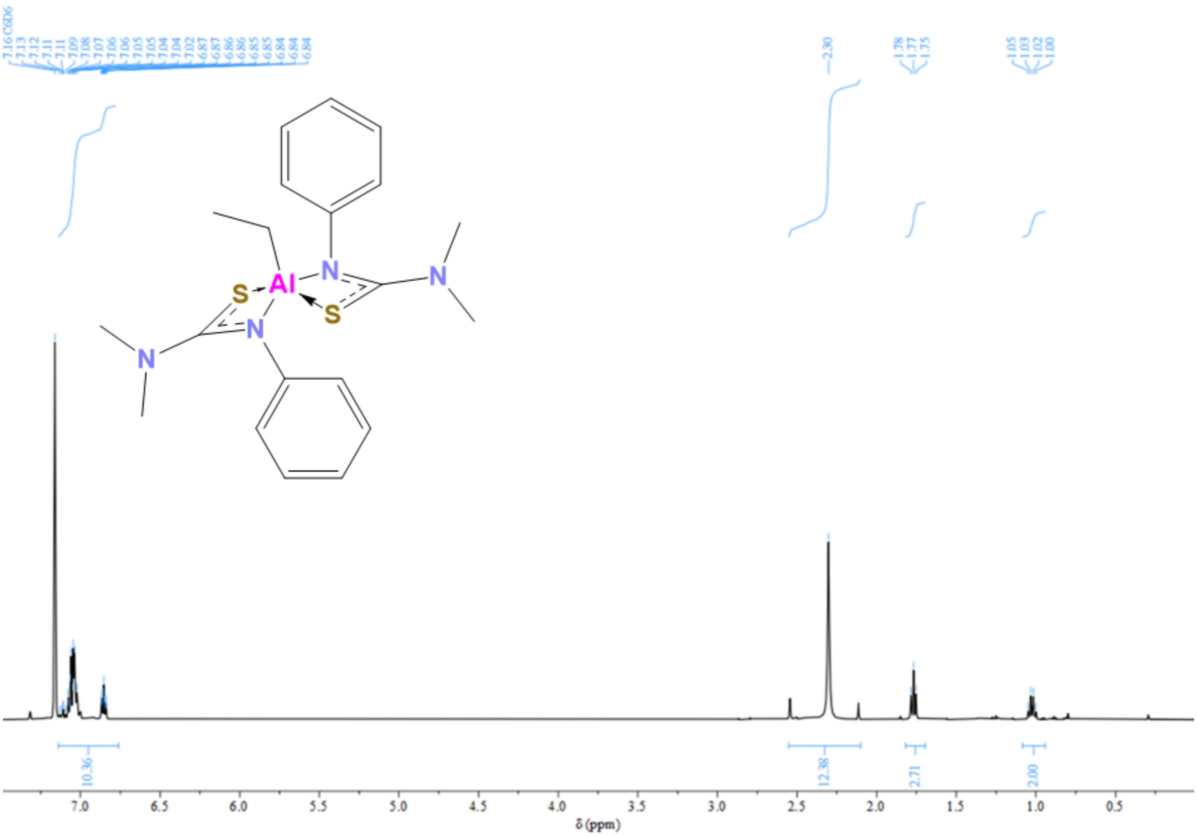

Figure S25: ^{1}H NMR spectrum of compound **12**.

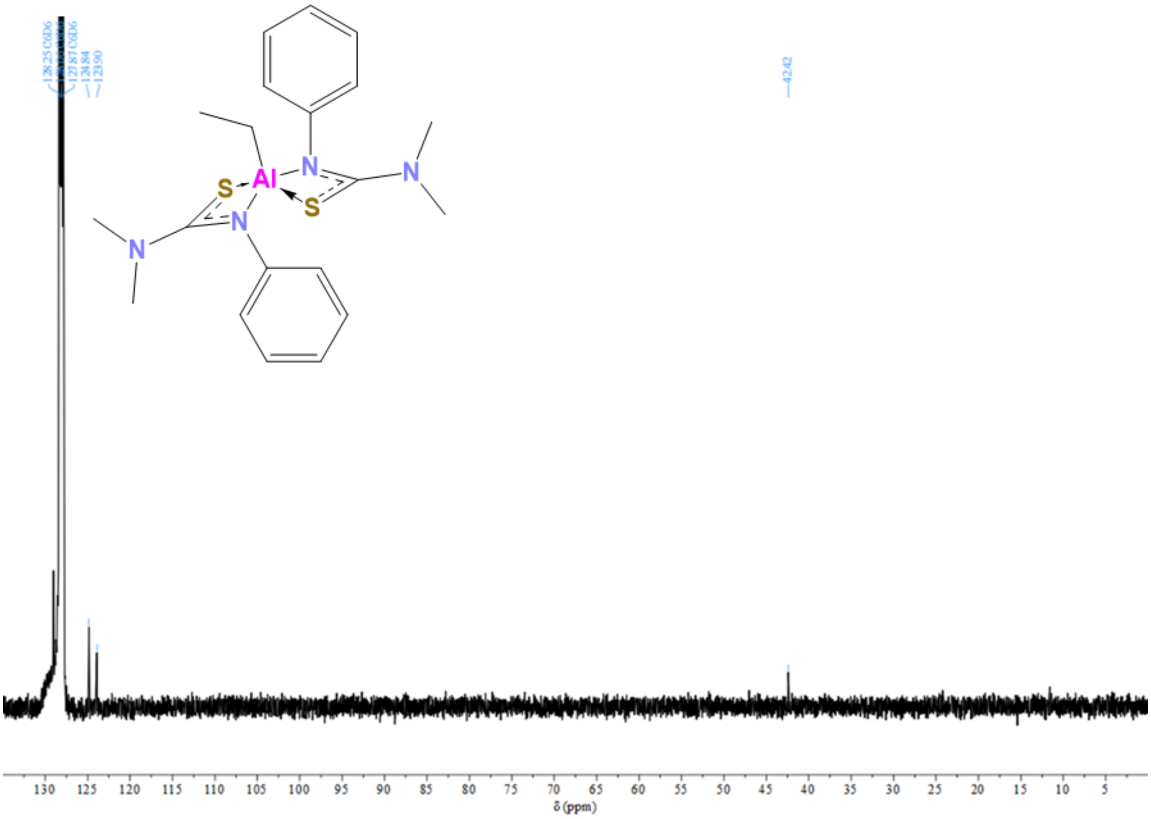

Figure S26: ^{13}C NMR spectrum of compound **12**.

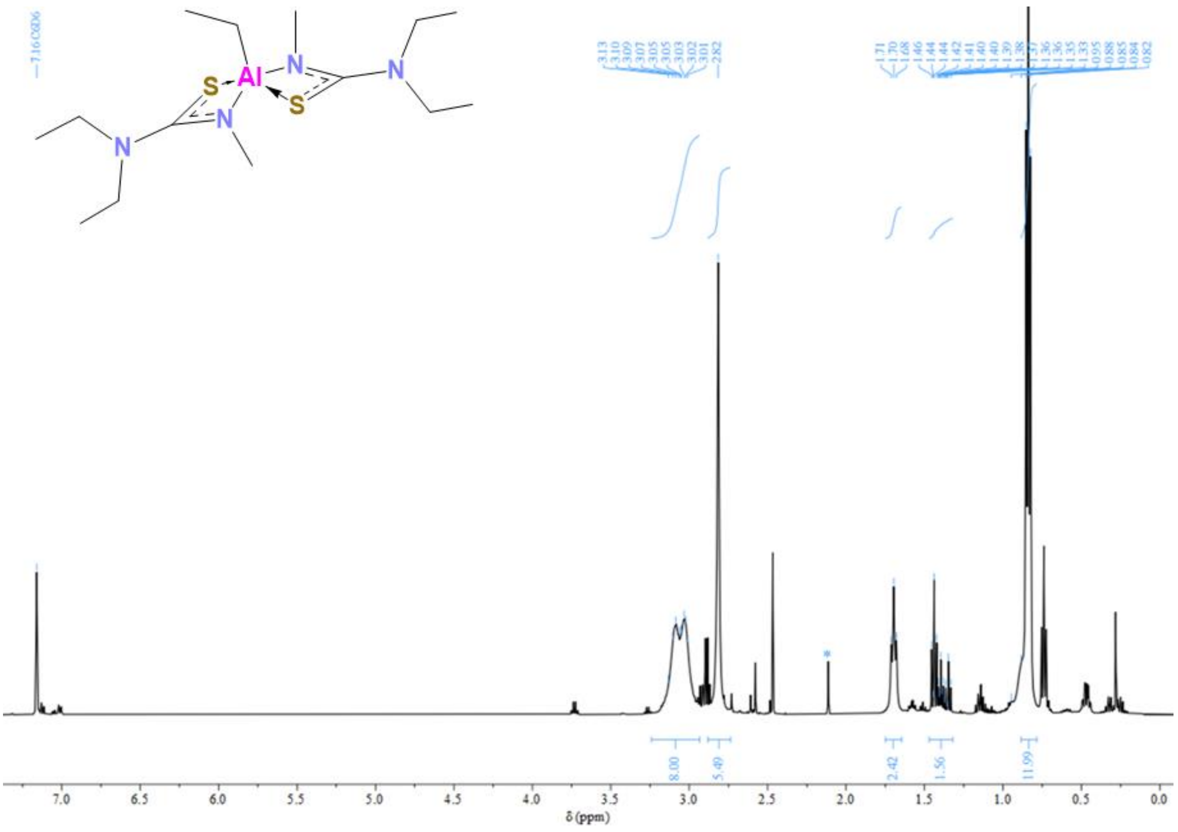

Figure S27: ^{1}H NMR spectrum of compound **13**.

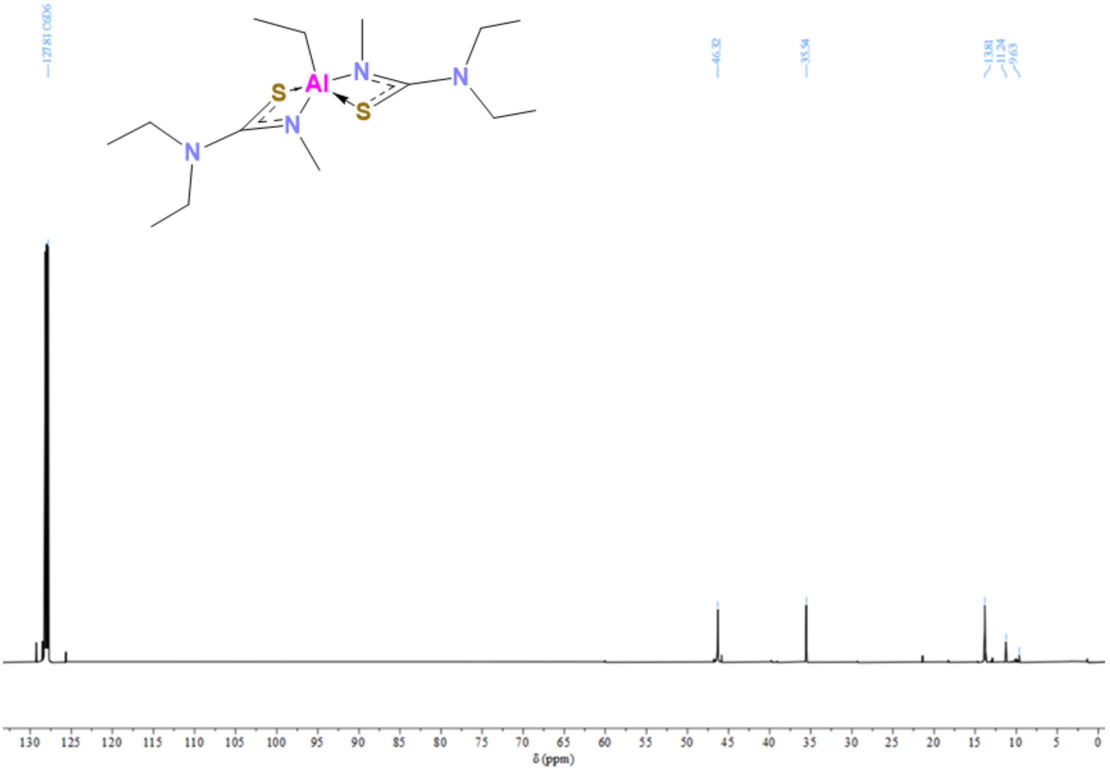

Figure S28: ^{13}C NMR spectrum of compound **13**.

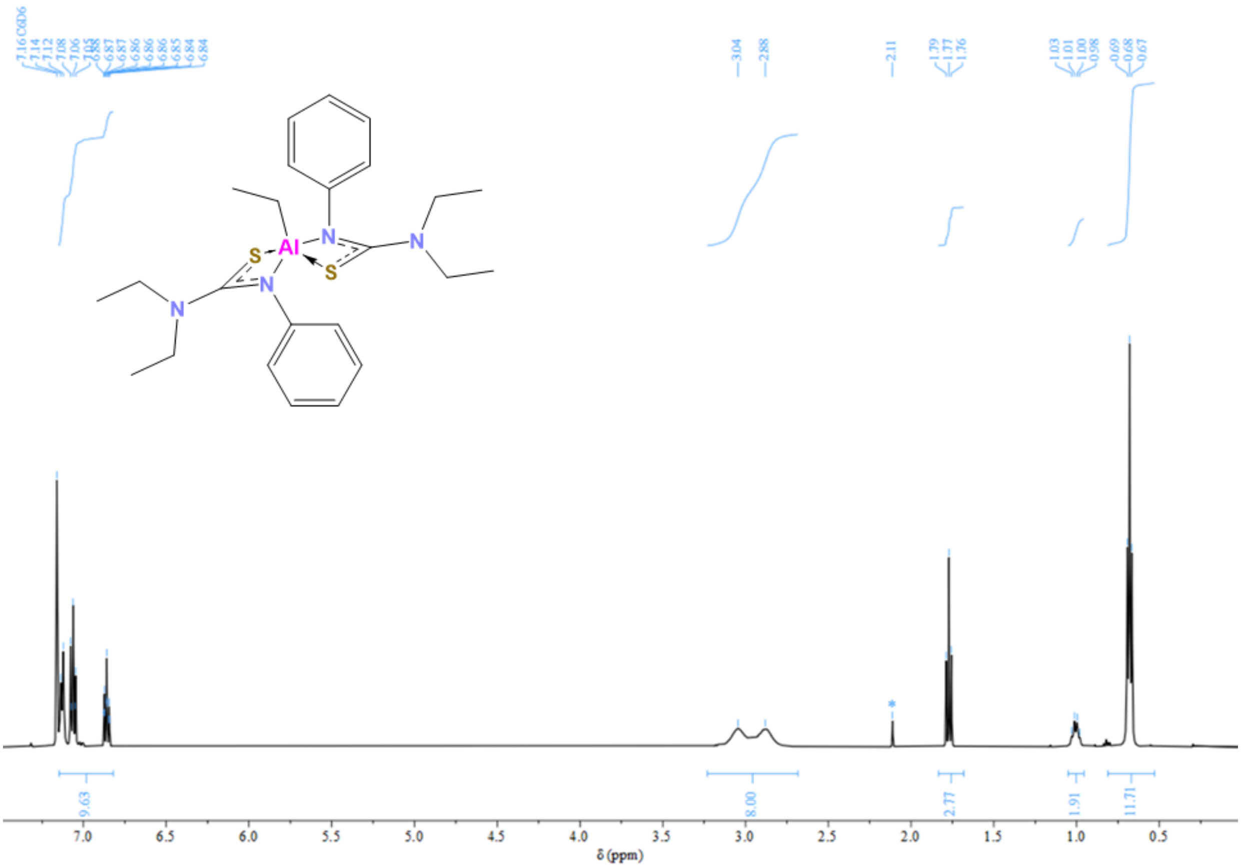

Figure S29: ^{1}H NMR spectrum of compound **14**.

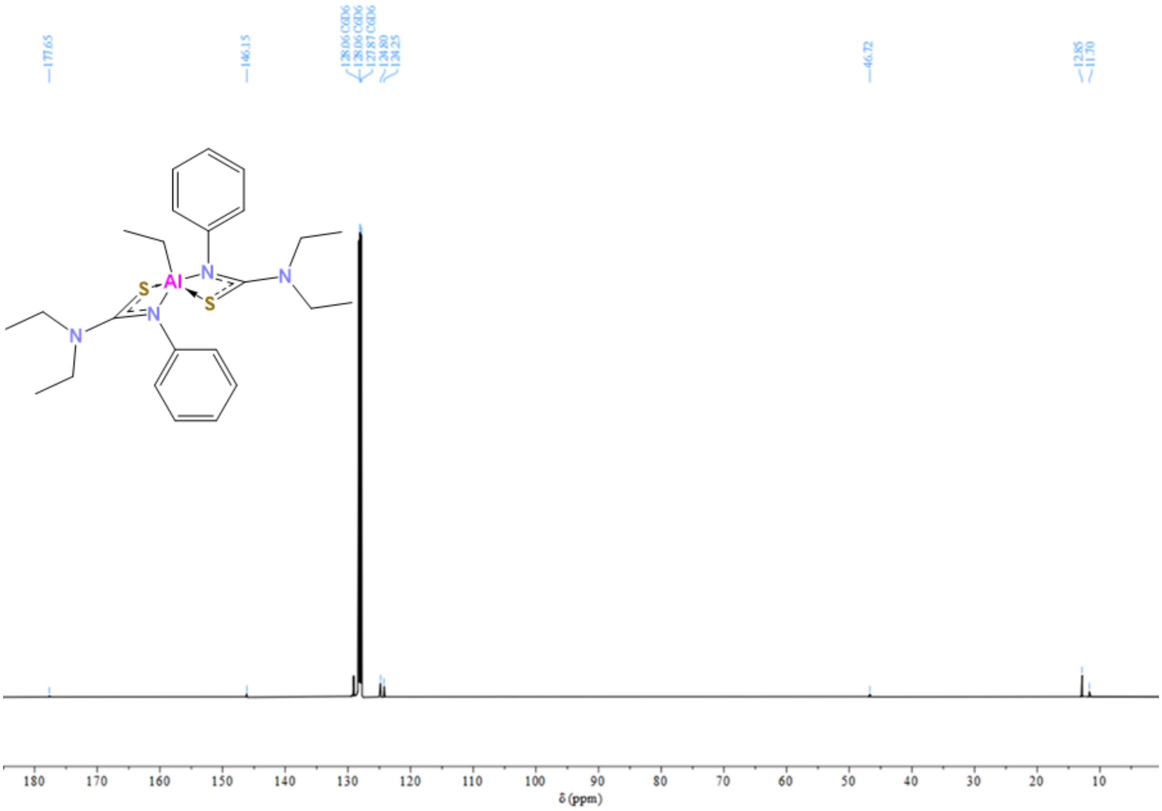

Figure S30: ^{13}C NMR spectrum of compound **14**.

4. **MSMS spectra of compounds 1 – 12**

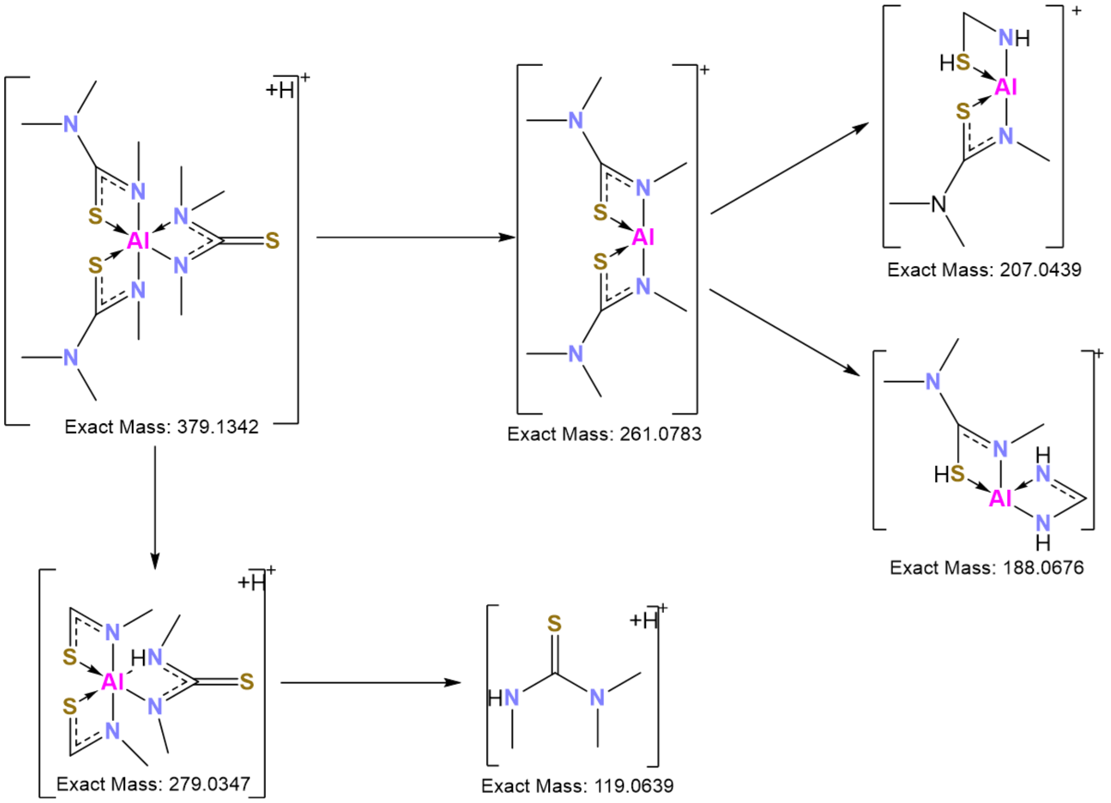

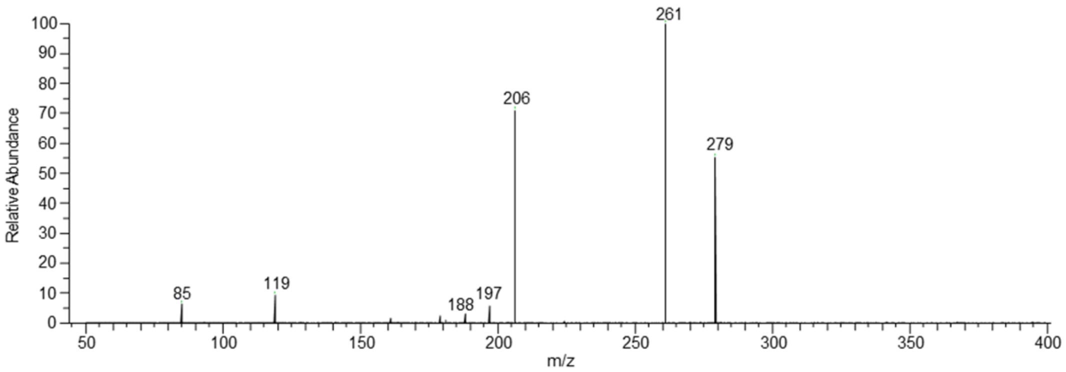

Figure S31: Top: Proposed mass fragmentation for compound **1**. Bottom: ASAP-HESI MSMS spectrum on *m/z* 379.1342 in positive HESI mode acquired on the Exactive Q mass spectrometer at HCD 35%.

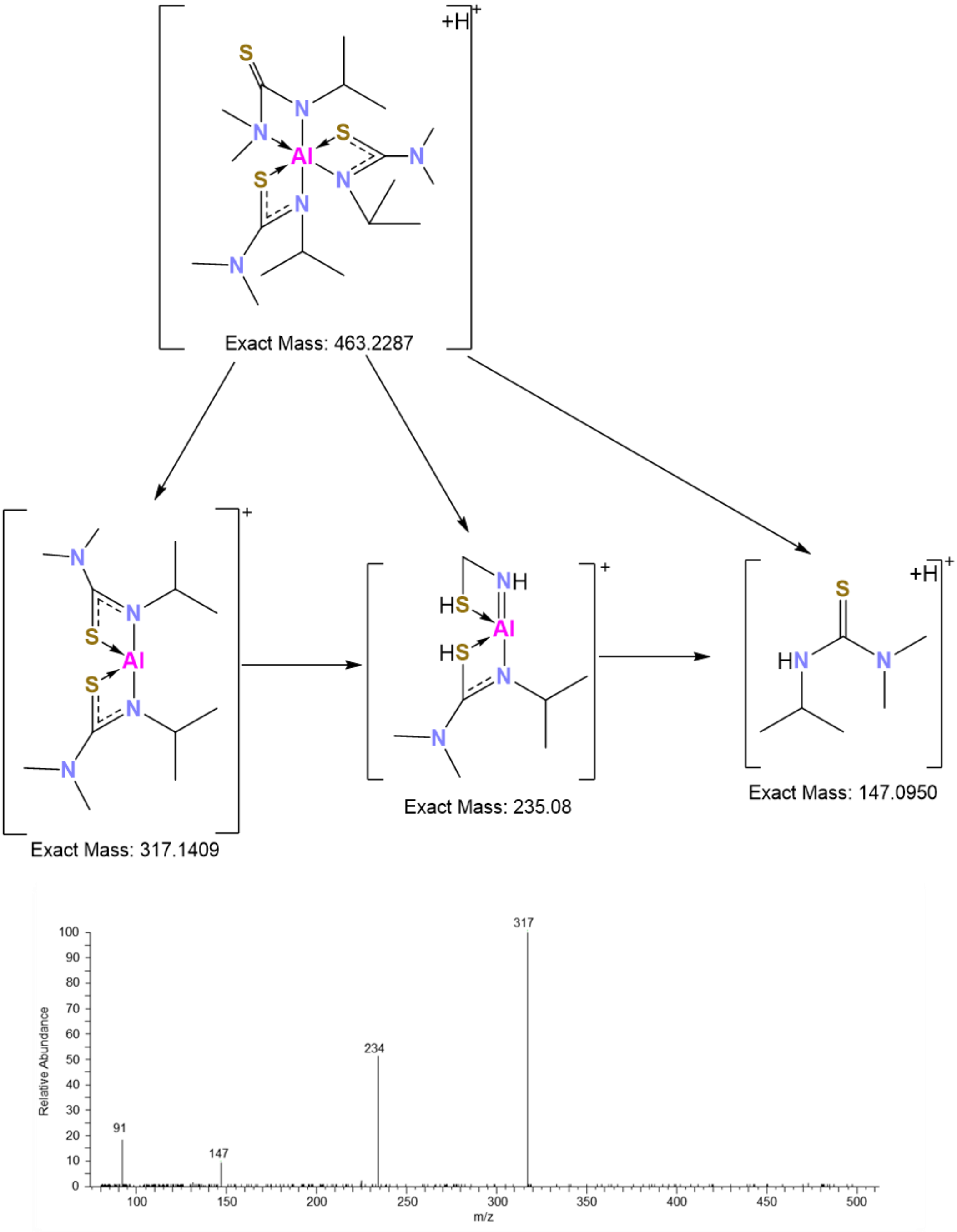

Figure S32: Top: Proposed mass fragmentation for compound **2**. Bottom: ASAP-HESI MSMS spectrum on *m/z* 463.2287 in positive HESI mode acquired on the Exactive Q mass spectrometer at HCD 35%.

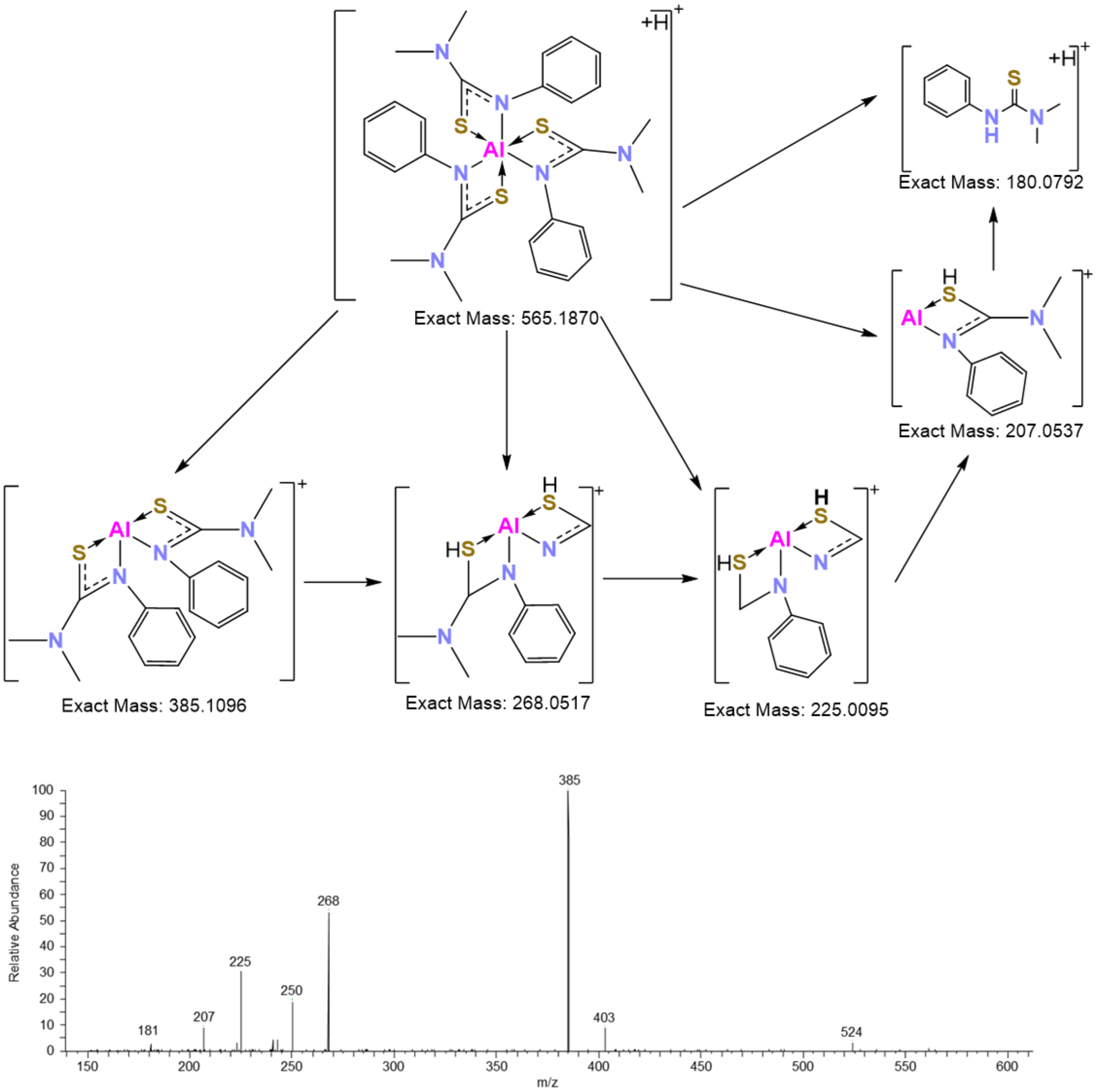

Figure S33: Top: Proposed mass fragmentation for compound **3**. Bottom: ASAP-HESI MSMS spectrum on *m/z* 565.1870 in positive HESI mode acquired on the Exactive Q mass spectrometer at HCD 35%.

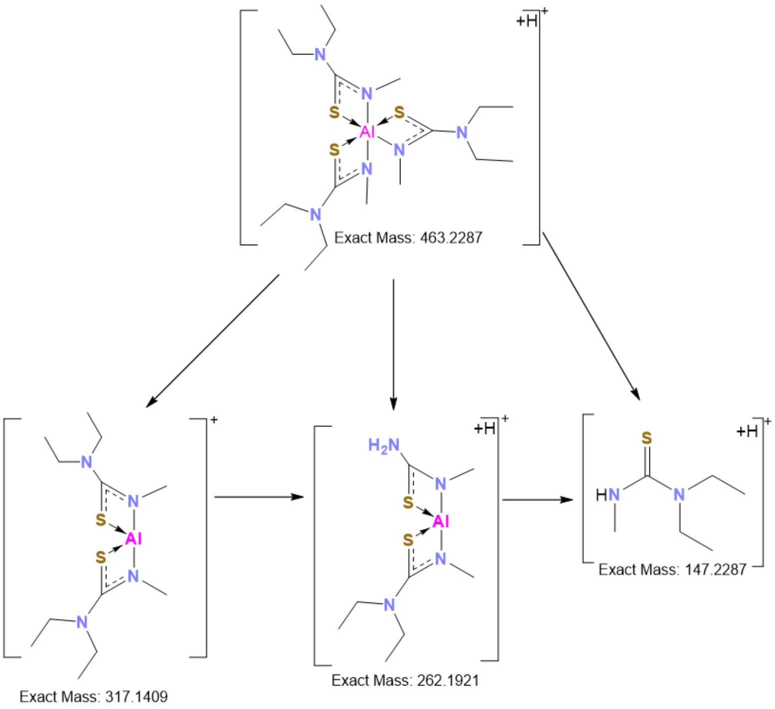

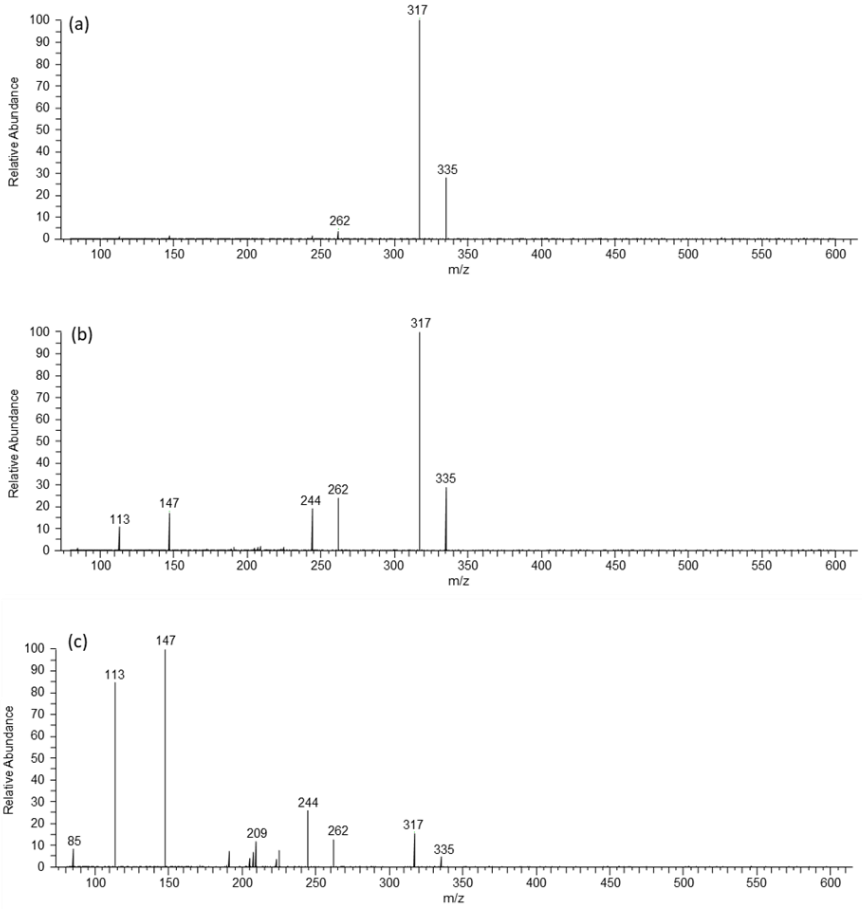

Figure S34: Top: Proposed mass fragmentation for compound **4**. Bottom: ASAP-HESI MSMS spectrum on *m/z* 463.2287 in positive HESI mode acquired on the Exactive Q mass spectrometer at HCD (a) 25% (b) 35% and (c) 50%.

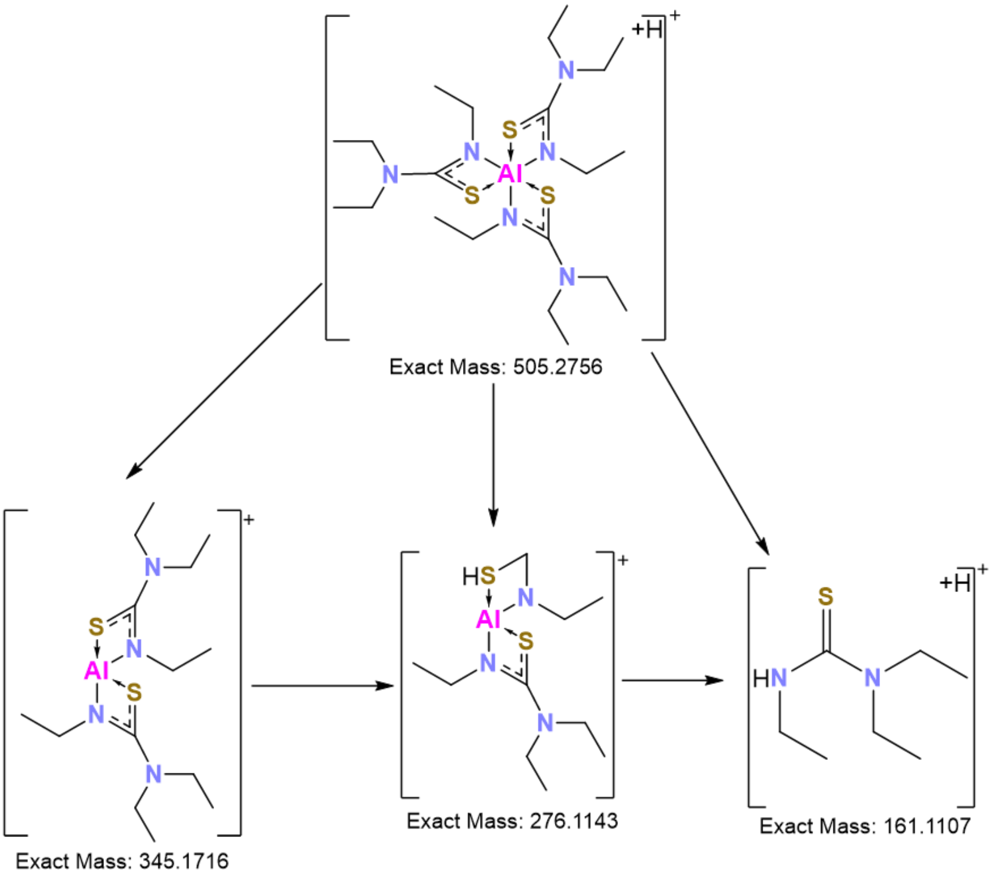

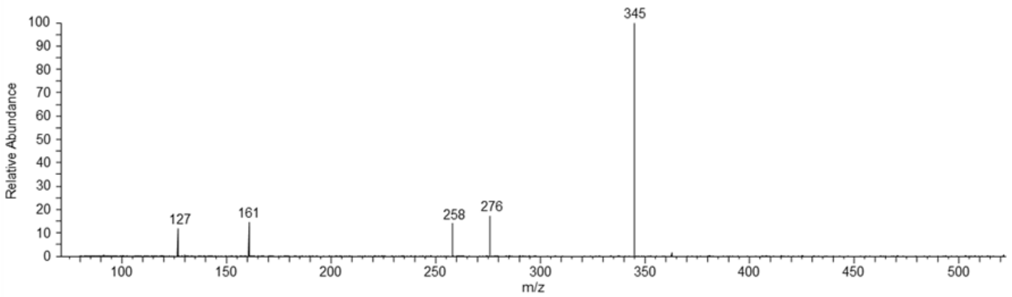

Figure S35: Top: Proposed mass fragmentation for compound **5**. Bottom: ASAP-HESI MSMS spectrum on *m/z* 505.2756 in positive HESI mode acquired on the Exactive Q mass spectrometer at HCD 35%.

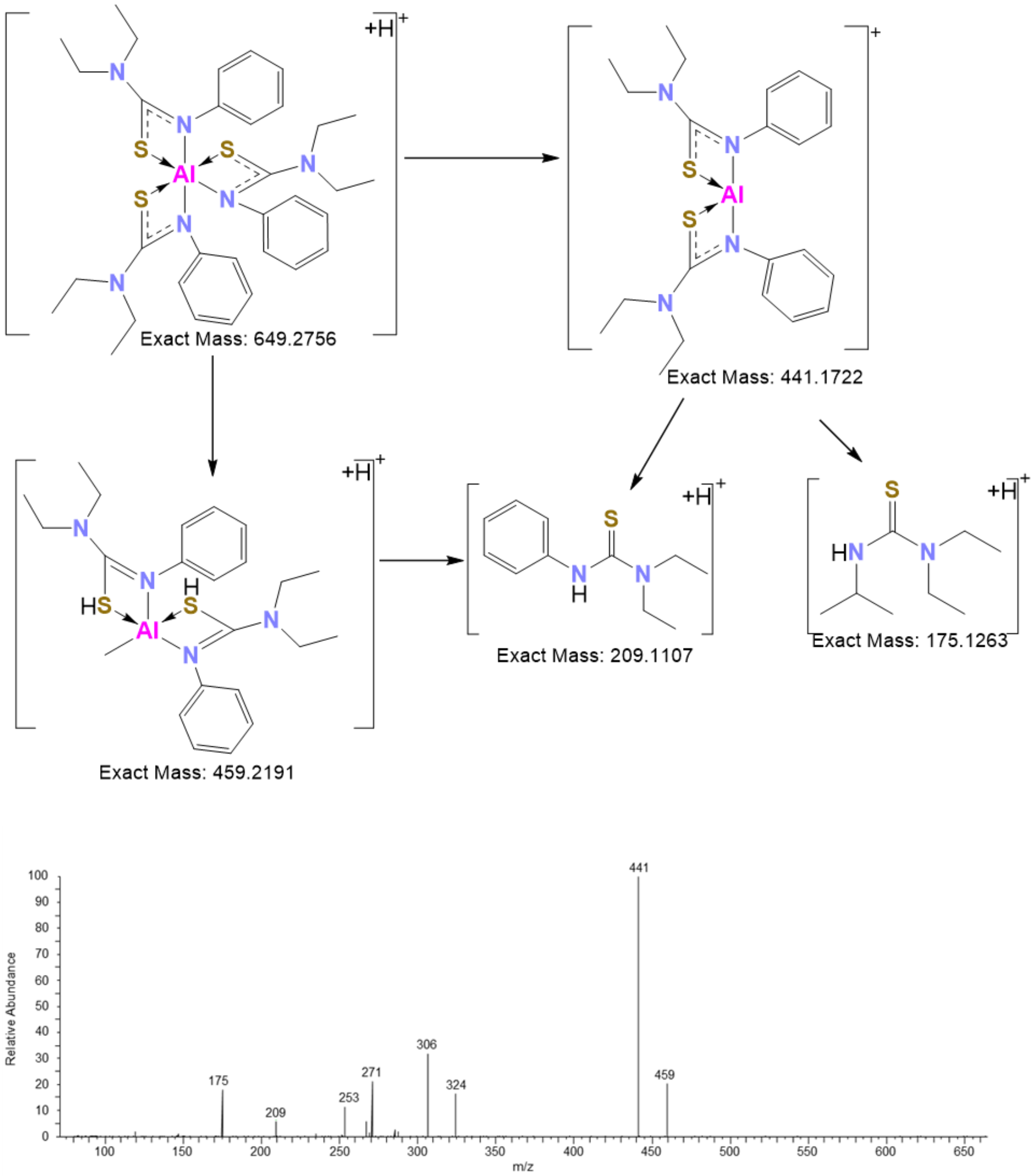

Figure S36: Top: Proposed mass fragmentation for compound **7**. Bottom: ASAP-HESI MSMS spectrum on *m/z* 649.2756 in positive HESI mode acquired on the Exactive Q mass spectrometer at HCD 35%.

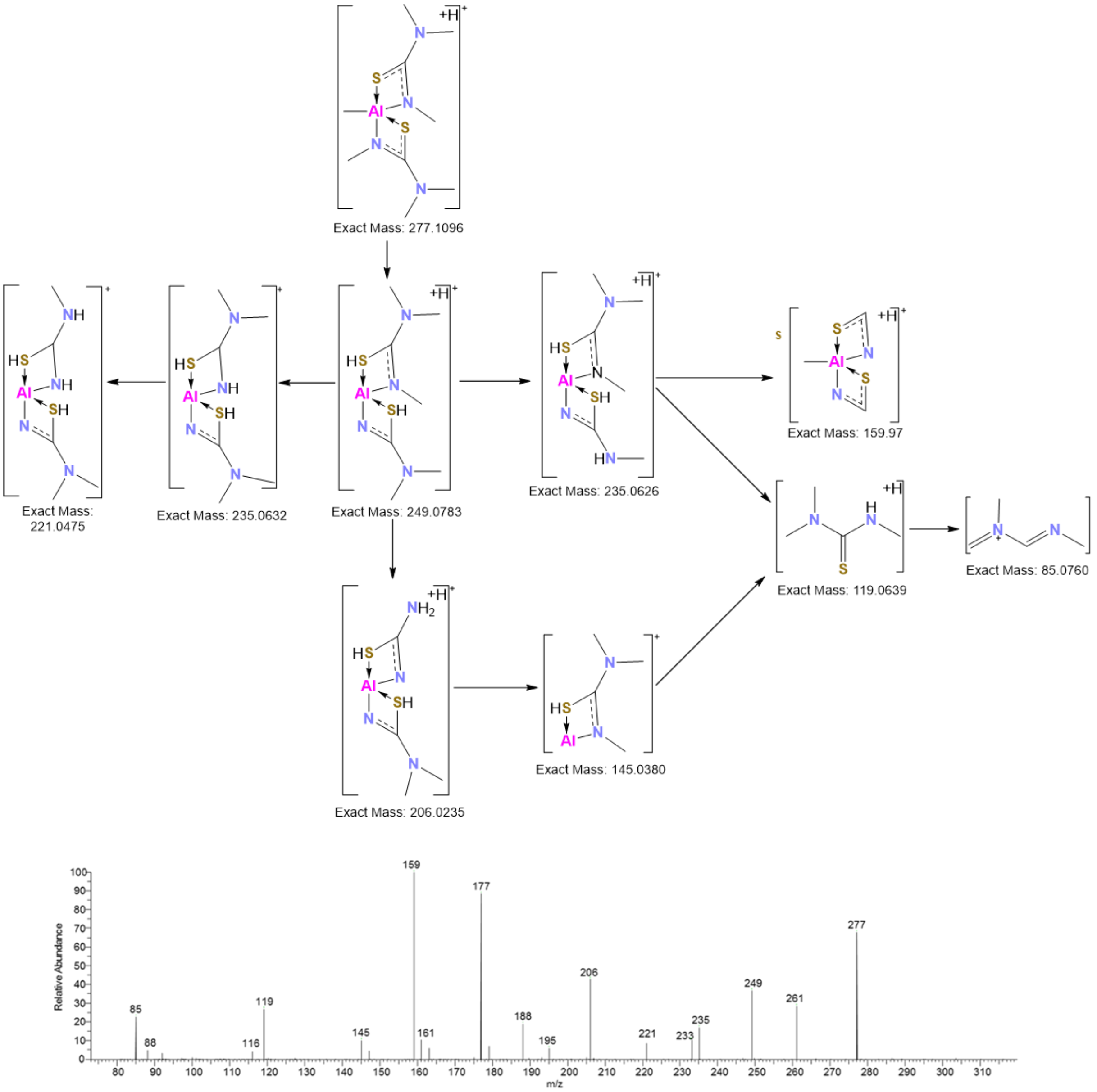

Figure S37: Top: Proposed mass fragmentation for compound **8**. Bottom: ASAP-HESI MSMS spectrum on *m/z* 277.1096 in positive HESI mode acquired on the Exactive Q mass spectrometer at HCD 35%.

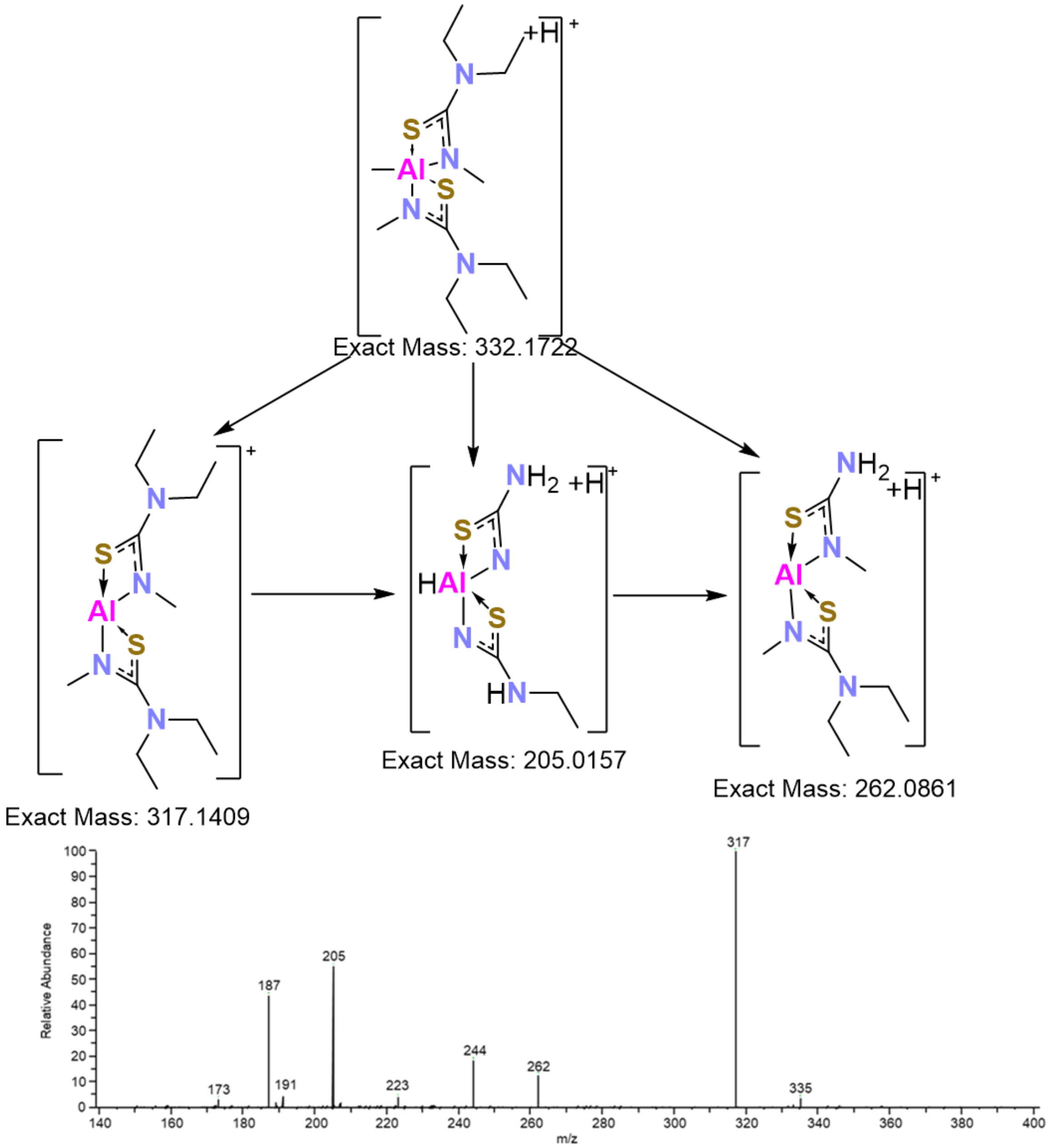

Figure S38: Top: Proposed mass fragmentation for compound **9**. Bottom: ASAP-HESI MSMS spectrum on *m/z* 332.1722 in positive HESI mode acquired on the Exactive Q mass spectrometer at HCD 35%.

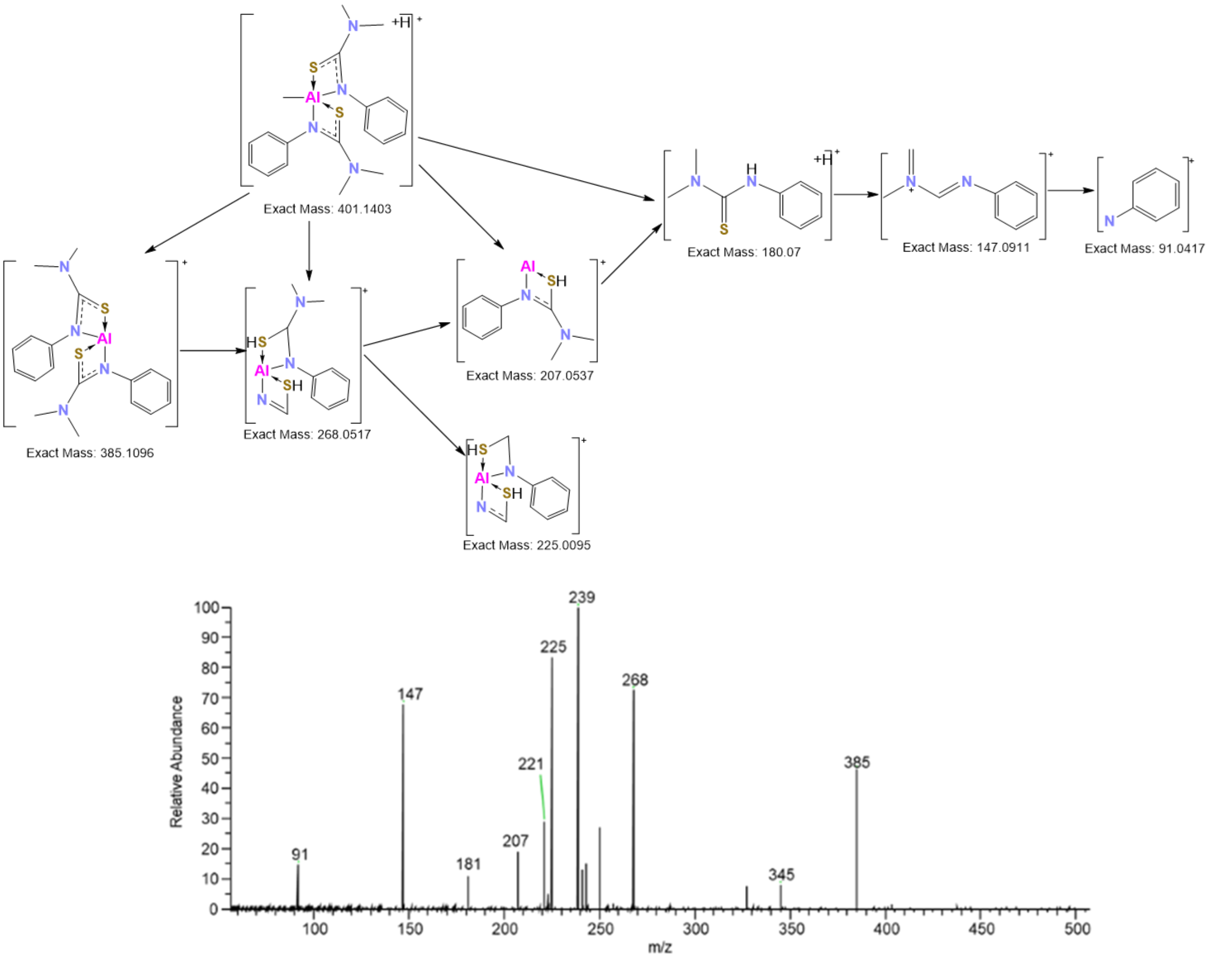

Figure S39: Top: Proposed mass fragmentation for compound **10**. Bottom: ASAP-HESI MSMS spectrum on *m/z* 401.1403 in positive HESI mode acquired on the Exactive Q mass spectrometer at HCD 35%.

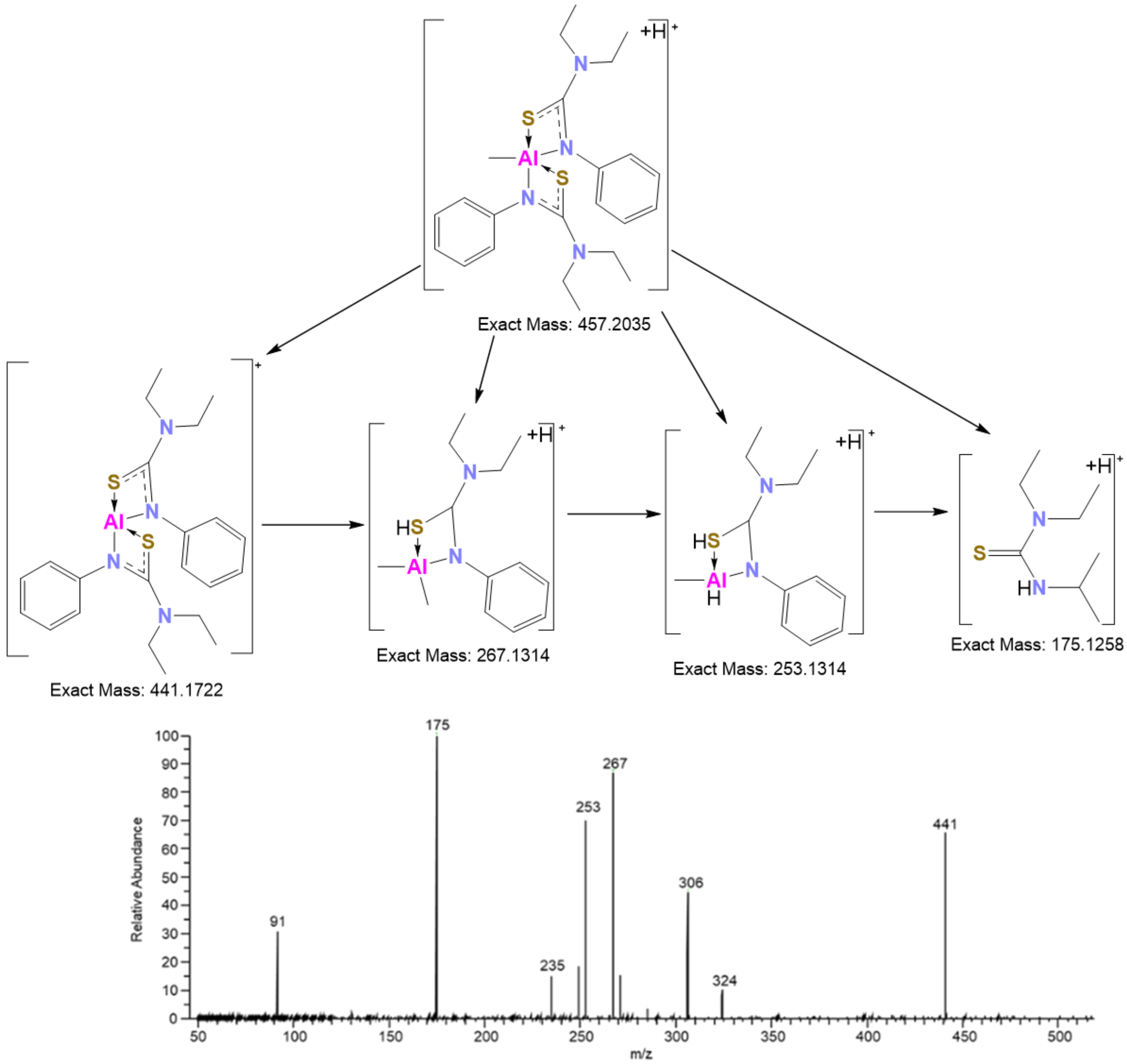

Figure S40: Top: Proposed mass fragmentation for compound **11**. Bottom: ASAP-HESI MSMS spectrum on *m/z* 457.2035 in positive HESI mode acquired on the Exactive Q mass spectrometer at HCD 35%.

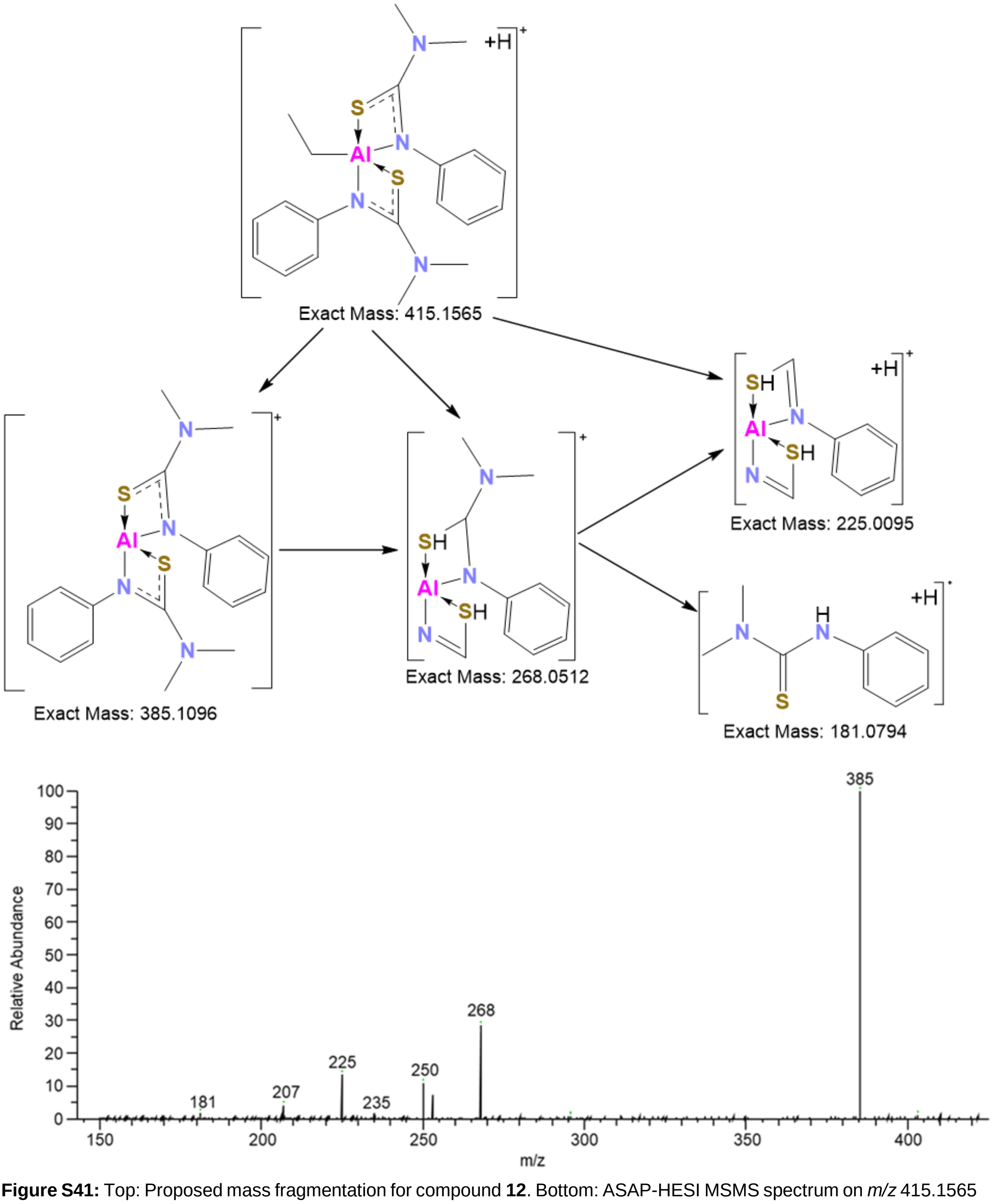

Figure S41: Top: Proposed mass fragmentation for compound **12**. Bottom: ASAP-HESI MSMS spectrum on *m/z* 415.1565 in positive HESI mode acquired on the Exactive Q mass spectrometer at HCD 35%.

5. TGA Plots

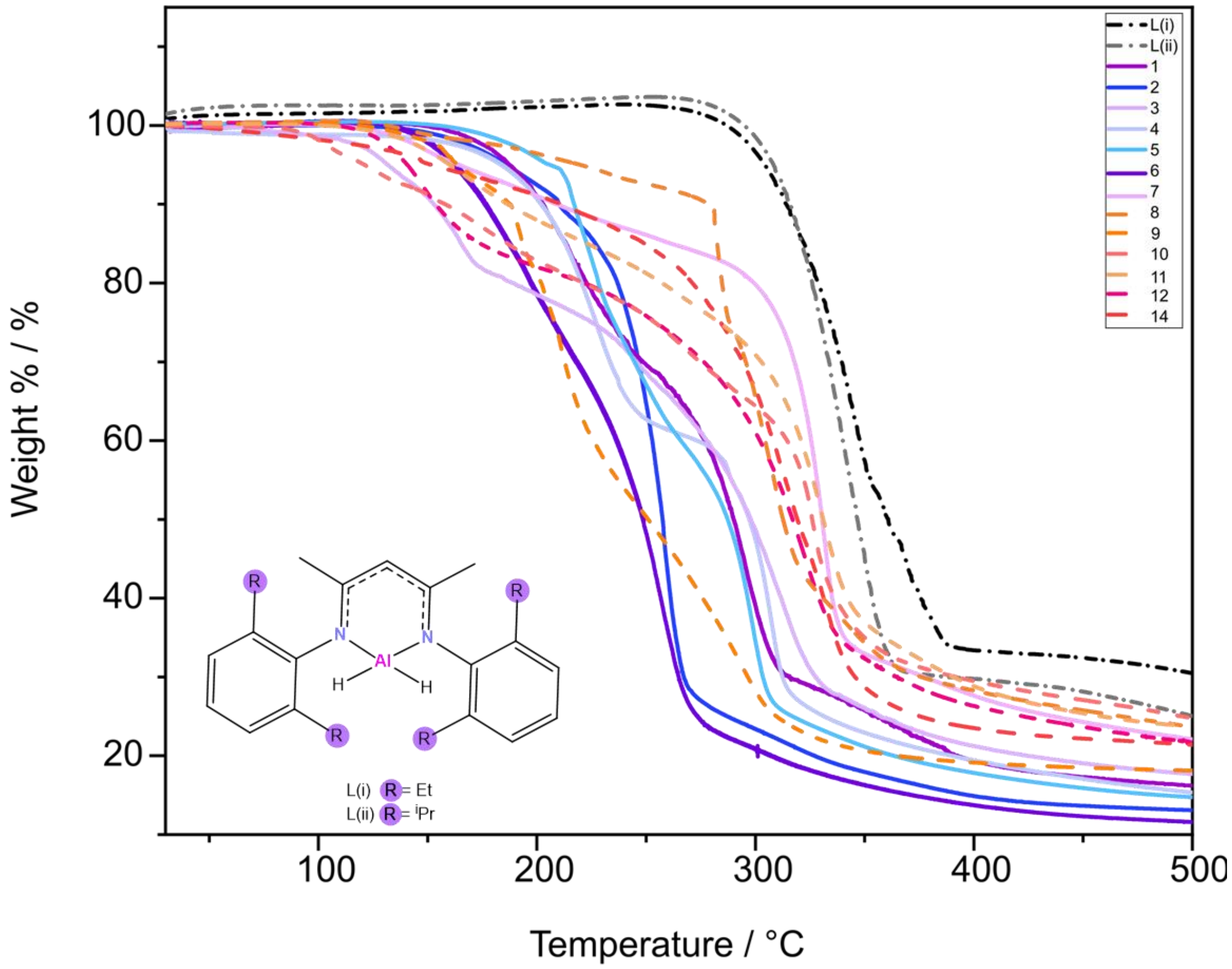

Figure S42: Overlapping thermograms of (a) Short dash and dot in shades of black: literature compounds L(i) and L(ii) (shown in inset), (b) Solid lines in shades of blue and purple: tris(thioureide) aluminium(III) compounds (**1** – **7**) and (c): Dashed lines in shades of red and orange: bis(thioureide) alkyl aluminium(III) compounds (**8** – **12** and **14**).

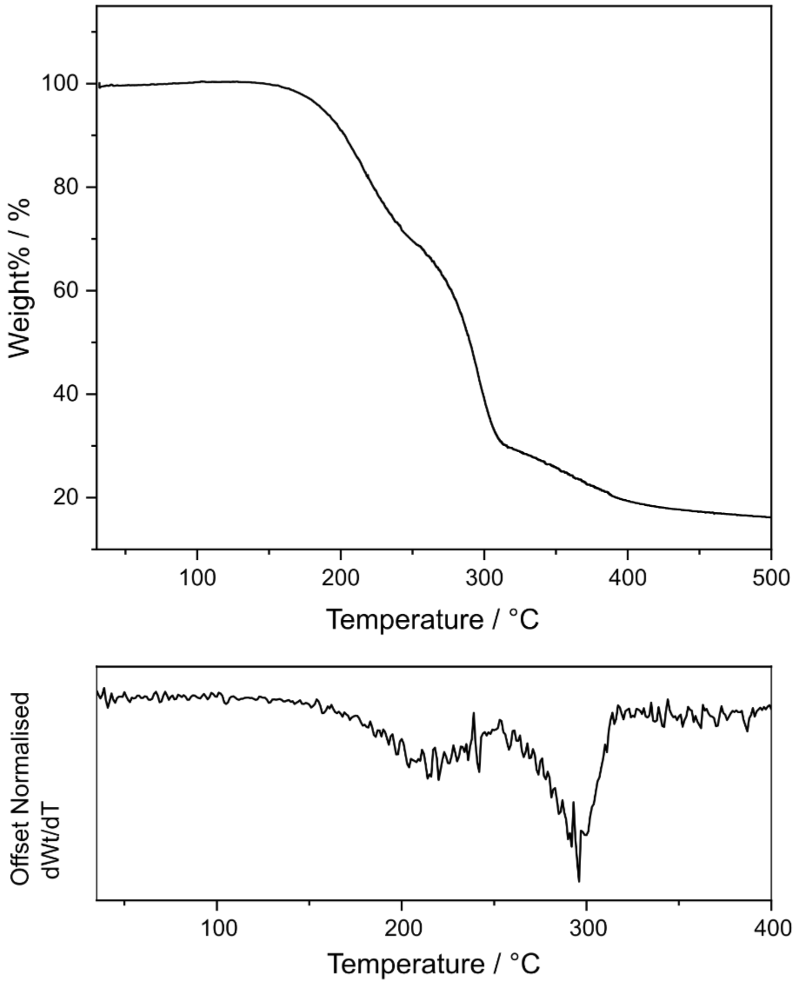

Figure S43: Top: TGA plot of compound **1**; Bottom: Offset derivative of the thermogram with dWt/dT = 0.

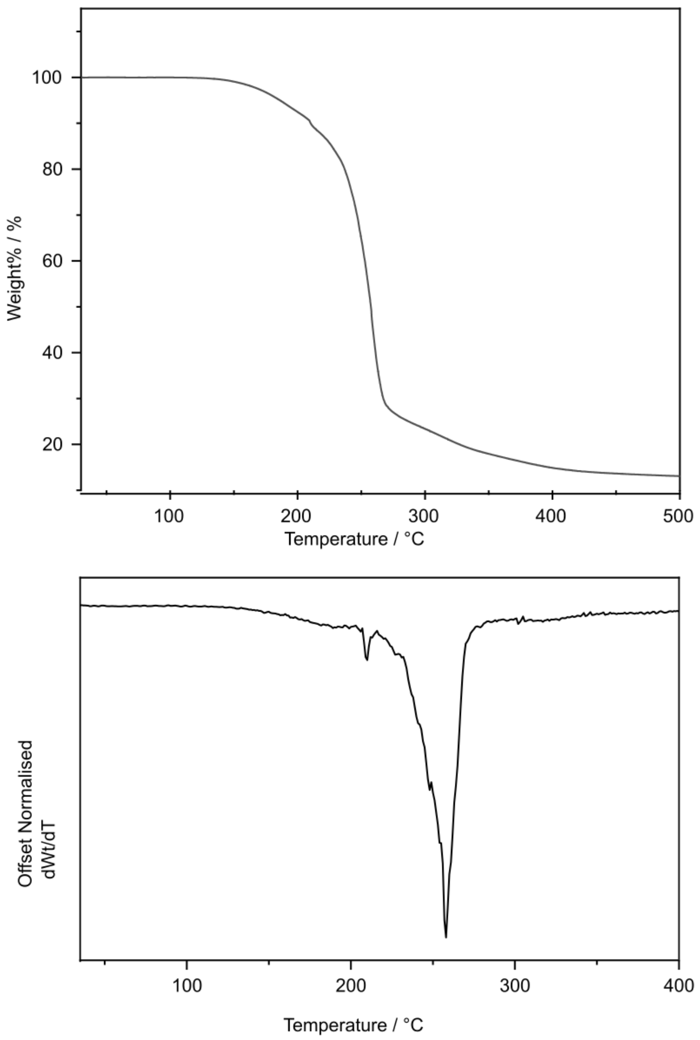

Figure S44: Top: TGA plot of compound **2**; Bottom: Offset derivative of the thermogram with dWt/dT = 0.

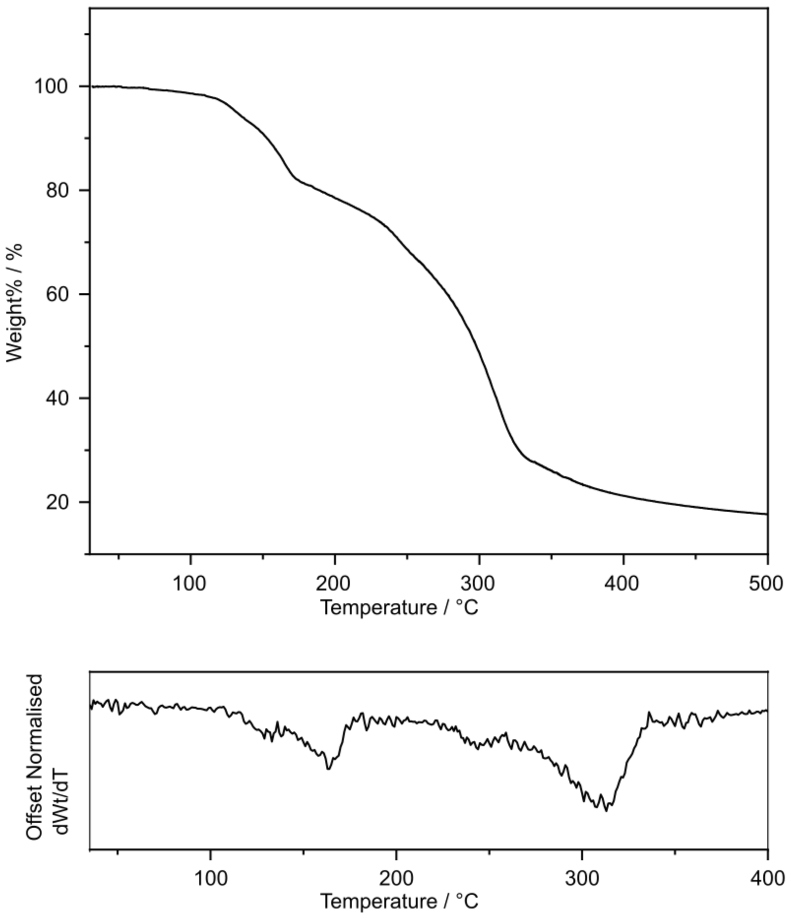

Figure S45: Top: TGA plot of compound **3**; Bottom: Offset derivative of the thermogram with dWt/dT = 0.

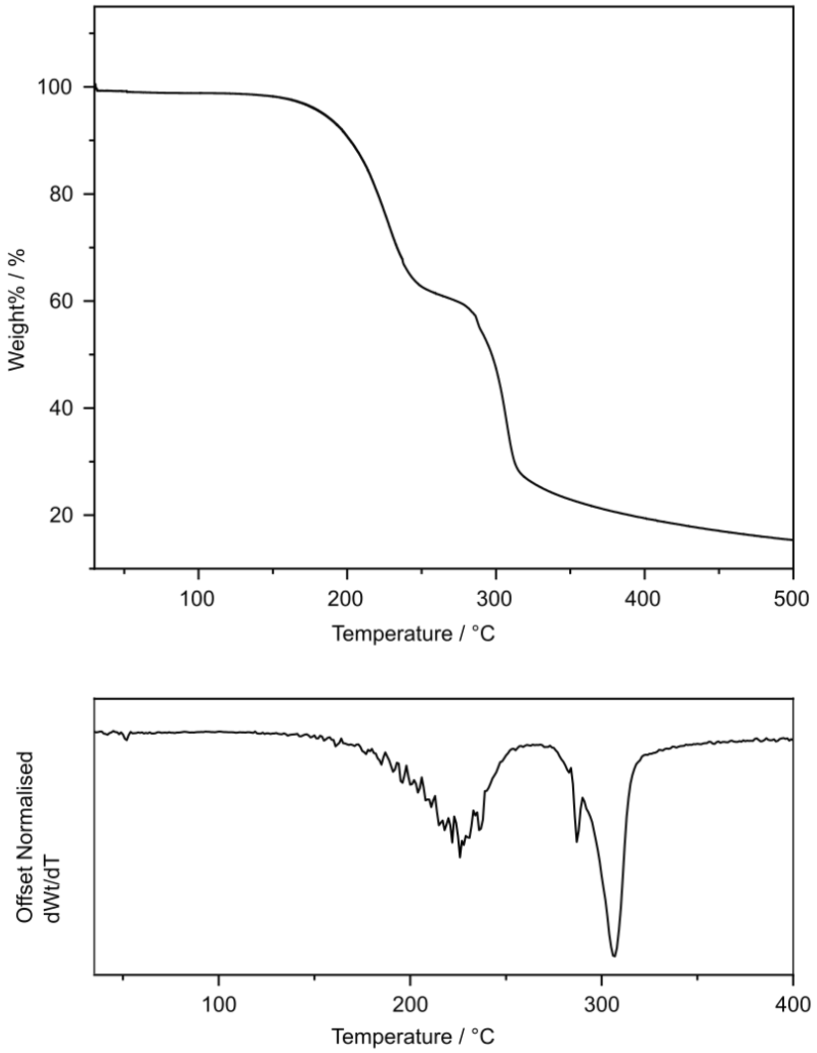

Figure S46: Top: TGA plot of compound **4**; Bottom: Offset derivative of the thermogram with dWt/dT = 0.

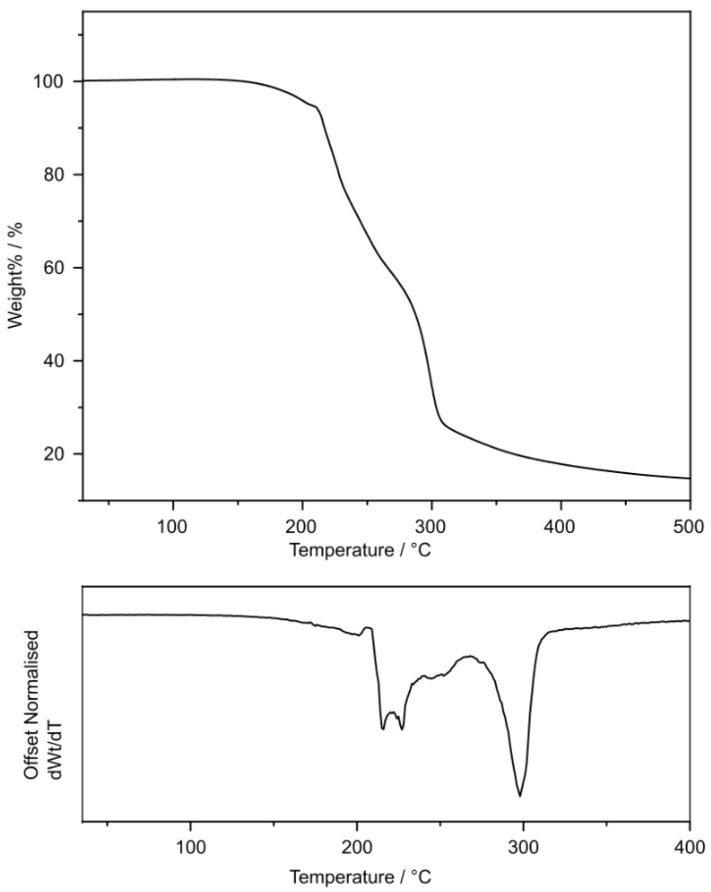

Figure S47: Top: TGA plot of compound **5**; Bottom: Offset derivative of the thermogram with dWt/dT = 0.

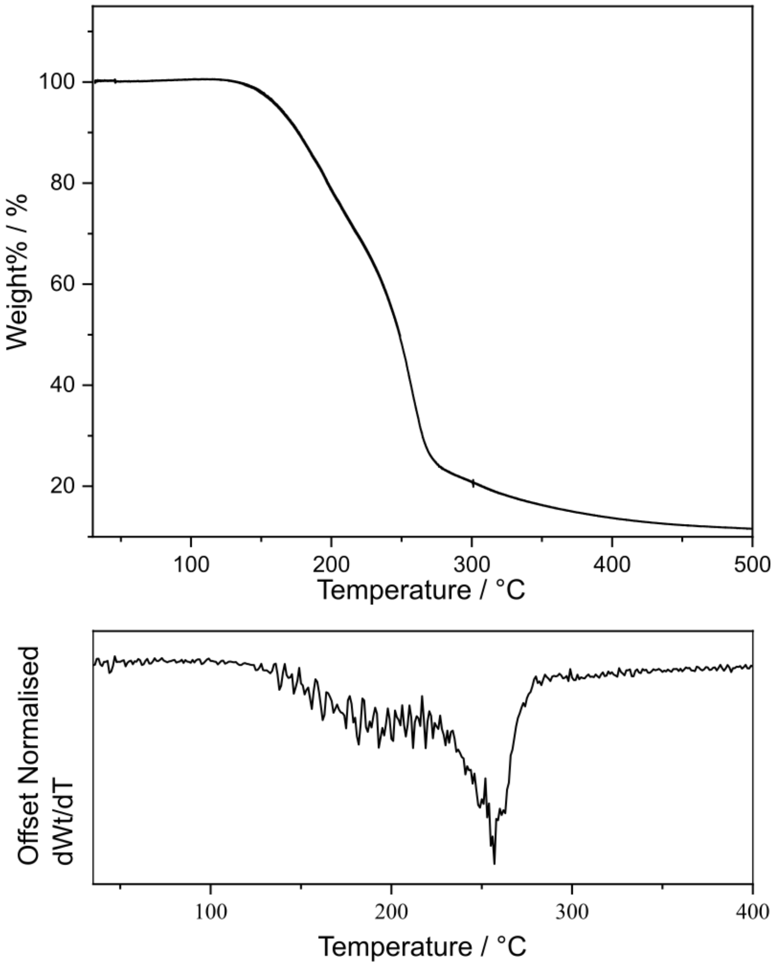

Figure S48: Top: TGA plot of compound **6**; Bottom: Offset derivative of the thermogram with dWt/dT = 0.

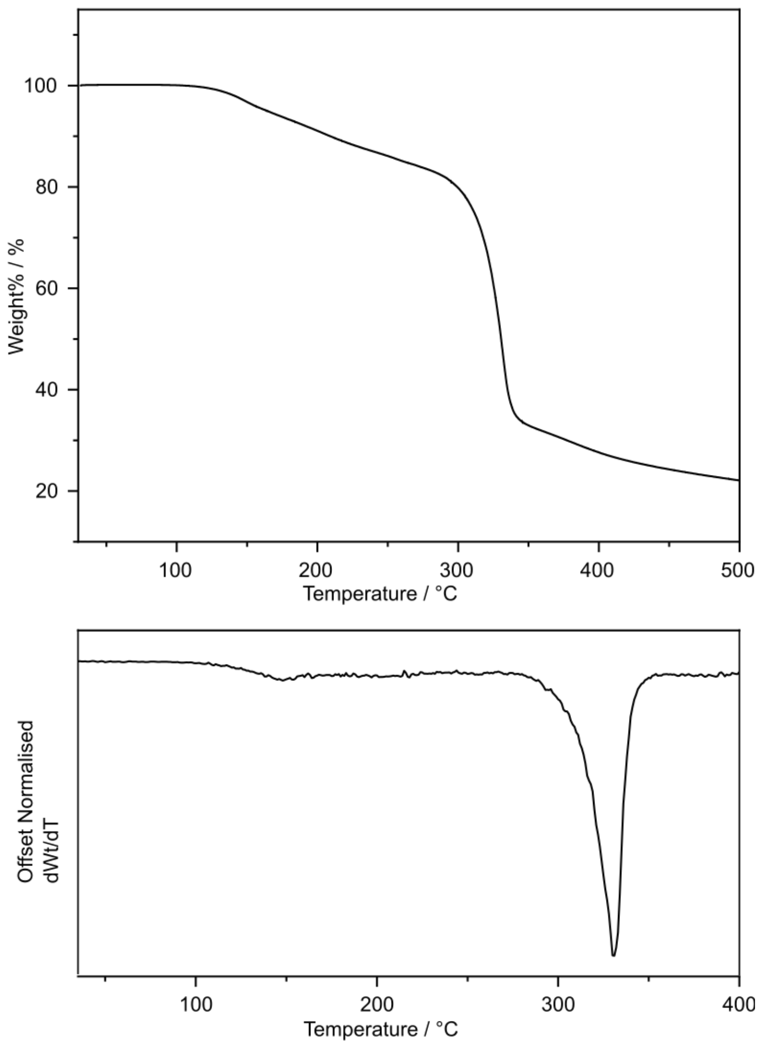

Figure S49: Top: TGA plot of compound **7**; Bottom: Offset derivative of the thermogram with dWt/dT = 0.

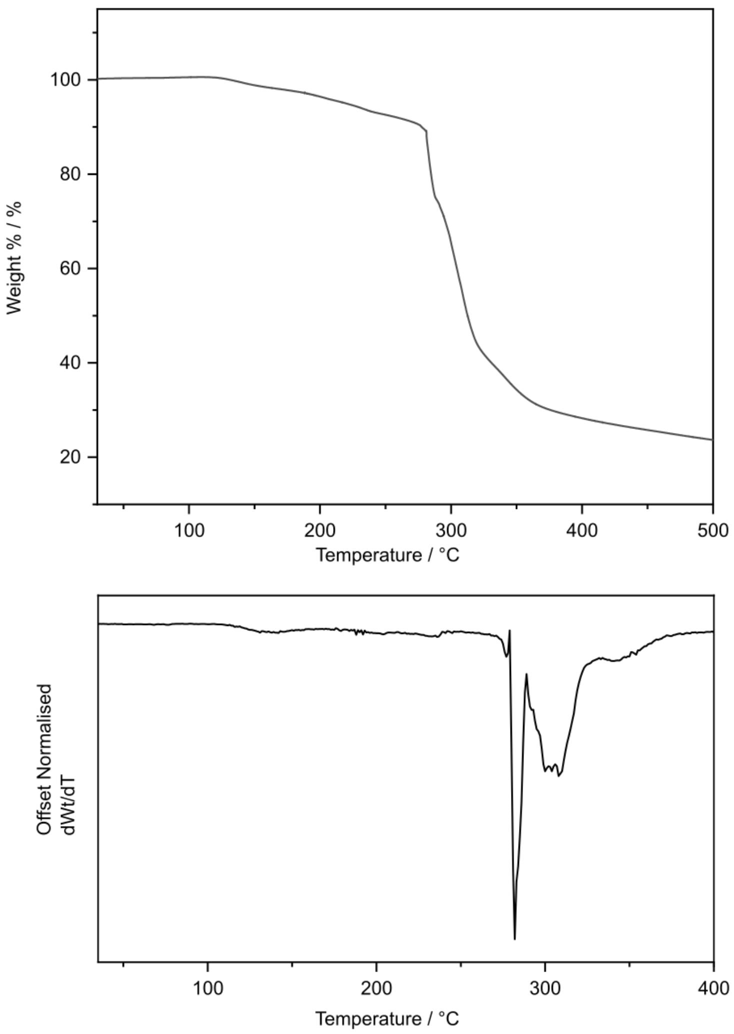

Figure S50: Top: TGA plot of compound **8**; Bottom: Offset derivative of the thermogram with dWt/dT = 0.

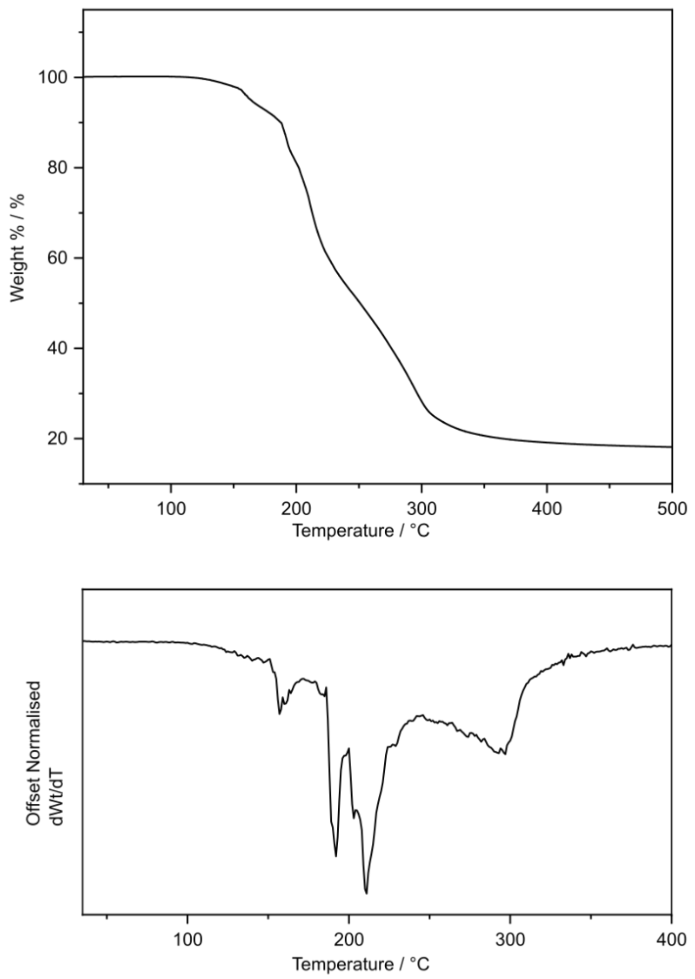

Figure S51: Top: TGA plot of compound **9**; Bottom: Offset derivative of the thermogram with dWt/dT = 0.

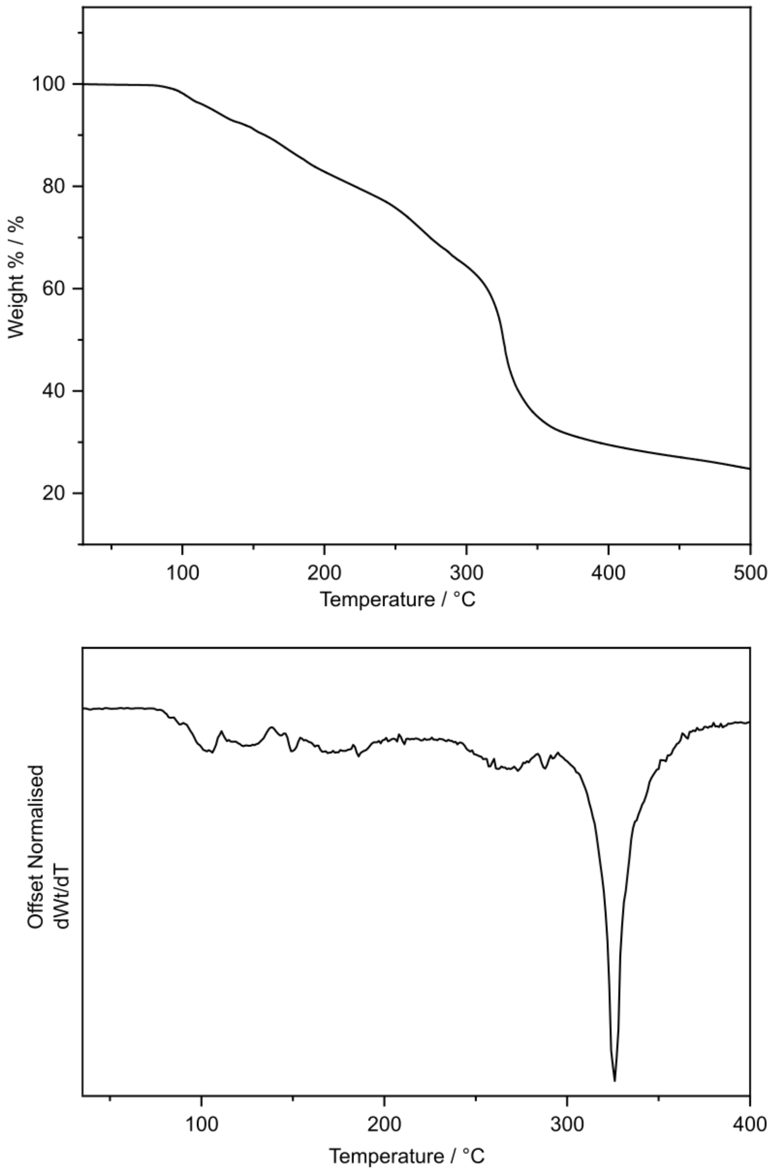

Figure S52: Top: TGA plot of compound **10**; Bottom: Offset derivative of the thermogram with dWt/dT = 0.

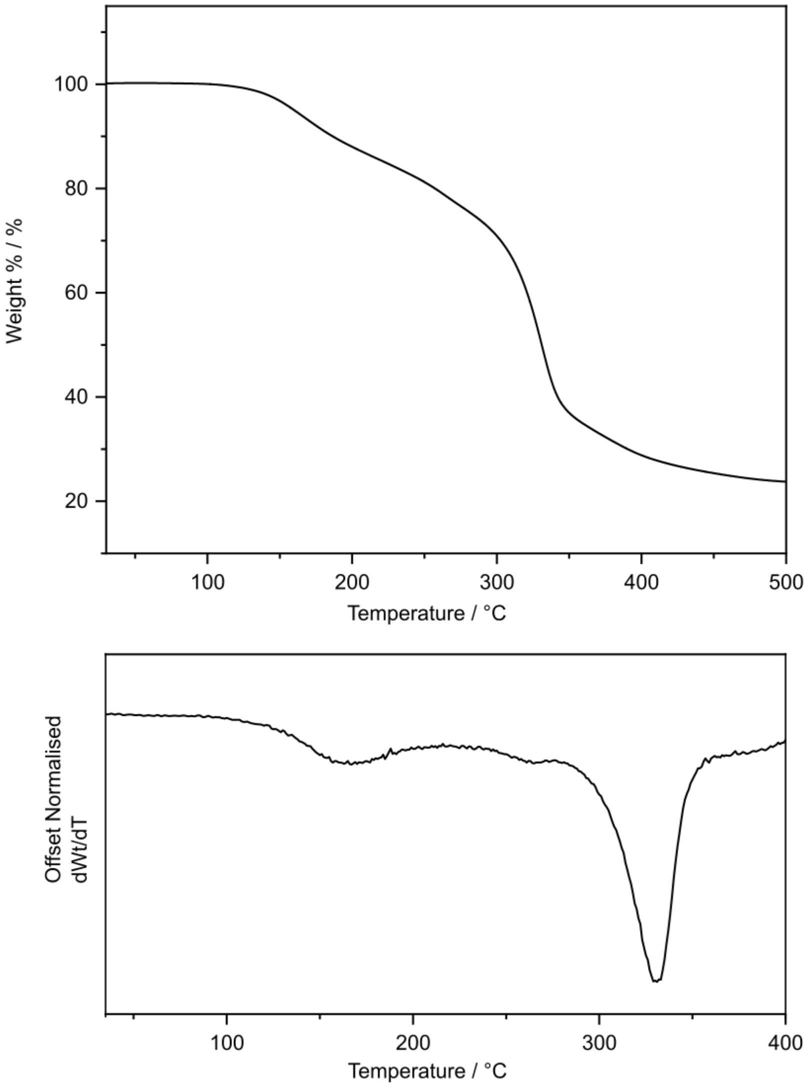

Figure S53: Top: TGA plot of compound **11**; Bottom: Offset derivative of the thermogram with dWt/dT = 0.

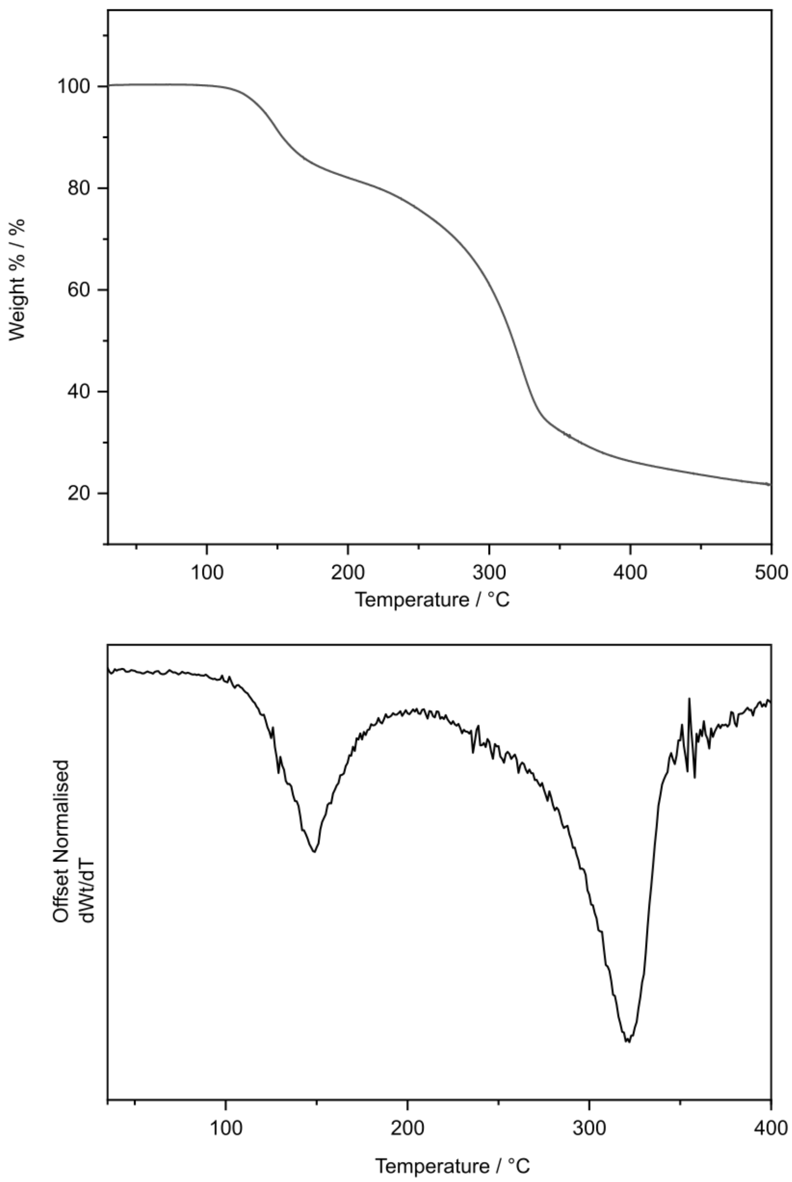

Figure S54: Top: TGA plot of compound **12**; Bottom: Offset derivative of the thermogram with dWt/dT = 0.

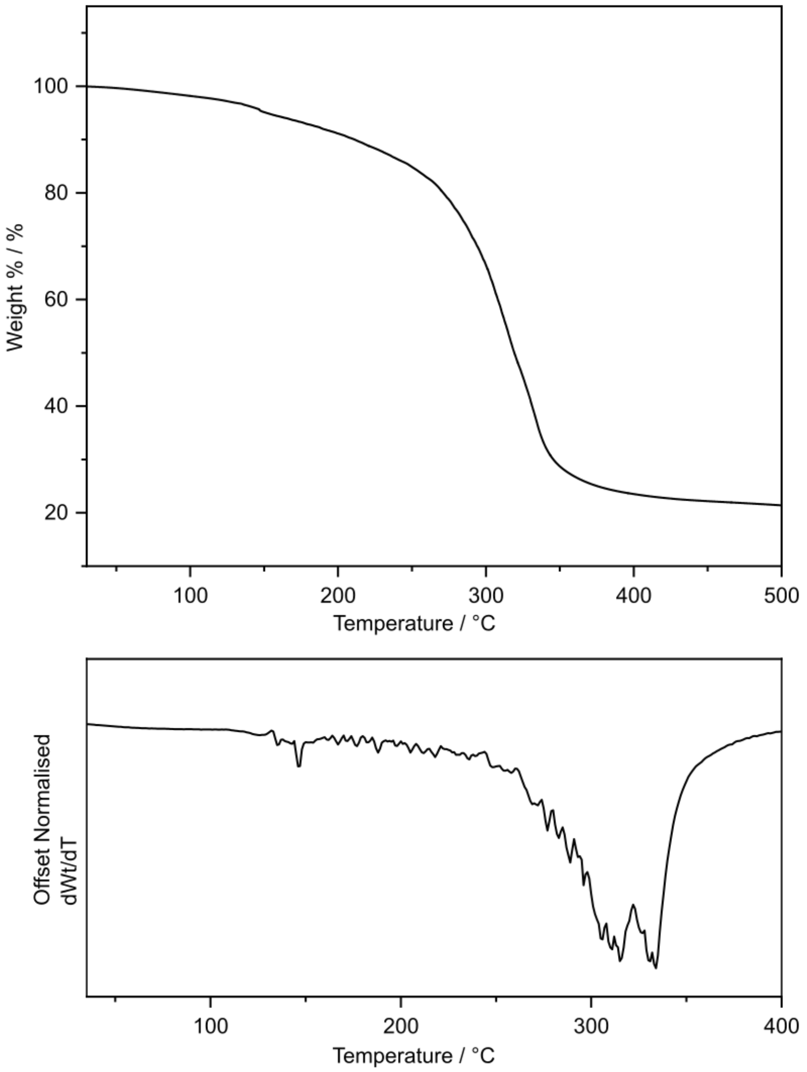

Figure S55: Top: TGA plot of compound **14**; Bottom: Offset derivative of the thermogram with dWt/dT = 0.
